# Supplementary material for: Human sensorimotor organoids derived from healthy and amyotrophic lateral sclerosis stem cells form neuromuscular junctions
Source: Nat Commun. 2021 Aug 6;12:4744. doi: 10.1038/s41467-021-24776-4 (PMC8346474; doi:10.1038/s41467-021-24776-4)
Supplement: Supplementary file 1 — Supplementary information [file 41467_2021_24776_MOESM1_ESM.pdf]

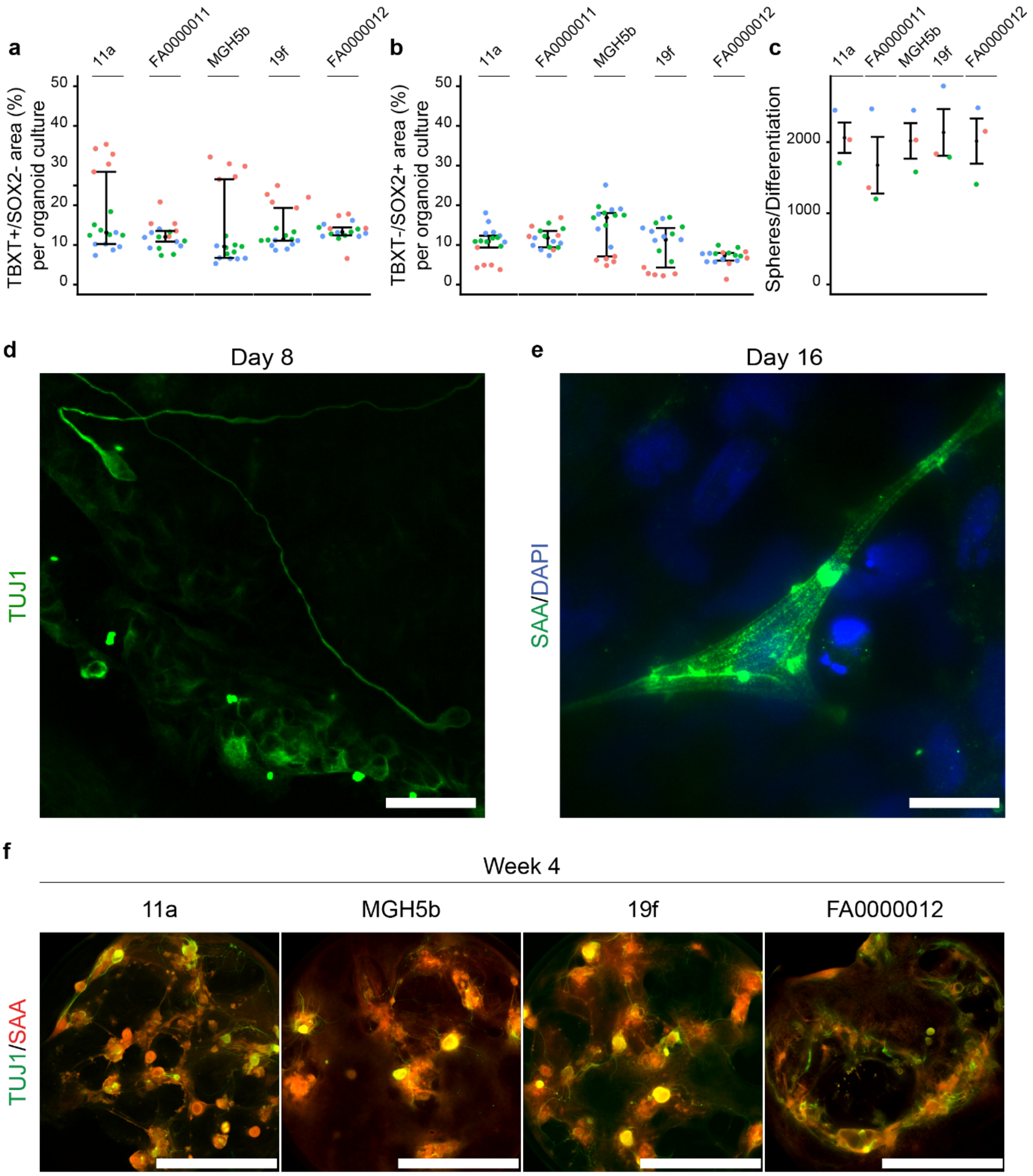

Supplementary Fig. 1 | Spheres are generated in consistent numbers from iPSC lines and give rise to neurons and myocytes. a,b Quantification of TBXT+/SOX2- (**a**) and TBXT-/SOX2+ (**b**) staining area after two days in culture. Bars indicate median and I.Q.R. Control lines: 11a, FA0000011; ALS lines: MGH5b, 19f, and FA0000012. (*n* = 17 organoid cultures obtained from three independent biological differentiation replicates, distinct colors). **c,**

Number of spheres resulting from three independent differentiations of each of five iPSC lines. Bars represent mean and SEM. (*n* = 3 independent biological differentiation replicates, distinct colors; one-way ANOVA, F=0.758, P=0.666). **d,** Scattered TUJ1+ cells at day eight of culture. Scale bar, 50 µm. **e,** SAA+ myocyte with typical morphology at day 16 of culture. Scale bar, 50 µm. **f**, Whole well epifluorescence examples of organoid cultures labeled for TUJ1 and SAA at four weeks of culture. Scale bar, 7.8mm.

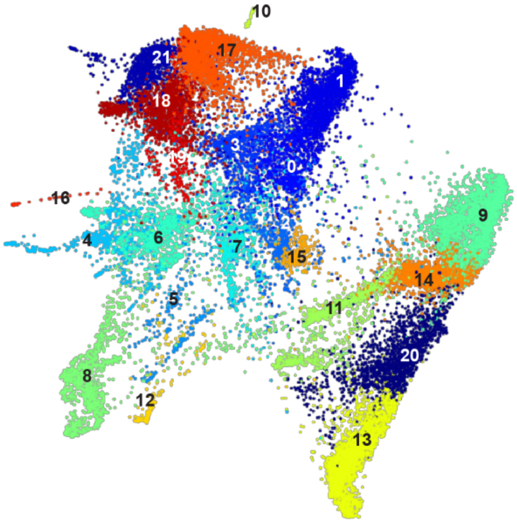

Supplementary Fig. 2 | Organoid overview and SPRING generated clusters. SPRING analysis of scRNA-seq data shows a similar number and type of clusters as Seurat analysis.

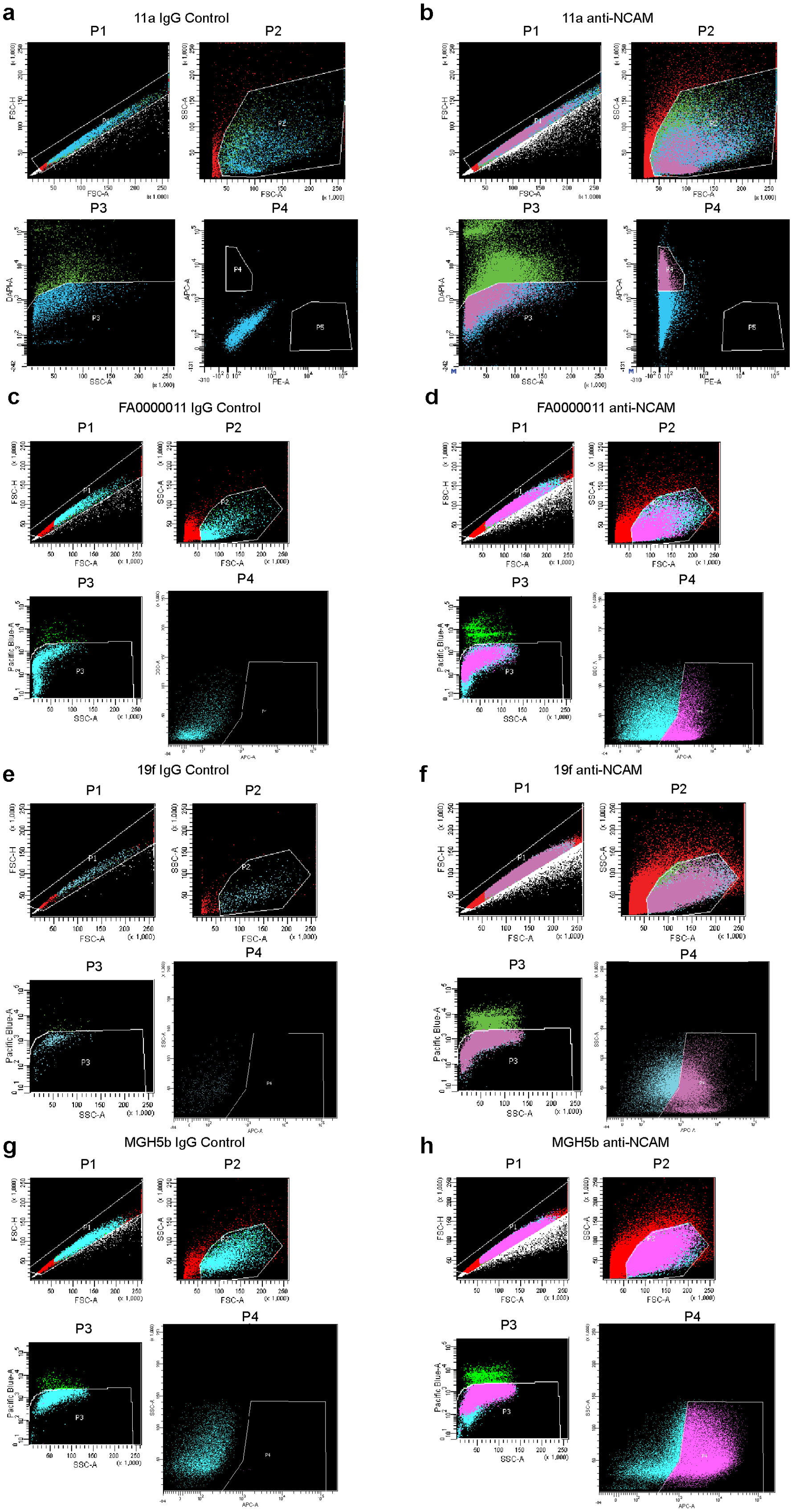
a
11a IgG Control
b
11a anti-NCAM
c
FA0000011 IgG Control
d
FA0000011 anti-NCAM
e
19f IgG Control
f
19f anti-NCAM
g
MGH5b IgG Control
h
MGH5b anti-NCAM
P1
P2
P3
P4
P5
FSC-H
FSC-A
SSC-A
DAPI-A
APC-A
PE-A
Pacific Blue-A
(x 1,000)

Supplementary Fig. 3 | FACS gating for NCAM cell isolation. Gates used for FACS sorting of NCAM expressing cells from differentiation of four iPSC lines, at four weeks of organoid culture. (**a, c, e, g)** Fraction of the sample labeled with a negative control antibody (a non-specific antibody matching the specific IgG isotype), showing the selection of gates used for excluding the population of cells negative for NCAM expression. Forward scatter height (FSC-H) and forward scatter area (FSC-A) were used for doublet exclusion and forward scatter area (FSC-A) and side scatter area (SSC-A) were used to exclude cell debris. (**b, d, f, h)** NCAM-APC labeled samples showing an increase in the labeled population (P4) when stained with an anti-NCAM antibody compared to the controls. Gate panels (P4) match the data presented in Fig. 3.

a

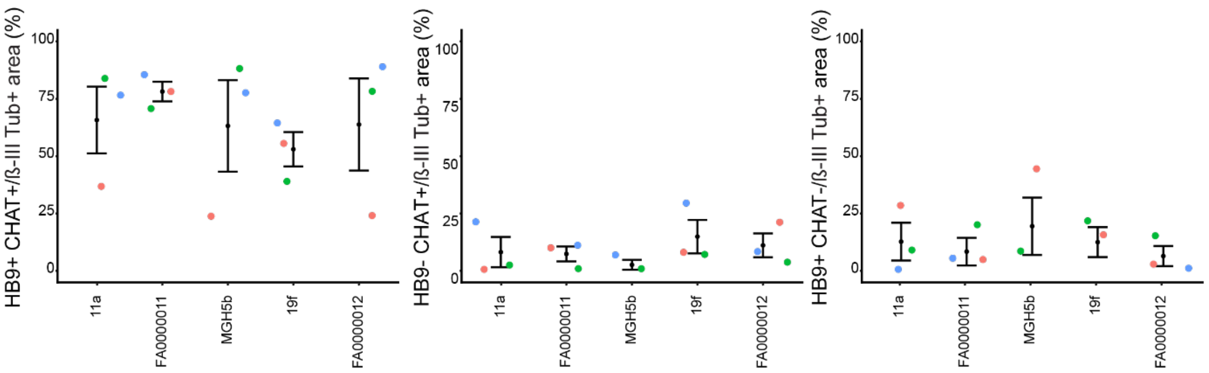

Supplementary Fig. 4 | Presence of Motor Neurons in Whole Organoid Cultures. a, Quantification of HB9+ ChAT+ (left), HB9- ChAT+ (middle), and HB9+ ChAT- (right) area of β-III Tubulin expressing cells, in an unbiased grid of thirty-five fields of view per whole organoid culture of five iPSC lines at four weeks (*n* = 3 independent biological differentiation replicates, distinct colors). Bars indicate mean and SEM.

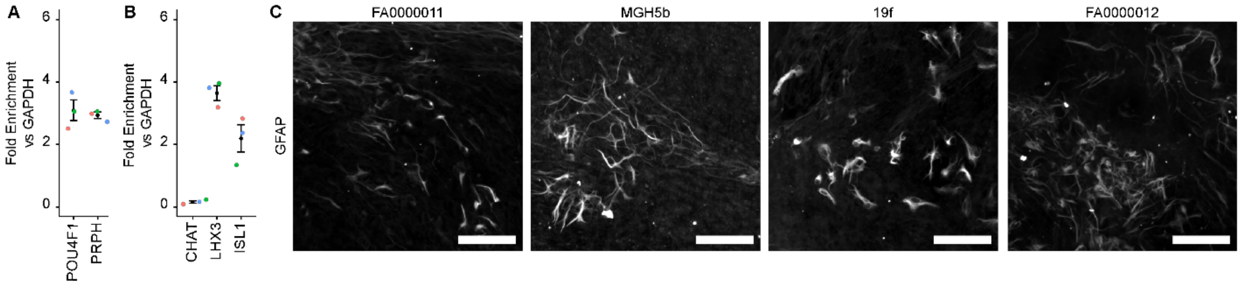

Supplementary Fig. 5 | Confirmation of Ectodermal Lineage Derivatives. a, Quantification of sensory neuron markers *POU4F1* and *PRPH* expression in organoid cultures by qPCR at week ten from one differentiation of three iPSC lines (11a, 19f, MGH5b). Bars indicate mean and SEM. **b,** Quantification of motor neuron markers ChAT, LHX3, and ISL1 by qPCR in cultures at week ten from one differentiation of three iPSC lines (11a, 19f, MGH5b). Bars indicate mean and SEM. **c,** Example of GFAP+ astrocytes in sensorimotor organoid cultures from four iPSC lines at eight weeks of culture. Data are representative from three independent biological differentiations for each of four iPSC lines. Scale bars represent 100 μm. Examples from the iPSC line 11a are in Fig. 3L.

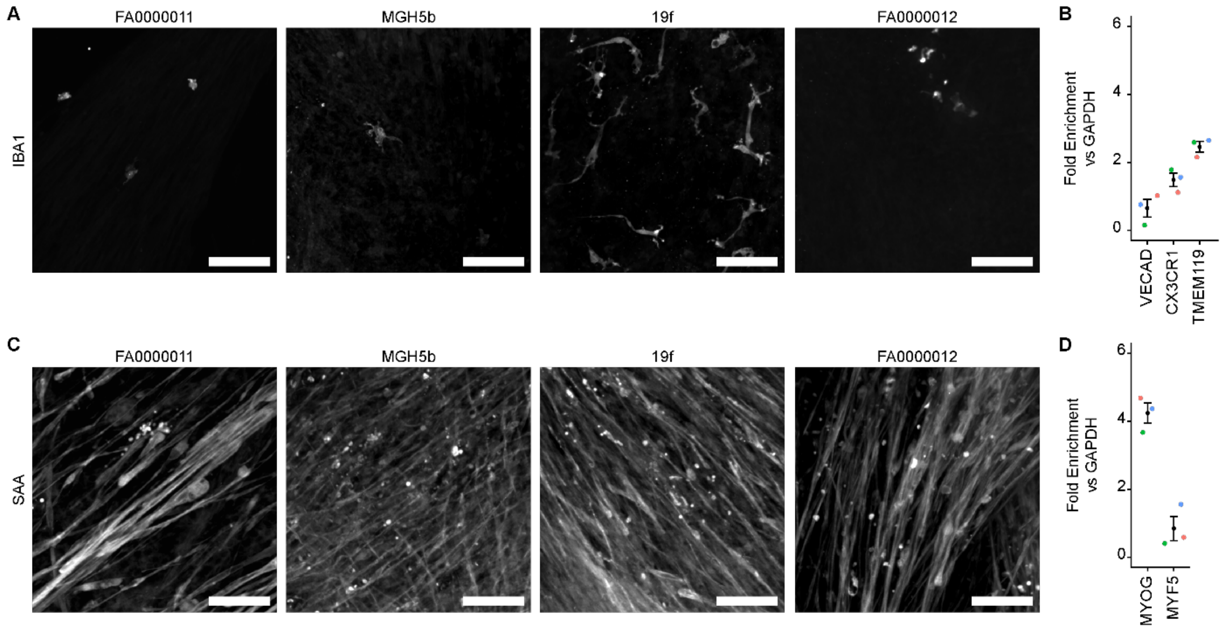

Supplementary Fig. 6 | Confirmation of Mesodermal Lineage Derivatives. a, Microglia labeled with IBA1 in organoid cultures from differentiation of four indicated iPSC lines at eight weeks of culture. Data are representative from three independent biological differentiations replicates for each of four iPSC lines. Scale bars represent 100 µm. **b,** Quantification of microglial (*CX3CR1* and *TMEM119*) and endothelial (*CDH5*, also known as VECAD) marker genes by qPCR at week ten from one differentiation of three iPSC lines (11a, 19f, MGH5b). Bars indicate mean and SEM. **c,** SAA+ skeletal muscle in sensorimotor organoids differentiated from four iPSC lines at eight weeks of culture, representative from three independent biological differentiation replicates for each of four iPSC lines. Scale bars represent 100 µm. **d,** Quantification of skeletal muscle markers *MYOG* and *MYF5* expression in organoid cultures by qPCR at week ten from one differentiation of three iPSC lines (11a, 19f, MGH5b). Bars indicate mean and SEM.

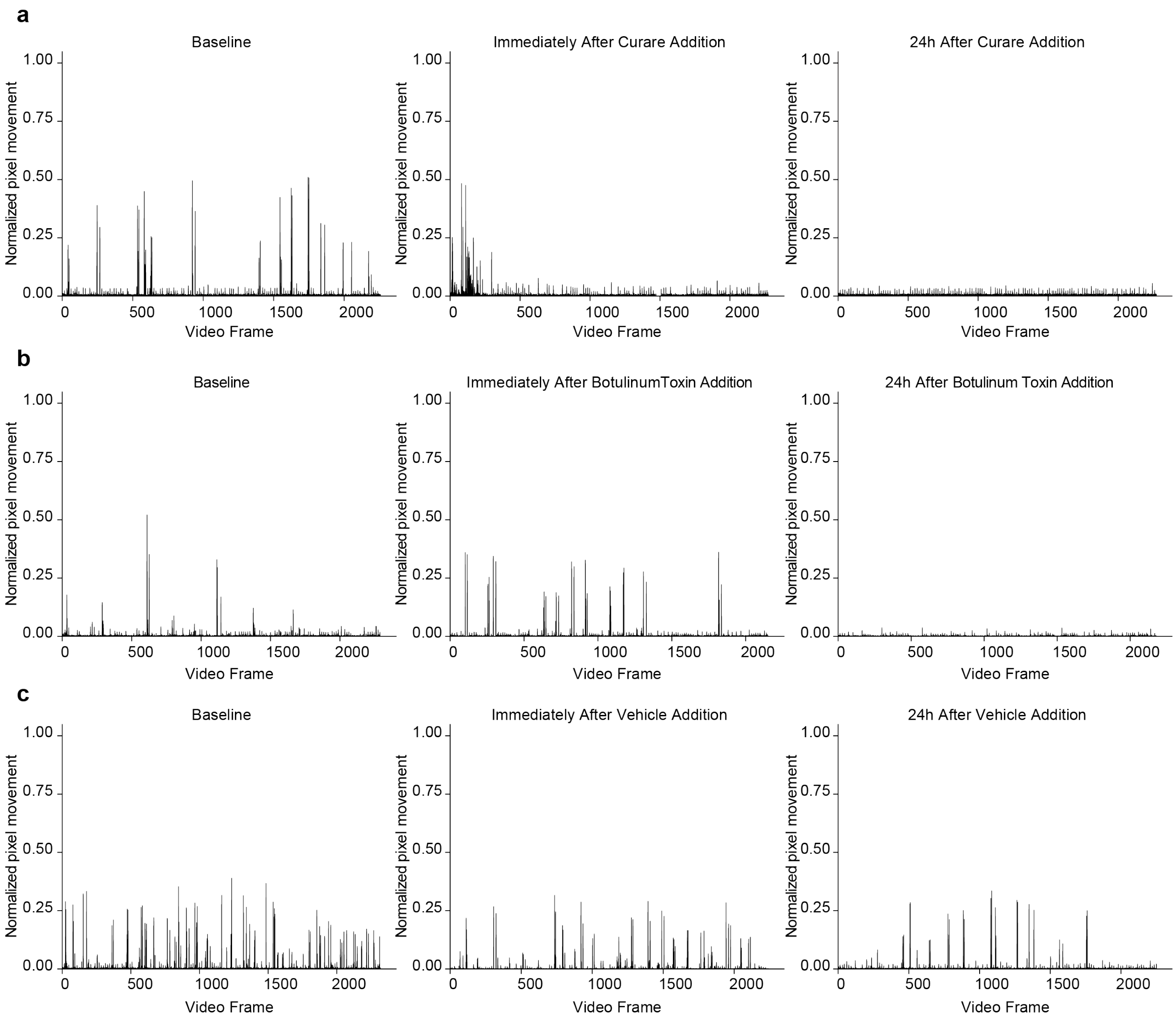

Supplementary Fig. 7 | Examples From Optic Flow Analysis of Skeletal Muscle Contractions Show Abrogation of Contractions by NMJ Blockers. **a,** Quantification of spontaneous contractions based on average normalized pixel movement, before, immediately after, and 24h after curare addition. **b,** Same time frame for botulinum toxin addition. **c,** Same time frame for vehicle addition.

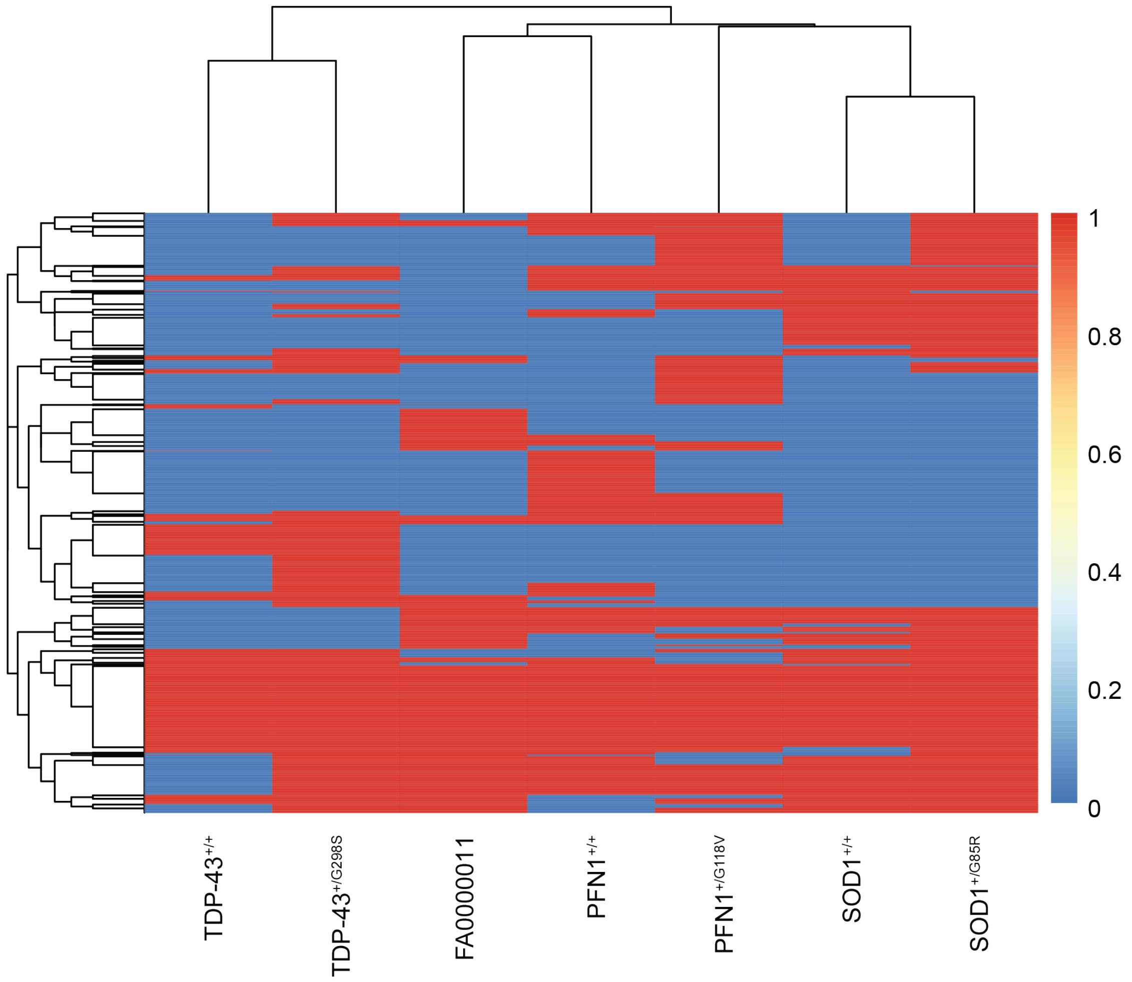

Supplementary Fig. 8 | Exome Sequencing of Isogenic iPSC lines. Clustering analysis of isogenic lines and the original control iPSC line, FA0000011.

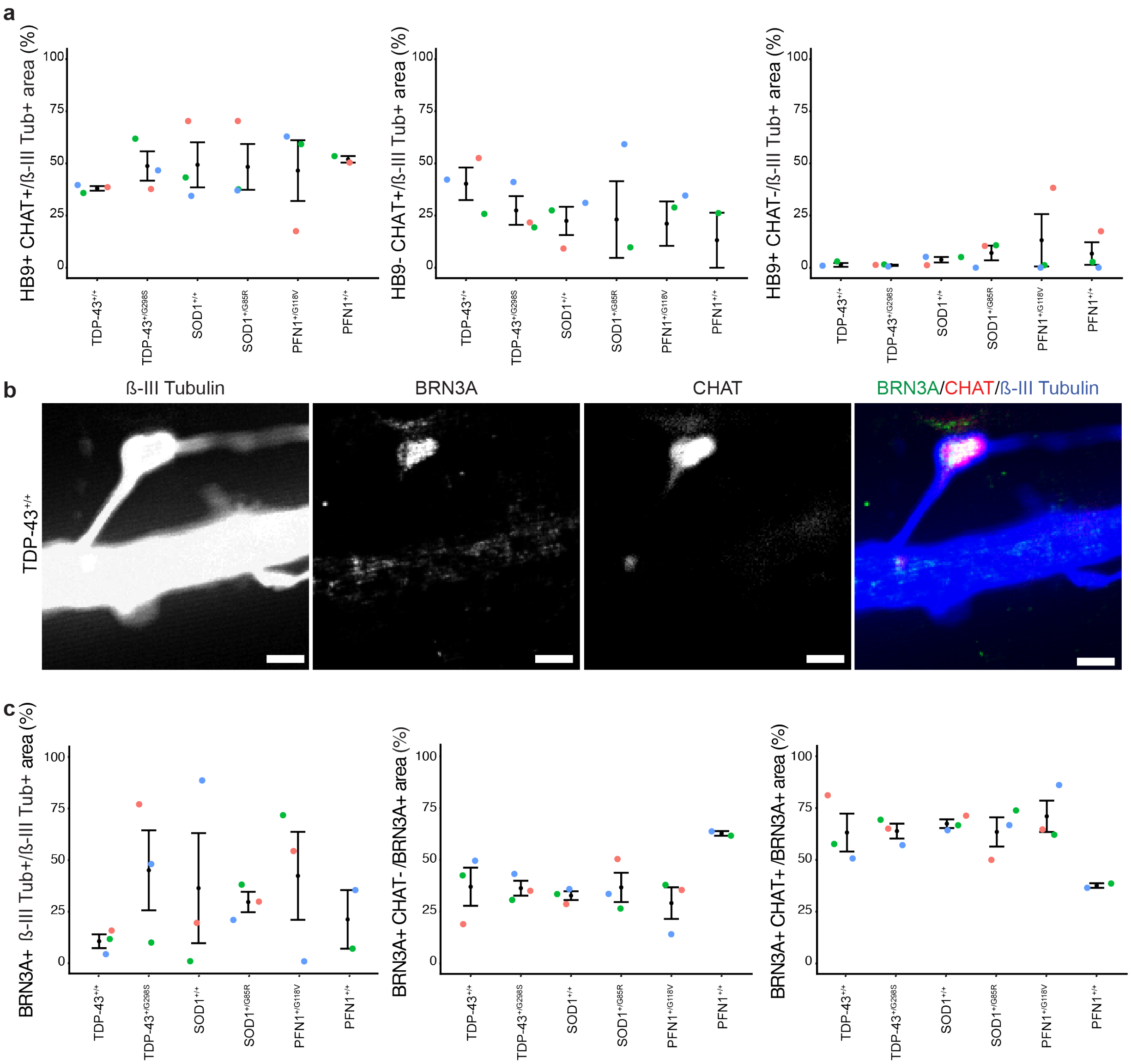

Supplementary Fig. 9 | Presence of Motor and Sensory Neurons in Whole Organoid Cultures of the Isogenic iPSC lines. a, Quantification of HB9+ ChAT+ (left), HB9- ChAT+ (middle), and HB9+ ChAT- (right) area of β-III Tubulin-expressing cells, in an unbiased grid of thirty-five fields of view per whole organoid culture in six indicated iPSC lines at four weeks (n = 3 independent biological differentiation replicates for each iPSC line). Bars indicate mean and SEM. **b,** Examples of Brn3a+, ChAT+, and β-III Tubulin+ neurons both inside and outside ganglia-like structures. Data are representative of three independent biological differentiation replicates for each indicated iPSC line. Scale bars represent 20 μm. **c,** Quantification of percentage of Brn3a+ β-III Tubulin+ area of β-III Tubulin+

neurons (left), percentage of Brn3a+ ChAT- β-III Tubulin+ neurons in the Brn3a+ β-III Tubulin+ population (middle), and percentage of Brn3a+ ChAT+ β-III Tubulin+ neurons in the Brn3a+ β-III Tubulin+ population (right), quantified in an unbiased grid of twenty fields of view per whole organoid culture in six indicated iPSC lines at four weeks (*n* = 3 independent biological differentiation replicates for each iPSC line. Bars indicate mean and SEM).

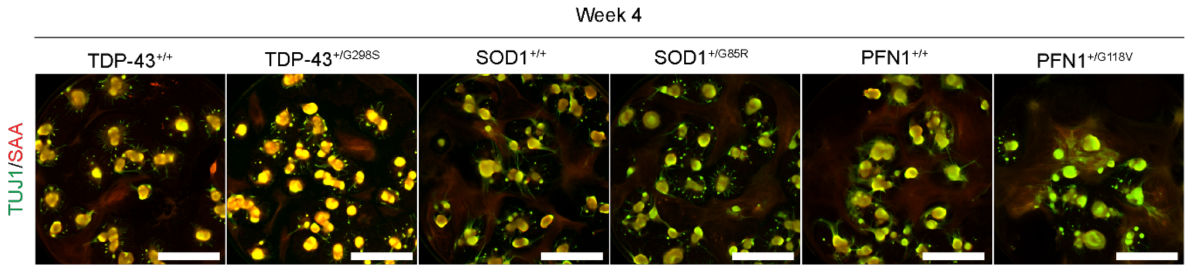

Supplementary Fig. 10 | Isogenic iPSC Lines Reduce Within- and Among-line Variability in Early Organoid Cultures. Organoid cultures of the matched isogenic pairs are qualitatively similar after four weeks in culture. Data are representative of three independent biological differentiation replicates for each indicated iPSC line. Scale bars indicate 5mm.

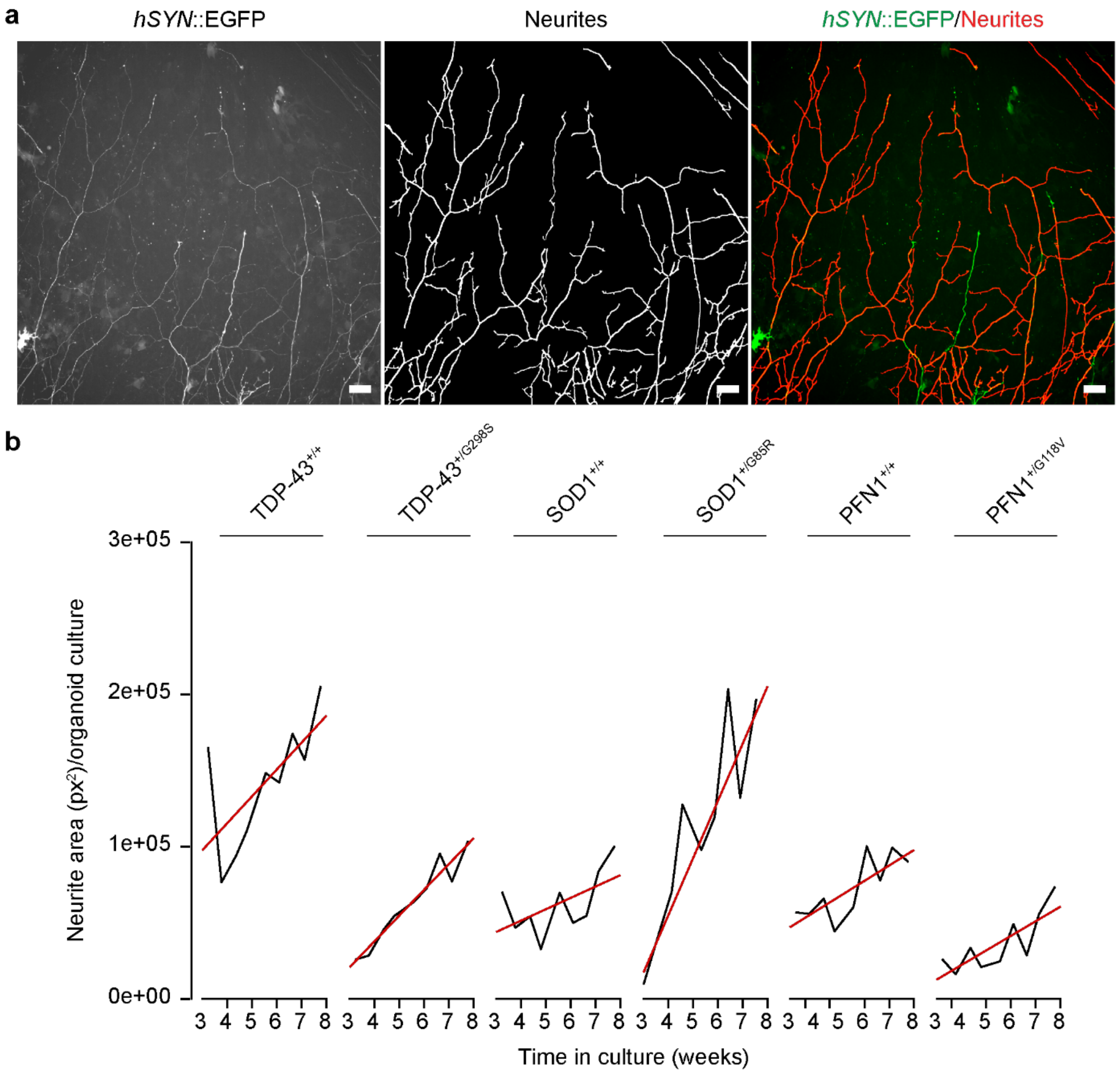

Supplementary Fig. 11 | Longitudinal neurite outgrowth analysis in live organoid cultures. a, Example of automated detection of *hSYN*::EGFP labeled neurites. Scale bars represent 25 µm. **b,** Examples of longitudinal quantification of neurite area (black), along with linear fits of outgrowth rate (red), in individual organoid cultures of each iPSC line.

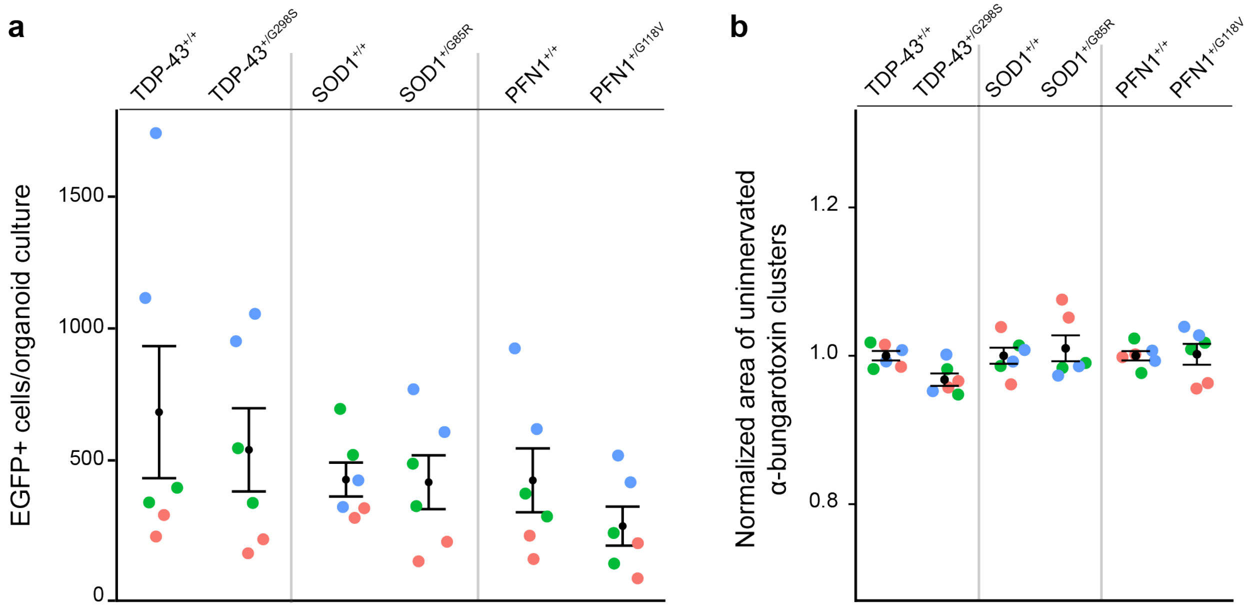

Supplementary Fig. 12| Cells expressing EGFP at 15 weeks of culture after transduction with *hSYN*::EGFP two weeks prior. a, Quantification of EGFP+ cells in organoid cultures after NMJ labeling with α-bungarotoxin and fixation. Bars indicate mean and SEM. (*n*=6 organoid cultures from three independent biological differentiation replicates identified by colors; Kruskal-Wallis: P=0.522). **b,** Normalized area of the uninnervated α-bungarotoxin at three months in culture (normalized to the paired isogenic controls). Bars indicate mean and SEM. (*n*=6 organoid cultures obtained from three independent biological differentiation replicates identified by colors; one-way ANOVA: F=0.66 P=0.656).

	Cells	Pct_total
Neural crest	5712	27.42
Intermediate progenitors	7440	35.71
Neuronal progenitors	1645	7.90
Neurons	1336	6.41
Mesenchymal progenitors	1493	7.17
Unspecified	3209	15.40

Supplementary Data Table 1 | Percentage of Major Populations Found in the Seurat Analysis of scRNA-seq.

Target Gene/Mutation	Guide RNA sequence	Predicted Off-Targets
TARDBPG298S	GGATTTGGTAATAGCAGAGG	FAM110B<br>PCDHB10<br>TTLL7<br>SLC39A11<br>NCKAP1<br>STIM1<br>PTPN14<br>BICC1<br>PEX11G<br>SULF2
SOD1G85R	ATTAGGCATGTTGGAGACTT	SOX5<br>PARM1<br>USP9X<br>USP9Y<br>TRIM71<br>SMS<br>CAPN2<br>ESYT1<br>TNPO1<br>KCNIP4
PFN1G118V	ATGGGCAAAGAAGGTGTCCA	NBPF3<br>PFN2<br>MFN1<br>LDB3<br>GPC6<br>VAV3<br>NINJ1<br>ARHGAP23<br>SGSM2<br>PLOD1

Supplementary Data Table 2 | Guide RNAs used for CRISPR/Cas9 and potential off-targets.

Antibodies		
Bassoon (1:100)	Abcam	ab110426
BRN3A (1:200)	Millipore	MAB1585
B-III Tubulin (1:500)	Abcam	ab18207
GFAP (1:200)	Sigma-Aldrich	G9269
HB9 (1:100)	DSHB	81.5C10-c
IBA1 (1:200)	Wako Pure Chemicals	019-19741
IBA1 (1:50) (Fig. 4e)	Abcam	Ab107159
ISL1/2 (1:50)	DSHB	39.4D5
PAX7 (1:500)	ThermoFisher Scientific	PA1-117
Peripherin (1:100)	Millipore	AB1530
S100β (1:200)	Abcam	ab52642
SMI32 (1:200)	BioLegend	801701
SOX2 (1:100)	Abcam	ab97959
TBXT (Brachyury) (1:200)	R&D	AF2085
TUJ1 (1:500)	BioLegend	801202
SAA (1:200)	Abcam	ab9465
β-III Tubulin (1:500)	Abcam	ab18207
APC anti-human CD56 (NCAM) Antibody (1:20)	Biolegend	362503
APC Mouse IgG1 κ Isotype Ctrl (F.C.) Antibody (1:20)	Biolegend	400121
Human Fc Receptor Binding Inhibitor Purified 100 tests antibody	Thermo Fisher Scientific	14-9161-73
ChAT (1:200)	EMD Millipore	AB144P
Isolectin B4 (1:1000)	Vectorlabs	DL-1178
α-Bungarotoxin (1:1000)	Thermo Fisher Scientific	B35450

Supplementary Data Table 3 | List of Antibodies and Dyes.

Gene	Forward (5' - 3')	Reverse (5' - 3')
CHAT (Keiger et al., 2003)	GCTCAGAACAGCAGCATCATGCCG	GCTGACGGAGTCTGCTCGGATCAG
POU4F1 (Lee et al., 2012)	CGTACCACACGATGAACAGC	AGGAGATGTGGTCCAGCAGA
PRPH (Pomp et al., 2005)	TTGAGTTCCTCAAGAAGCTGCACG	CACCTCAGGCACAGTCGTCTTTAT
TMEM119 (Satoh et al., 2016)	ACCAGCACGGACTCTCTCTTCCA	ACCAGCACGGACTCTCTCTTCCA
ISL1 (Patani et al., 2011)	CAGGTTGTACGGGATCAAATGC	CACACAGCGGAAACACTCGAT
MYOG (Xu et al., 2013)	AGATGTGTCTGTGGCCTTCC	AGCTGGCTTCCTAGCATCAG
LHX3 (Hester et al., 2011)	TGCAGGTTTGGTTCCAGAACCGCC	GCCAGGCCTCCATGCTCCAGGGAG
MYF5 (Xu et al., 2013)	GCCTGAAGAAGGTCAACCAG	CCATCAGAGCAGTTGGAGGT
CDH5 (Xu et al., 2013)	CAGCCCAAAGTGTGTGAGAA	TGTGATGTTGGCCGTGTTAT
GAPDH (Xu et al., 2013)	TGGTATCGTGGAAGGACTCA	TTCAGCTCAGGGATGACCTT

Supplementary Data Table 4 | List of qPCR Primers Used in this Study.

Supplementary Data Table 5 | Summary of Exome Sequencing Data

Gene Names	Variant Type	FA11	PFN1HC	PFN1G118V	SOD1HC	SOD1G85R	TARDBPHC	TARDBPG298S
A4GALT	frameshift_variant&stop_gained	1	0	0	0	0	0	0
AATK	frameshift_variant	1	1	0	0	0	0	0
ABCA13	downstream_gene_variant	1	1	1	1	1	1	1
ABCB11&SPC25	gene_fusion	0	1	1	0	1	0	0
ABCD1P2&RP11-291L22.4	gene_fusion	1	0	0	1	1	1	1
ABCD1P2	exon_loss_variant	0	0	0	1	1	0	0
ABCD4&VRTN	bidirectional_gene_fusion	1	1	1	0	1	1	1
ABHD13&LIG4	bidirectional_gene_fusion	0	1	1	0	0	0	1
ABHD13	sequence_feature	0	0	0	1	1	0	0
ABHD4&SLC7A7	bidirectional_gene_fusion	0	0	1	0	1	0	0
ABL1&FIBCD1	bidirectional_gene_fusion	1	1	1	0	0	1	1
AC003968.1	transcript_ablation	0	0	0	0	1	0	0
AC004070.1&AL121875.1&RP	feature_ablation	0	0	0	1	1	0	0
AC004595.1&AC005094.2&AS	feature_ablation	0	1	0	1	1	0	0
AC004899.1&AC004899.3&AC	duplication	0	0	0	0	0	1	1
AC005003.1&PATZ1	bidirectional_gene_fusion	1	1	0	1	1	0	1
AC005027.3&GLI3	gene_fusion	0	0	0	1	1	0	0
AC005324.6&TEKT3	gene_fusion	1	0	1	1	1	1	1
AC006432.1&RP11-525E9.1&	feature_ablation	1	1	1	1	1	1	1
AC006538.4&GNG7	gene_fusion	0	1	1	0	0	1	1
AC006547.14&RTN4R	gene_fusion	0	0	1	0	1	0	0
AC008174.3&FSIP2	bidirectional_gene_fusion	1	1	1	1	1	1	1
AC008498.1	transcript_ablation	0	0	0	0	0	1	1
AC009120.6&CLEC18B	gene_fusion	1	1	1	1	1	1	1
AC009365.3&PLXNA4	bidirectional_gene_fusion	0	1	0	0	0	0	0
AC009502.1&AC009502.4&RP	feature_ablation	0	0	0	0	0	1	1
AC009948.5&PRKRA	bidirectional_gene_fusion	0	1	1	0	0	0	1
AC010547.9&RP11-510M2.4	gene_fusion	0	1	0	0	0	0	0
AC010724.1&AC126339.1&AC	feature_ablation	1	1	1	1	1	1	1
AC010724.1&AC126339.1&AC	feature_ablation	0	1	1	0	0	0	0
AC010976.2&IWS1	bidirectional_gene_fusion	0	0	0	1	1	0	0
AC011484.1&PPP5C	gene_fusion	1	1	1	1	1	0	0
AC011648.1&RNU6-745P&RP	feature_ablation	0	1	0	0	0	0	0
AC011718.1&CA15P2&PPP1R	feature_ablation	0	1	0	0	0	0	0
AC011899.10&AC078942.1&	feature_ablation	1	1	1	1	1	1	1
AC012441.1&CICP17&COBL&	feature_ablation	1	1	1	1	1	1	1
AC016582.2&WDR87	gene_fusion	1	1	1	0	1	0	0
AC016626.1&OR4F17&OR4F8	duplication	1	1	1	1	1	1	1
AC016751.2&AC016751.3&AC	RNA_feature_ablation	1	1	1	1	1	1	1
AC017081.2&NDUFS1	gene_fusion	0	0	1	0	0	0	0

AC022154.7	upstream_gene_variant	1	1	1	1	1	0	1
AC023481.1&LMCD1-AS1&RN	feature_ablation	1	1	1	1	1	1	1
AC024580.1&ZNF444	duplication	0	0	0	0	0	0	1
AC025335.1&CHD3	bidirectional_gene_fusion	0	0	0	1	1	0	0
AC068020.1&ALG1L13P&ENPI	feature_ablation	0	1	0	1	1	0	0
AC068587.1&RP11-303G3.9&I	feature_ablation	0	1	0	0	0	0	0
AC069547.1&AC069547.2&AL	feature_ablation	1	0	1	1	1	0	0
AC073626.2&FOXP2&PPP1R3/	feature_ablation	0	1	0	0	0	0	0
AC073869.1&AC073869.20	gene_fusion	0	1	0	0	0	0	0
AC091304.1&GOLGA8F	duplication	1	1	1	1	1	1	1
AC092117.2&SRRM2	duplication	1	1	1	1	1	1	1
AC096579.13	splice_donor_variant&splice_region_v	0	0	0	0	0	1	0
AC102948.2&CA10&RP11-429	feature_ablation	0	0	1	0	0	0	0
AC107613.1&ARX	bidirectional_gene_fusion	1	0	1	1	1	0	0
AC108519.1&ALG1L14P&FAM	feature_ablation	0	1	1	0	0	0	0
AC109829.1&C2orf16	gene_fusion	0	0	0	0	0	1	1
AC109829.1&RP11-158I13.2	gene_fusion	1	1	1	1	1	1	1
AC113407.1&CTD-2151A2.1&(	RNA_feature_ablation	0	1	0	0	0	0	0
AC126763.1&MIR3180-4&NPI	RNA&hsa-mir-3180-4_feature_ablatio	1	1	1	1	1	1	1
AC129778.2&ANKRD20A3&AC	feature_ablation	0	0	0	0	0	1	1
AC129778.2&ANKRD20A3&AC	feature_ablation	0	1	0	0	0	0	0
AC133041.1&CTD-2026G6.2&	feature_ablation	1	1	0	1	1	1	1
AC140725.7&OR4F13P&OR4F	duplication	0	1	1	0	0	1	1
ACAN	upstream_gene_variant	1	1	1	1	1	1	1
ACER3&B3GNT6	gene_fusion	1	0	1	1	1	0	0
ACOT4&HEATR4	bidirectional_gene_fusion	0	1	1	0	0	0	1
ACOT9&SMS	bidirectional_gene_fusion	1	1	1	1	1	0	0
ACPT&CLEC11A	gene_fusion	1	1	1	1	1	1	1
ACSL3&SCG2	bidirectional_gene_fusion	0	0	1	0	1	0	0
ACTBP9&RP11-640A1.4&RPL1	feature_ablation	1	1	1	0	0	0	1
ACTL7A&KLF4	bidirectional_gene_fusion	0	1	1	0	1	0	1
ACTL9&OR2Z1&RPL23AP78	duplication	1	0	0	1	1	0	1
ACTL9	stop_gained&duplication&splice_regic	1	0	1	1	1	0	0
ACVRL1&SCN8A	gene_fusion	0	1	0	0	0	0	1
ACYP1&MLH3	gene_fusion	1	1	1	1	1	1	1
ADAL&TUBGCP4	gene_fusion	0	0	0	1	0	0	0
ADAL&ZSCAN29	bidirectional_gene_fusion	0	0	0	0	0	1	1
ADAM20&RP11-486O13.4	gene_fusion	1	0	0	0	0	0	0
ADAM21P1	non_coding_transcript_exon_variant	1	0	0	0	0	0	0
ADAM21P1	splice_region_variant&non_coding_tra	1	1	0	0	0	1	1
ADAM21	sequence_feature	1	0	0	0	0	0	0

ADAM30&NOTCH2	gene_fusion	1	1	1	1	1	0	1
ADAMTS3&NPFFR2	bidirectional_gene_fusion	0	1	0	0	0	1	1
ADAMTSL1	sequence_feature	0	0	0	0	0	0	1
ADCK2&DENND2A	bidirectional_gene_fusion	1	0	0	1	1	0	0
ADCY10	duplication	0	0	1	0	1	0	0
ADCY5&PDIA5	bidirectional_gene_fusion	0	1	0	0	0	0	1
ADD1&MFSD10	bidirectional_gene_fusion	0	0	1	0	1	0	0
ADH1A	frameshift_variant	1	1	1	0	1	0	1
ADH1B	frameshift_variant&stop_gained	1	1	0	0	0	0	0
ADH1C	stop_gained&duplication	1	1	1	1	1	0	1
ADM&AMPD3	gene_fusion	0	0	1	1	1	0	0
ADNP	duplication	0	0	0	0	0	0	1
ADRA2A	intragenic_variant	0	0	1	0	1	0	0
ADRA2B	stop_gained&duplication	0	0	0	0	0	1	1
ADRA2C&LINC00955	gene_fusion	1	0	0	1	1	0	0
ADRBK2	sequence_feature	1	1	0	1	1	0	1
AE000658.22&SALL2	bidirectional_gene_fusion	1	1	1	1	1	1	1
AF228730.2&AF228730.5&RP!	feature_ablation	0	0	1	0	0	0	0
AGAP7	duplication	1	0	1	0	1	1	1
AGAP7	frameshift_variant&stop_gained	0	0	0	0	0	1	0
AGAP9&AL603965.1&ANXA8L	feature_ablation	0	1	1	0	0	0	0
AGPS&TTC30A	bidirectional_gene_fusion	0	0	1	1	1	1	1
AGRN	sequence_feature	1	0	1	1	1	1	1
AHCTF1P1	downstream_gene_variant	1	1	1	0	0	0	1
AHCTF1	frameshift_variant&splice_donor_vari	0	1	1	1	1	0	1
AHDC1	duplication	0	0	1	0	1	0	1
AHNAK2	frameshift_variant	1	1	1	1	1	1	1
AHNAK2	frameshift_variant&splice_acceptor_v	1	1	1	1	1	1	1
AHNAK2	frameshift_variant&stop_lost	1	1	1	0	1	0	1
AHNAK2	stop_gained&duplication	0	0	0	1	1	1	1
AHNAK	duplication	0	1	1	1	1	1	1
AHNAK	frameshift_variant	0	0	1	0	1	0	0
AHNAK	frameshift_variant&stop_gained	1	0	0	0	0	0	0
AHNAK	frameshift_variant&stop_lost	0	1	1	1	1	1	1
AIM1L&ZNF683	gene_fusion	0	0	0	0	0	0	1
AIM1	frameshift_variant	1	1	1	1	1	0	1
AKAP11	sequence_feature	1	0	1	0	0	1	1
AKAP12&ZBTB2	bidirectional_gene_fusion	0	1	0	0	0	0	0
AKAP17A&ASMTL	bidirectional_gene_fusion	1	1	1	1	1	0	0
AKAP4&CLCN5	bidirectional_gene_fusion	0	0	1	0	0	1	1
AKAP5&HSPA2	gene_fusion	0	0	1	0	1	0	0

AKAP9&FZD1	gene_fusion	0	0	1	0	0	0	1
AL031666.2&ZMYND8	bidirectional_gene_fusion	0	1	0	0	0	0	0
AL161898.1&KL	duplication	0	0	1	0	1	0	0
AL162415.1&AL162415.2&AL1	RNA_feature_ablation	1	0	0	0	0	1	0
AL353791.1&FAM74A1&RN7S	feature_ablation	0	1	0	0	0	0	0
AL356019.1&AL359218.1&CTI	duplication	1	1	1	1	1	1	1
AL356019.1&AL359218.1&CTI	duplication	0	0	0	0	0	1	1
AL359195.1	exon_region	0	0	1	0	0	0	0
AL359546.1&DDX10P1&RNA5	feature_ablation	0	0	0	0	0	1	1
AL445665.1&AL445665.2&AN	feature_ablation	1	1	1	0	0	0	0
AL513478.1&ANKRD20A3&AT	feature_ablation	0	1	0	0	0	0	0
AL583784.1&CCR12P&GPR18	duplication	0	0	0	1	1	0	0
AL583842.1&AL583842.2&AL	feature_ablation	1	1	1	1	1	1	1
AL590812.1&AL590812.3&ATI	feature_ablation	0	0	1	0	0	0	0
AL627309.1&AL669831.1&AL	feature_ablation	1	0	0	1	1	1	1
AL772307.1&ANKRD20A2&AT	feature_ablation	0	0	0	0	0	1	1
AL772307.1&RP11-216M21.2	bidirectional_gene_fusion	0	1	0	0	0	0	0
ALG10B&SYT10	bidirectional_gene_fusion	0	1	0	0	0	0	0
ALG12	frameshift_variant&stop_gained	1	0	1	1	1	0	1
ALG13&ALG13-AS1	duplication	0	0	1	0	0	0	0
ALMS1-IT1	upstream_gene_variant	1	1	1	1	1	1	1
ALMS1	upstream_gene_variant	0	0	0	1	1	1	1
ALPK2	stop_gained&duplication	1	1	1	1	1	1	1
ALS2CR11	frameshift_variant&stop_gained	1	0	1	1	1	0	0
ALS2CR11	splice_acceptor_variant&duplication&	0	1	0	0	0	0	1
ALYREF&ARHGDIA	gene_fusion	1	0	0	0	0	0	0
AMBN&ENAM	gene_fusion	0	0	1	0	1	0	0
AMBRA1	frameshift_variant&splice_donor_vari	1	0	1	0	0	0	0
AMD1&CDK19	bidirectional_gene_fusion	0	0	0	0	0	0	1
AMER1&MTMR8	gene_fusion	0	1	1	0	1	1	1
AMER1	splice_acceptor_variant&splice_regior	1	1	1	1	1	1	1
AMIGO1&GPR61	bidirectional_gene_fusion	1	1	1	1	1	1	1
AMIGO3&IP6K1	gene_fusion	0	0	0	0	0	1	1
AMOTL1	sequence_feature	0	0	1	1	1	0	1
AMPH&FAM183B	gene_fusion	0	1	1	0	1	0	0
AMPH	frameshift_variant&splice_donor_vari	0	1	1	0	1	0	0
AMZ2&LRRC37A16P	bidirectional_gene_fusion	0	0	0	0	0	0	1
ANK1&KAT6A	gene_fusion	0	1	1	1	1	1	1
ANK1&RP11-930P14.1	bidirectional_gene_fusion	0	0	1	1	1	0	0
ANK3&CCDC6	gene_fusion	0	1	0	1	1	0	0
ANK3	frameshift_variant	0	0	0	0	0	1	1

ANK3	splice_donor_variant&duplication&spl	1	1	1	1	1	0	1
ANKAR&ASNSD1	gene_fusion	1	0	0	0	0	0	0
ANKAR&SLC40A1	bidirectional_gene_fusion	0	0	1	1	1	0	0
ANKHD1	sequence_feature	1	0	1	0	0	0	0
ANKRD11	frameshift_variant&splice_acceptor_v	1	1	1	1	1	1	1
ANKRD13B&RP11-68I3.2	gene_fusion	0	0	1	0	0	0	1
ANKRD17	frameshift_variant	0	0	0	0	0	1	1
ANKRD20A2&RP11-146D12.2	gene_fusion	0	1	0	0	0	0	0
ANKRD20A4	sequence_feature	0	0	1	0	0	0	0
ANKRD23&SEMA4C	gene_fusion	1	0	1	0	1	1	1
ANKRD26	frameshift_variant&splice_acceptor_v	0	0	0	0	0	0	1
ANKRD30A&ARL6IP1P2&MKN	RNA_feature_ablation	1	1	1	1	1	0	0
ANKRD31	frameshift_variant&splice_donor_vari	0	1	0	1	1	0	1
ANKRD34A	3_prime_UTR_variant	0	0	1	0	0	1	1
ANKRD36&FAHD2B	bidirectional_gene_fusion	0	0	1	0	0	0	0
ANKRD36C	frameshift_variant&start_lost	0	0	0	0	0	0	1
ANKRD36C	splice_acceptor_variant&splice_donor	0	0	0	0	0	0	1
ANKRD65&MRPL20	gene_fusion	0	1	0	0	0	1	1
ANKUB1	frameshift_variant	0	1	0	0	0	1	1
ANKUB1	frameshift_variant&splice_donor_vari	1	0	1	1	1	0	0
ANO2&GALNT8	bidirectional_gene_fusion	1	0	1	0	0	0	0
ANXA2P2&PTENP1	bidirectional_gene_fusion	1	0	0	0	0	1	1
ANXA8&GDF10	bidirectional_gene_fusion	0	1	1	0	0	0	0
AP000251.3&TIAM1	bidirectional_gene_fusion	0	0	0	0	1	0	0
AP002517.1&OR2AH1P&OR5A	duplication	0	1	0	0	0	0	0
AP1S3&SCG2	gene_fusion	0	1	1	0	0	0	0
AP3B2	frameshift_variant	1	1	1	1	1	0	0
APBB3&CD14	gene_fusion	1	0	1	1	1	1	1
APEH&MST1	bidirectional_gene_fusion	0	0	0	0	0	1	0
API5&TTC17	gene_fusion	0	0	1	0	1	0	0
APLNR	frameshift_variant&start_lost	0	1	1	0	1	0	0
APOA4	duplication	0	1	1	1	1	1	1
APOBEC4&ARPC5	gene_fusion	0	0	1	0	1	0	0
APOB	frameshift_variant	1	1	1	1	1	1	1
APOB	stop_gained&duplication	0	0	1	0	1	0	1
APOO&SAT1	bidirectional_gene_fusion	1	1	1	1	1	0	1
ARAP3&PCDH1	gene_fusion	0	0	0	0	0	1	1
AREG&AREGB	gene_fusion	0	1	1	0	0	1	1
ARHGAP20	frameshift_variant&stop_gained	0	0	1	0	0	0	0
ARHGAP21&KIAA1217	bidirectional_gene_fusion	0	0	1	0	0	0	0
ARHGAP21	frameshift_variant&splice_donor_vari	0	0	0	1	1	0	1

ARHGAP23	upstream_gene_variant	0	0	1	0	0	0	0
ARHGAP30	frameshift_variant&stop_gained	0	0	0	1	1	0	0
ARHGAP32&TP53AIP1	gene_fusion	1	1	1	1	1	1	1
ARHGEF17&P2RY2	gene_fusion	1	1	1	1	1	1	1
ARHGEF17&RP11-800A3.7	bidirectional_gene_fusion	0	0	0	0	0	0	1
ARHGEF35&OR2A1-AS1	gene_fusion	1	1	1	0	1	0	1
ARID1B	sequence_feature	0	1	0	0	0	0	0
ARID2	sequence_feature	0	1	0	0	0	0	0
ARID5A	sequence_feature	0	0	1	0	1	0	0
ARL16&HGS	bidirectional_gene_fusion	1	0	0	0	0	0	0
ARL17A	frameshift_variant&splice_region_vari	1	1	1	1	1	1	1
ARL17B&KANSL1	gene_fusion	0	0	0	1	1	0	0
ARL17B&LRRC37A	bidirectional_gene_fusion	0	0	1	0	0	0	1
ARL17B	frameshift_variant&stop_gained&splic	0	0	1	0	0	0	1
ARL4C&SH3BP4	bidirectional_gene_fusion	1	1	1	1	1	1	1
ARL4C	frameshift_variant&start_lost&splice_	0	1	1	1	1	0	0
ARMCX4&RP1-232L22	_B.1_bidirectional_gene_fusion	0	0	1	1	1	0	1
ARPC2&CXCR2	gene_fusion	0	0	0	0	0	0	1
ASH1L	sequence_feature	1	1	1	1	1	1	1
ASPM	frameshift_variant	0	0	0	1	1	0	0
ASPM	frameshift_variant&splice_acceptor_v	1	1	1	1	1	1	1
ASPM	frameshift_variant&splice_donor_vari	0	0	1	1	1	0	1
ASPM	stop_gained&duplication	0	0	0	0	0	1	1
ASXL1	downstream_gene_variant	0	1	0	0	0	0	0
ASXL2	frameshift_variant	1	1	0	0	0	0	1
ATAD2B	frameshift_variant&splice_donor_vari	0	1	1	0	0	0	0
ATAD2	stop_gained&duplication	0	1	0	0	0	0	0
ATAD3C&VWA1	gene_fusion	0	0	0	0	0	1	1
ATMIN&RP11-303E16.8	bidirectional_gene_fusion	1	0	0	0	0	0	0
ATP10A&UBE3A	gene_fusion	0	0	0	1	1	0	0
ATP5A1P1&BX664608.1&CTD-	feature_ablation	0	1	0	0	0	0	0
ATP6V1B2&GFRA2	bidirectional_gene_fusion	1	1	0	1	1	0	1
ATRN&C20orf194	bidirectional_gene_fusion	0	0	0	0	0	1	0
ATRX	frameshift_variant	1	1	1	1	1	1	1
ATXN1L&ZNF821	bidirectional_gene_fusion	1	1	1	1	1	1	1
B3GALT1&XIRP2	gene_fusion	1	1	1	1	1	1	1
B3GNT3&INSL3	bidirectional_gene_fusion	0	1	0	1	1	0	0
BAGE2	duplication	1	0	0	0	0	0	1
BAI3&EYS	bidirectional_gene_fusion	1	0	0	1	1	0	0
BAZ1B	duplication	1	1	1	1	1	0	0
BAZ1B	frameshift_variant&splice_donor_vari	0	0	0	0	0	0	1

BAZ1B	frameshift_variant&stop_gained&splic	0	1	1	1	1	0	0
BBS10	splice_acceptor_variant&duplication&	1	1	0	0	0	0	0
BCHE&SLITRK3	gene_fusion	1	0	1	0	1	0	0
BCHE	frameshift_variant&start_lost&splice_	0	0	0	0	0	0	1
BCL11A	frameshift_variant&splice_acceptor_v	1	1	0	1	1	0	1
BCL9	protein_protein_contact	1	1	1	0	0	0	0
BCORL1	sequence_feature	1	1	1	1	1	0	1
BCOR	frameshift_variant	0	1	0	0	0	0	0
BEND2&RAI2	gene_fusion	1	1	0	1	1	0	1
BEND3&C6orf203	bidirectional_gene_fusion	0	1	1	0	0	0	1
BFSP1&PCSK2	bidirectional_gene_fusion	1	0	0	0	0	0	0
BHLHB9&NXF2B	bidirectional_gene_fusion	0	0	0	0	0	1	1
BHLHE40&BHLHE40-AS1	bidirectional_gene_fusion	1	1	1	1	1	1	1
BLNK&ZNF518A	bidirectional_gene_fusion	0	1	1	0	1	0	1
BLOC1S5-TXNDC5&EEF1E1	gene_fusion	0	0	1	0	0	0	0
BMP15&RP11-363G10.2	gene_fusion	0	0	0	1	1	1	1
BMS1P1&CTGLF8P&FRMPD2P	feature_ablation	1	1	1	1	1	0	0
BMS1P16&CT60&CXADRP2&D	feature_ablation	0	0	0	1	1	1	1
BMS1P16&CXADRP2&DKFZP5	feature_ablation	0	0	1	0	1	0	0
BMS1P20&PRAME	bidirectional_gene_fusion	0	0	0	1	1	0	0
BMS1P8&ENPP7P13&IGHV3O	duplication	1	1	0	1	1	1	1
BNC2	frameshift_variant&splice_acceptor_v	1	1	1	0	0	0	1
BNC2	frameshift_variant&stop_gained	0	1	1	0	0	0	0
BOD1L1	frameshift_variant&splice_acceptor_v	1	1	1	1	1	1	1
BOLA1&HIST2H2BC	bidirectional_gene_fusion	1	1	0	1	1	0	1
BOLA1&HIST2H2BF	bidirectional_gene_fusion	0	0	0	0	0	1	1
BRCA1	frameshift_variant&stop_gained&splic	1	1	1	1	1	1	1
BRCA2	downstream_gene_variant	1	1	1	1	1	0	1
BRD1&ZBED4	bidirectional_gene_fusion	1	1	1	1	1	0	1
BRINP1&TLR4	bidirectional_gene_fusion	1	1	0	1	1	0	1
BRIP1&TBX4	bidirectional_gene_fusion	1	1	1	1	1	1	1
BRSK2&MOB2	bidirectional_gene_fusion	1	0	0	1	1	1	1
BRWD1&PSMG1	gene_fusion	0	0	1	0	0	0	1
BRWD1	frameshift_variant	1	1	0	1	1	1	1
BRWD3&TBX22	bidirectional_gene_fusion	0	1	0	1	1	1	1
BSN	sequence_feature	1	1	1	1	1	1	1
BTAF1	sequence_feature	0	0	0	1	1	0	0
BTBD18	frameshift_variant&stop_gained&splic	1	0	0	0	0	0	0
BTBD3&SPTLC3	gene_fusion	0	0	0	1	1	0	0
C10orf118&NHLRC2	bidirectional_gene_fusion	1	1	1	0	0	0	0
C10orf118	splice_acceptor_variant&splice_regior	0	1	0	0	0	0	0

C10orf120&DMBT1	bidirectional_gene_fusion	0	0	1	1	1	0	1
C10orf120	frameshift_variant	0	1	0	0	0	0	0
C10orf71	sequence_feature	1	1	1	1	1	0	1
C11orf80&LRFN4	gene_fusion	0	1	1	1	1	0	1
C11orf82	sequence_feature	1	0	0	0	0	0	0
C16orf87&MYLK3	gene_fusion	1	1	1	0	1	0	1
C16orf91	frameshift_variant&stop_gained	0	0	0	0	0	1	1
C17orf47	duplication	0	0	0	1	1	0	0
C17orf82&TBX2	gene_fusion	1	1	1	1	1	1	1
C1ORF220&C1orf220&MIR442	feature_ablation	1	0	1	1	1	0	1
C1QTNF2&C5orf54	gene_fusion	1	1	1	1	1	0	1
C1QTNF2&SLU7	gene_fusion	0	1	1	0	0	0	0
C1orf100&ZBTB18	gene_fusion	0	0	1	0	0	0	0
C1orf106&CAMSAP2	gene_fusion	0	0	0	1	1	0	0
C1orf173&RP4-612J11.1	bidirectional_gene_fusion	0	1	1	1	1	1	0
C1orf173	frameshift_variant&splice_acceptor_v	0	0	0	0	0	0	1
C1orf173	frameshift_variant&stop_gained&splic	1	1	1	1	1	1	1
C1orf222&GABRD	bidirectional_gene_fusion	0	1	1	0	0	0	0
C1orf52&SYDE2	gene_fusion	1	1	0	1	1	0	0
C1orf56	sequence_feature	0	1	0	0	0	0	0
C1orf68&KPRP&RP1-43O17.1	duplication	1	1	1	1	1	1	1
C2orf27A&C2orf27B	bidirectional_gene_fusion	1	1	1	1	1	1	1
C2orf44	splice_acceptor_variant&splice_donor	0	1	0	0	0	0	0
C2orf71	splice_donor_variant&duplication&spl	1	1	1	1	1	1	1
C2orf81	frameshift_variant	0	1	1	0	0	0	0
C2orf88&MSTN	bidirectional_gene_fusion	1	0	0	0	0	0	0
C4orf19&KIAA1239	gene_fusion	0	0	0	0	0	1	1
C4orf21	frameshift_variant	1	1	1	1	1	1	1
C4orf21	frameshift_variant&splice_acceptor_v	0	1	1	0	0	1	1
C4orf40&CSN1S2AP	gene_fusion	0	0	0	0	0	1	1
C4orf50	duplication	0	0	1	1	1	0	0
C5AR1&DHX34	gene_fusion	0	1	1	0	1	0	0
C5orf60&CBY3	gene_fusion	1	1	1	1	1	0	1
C6orf10&XXbac-BPG154L12.4	bidirectional_gene_fusion	0	0	0	1	1	0	1
C6orf10	frameshift_variant	1	1	1	0	1	1	1
C6orf47&GPANK1	gene_fusion	1	0	1	1	1	0	0
C9orf131&DNAJB5	gene_fusion	1	1	1	1	1	1	1
C9orf171&TTF1	bidirectional_gene_fusion	0	1	1	0	0	0	0
C9orf72	duplication	1	0	0	0	0	0	0
CA7&PDP2	gene_fusion	0	0	0	1	1	1	1
CACNG8&SIGLEC5	bidirectional_gene_fusion	1	1	1	1	1	0	0

CACNG8&ZNF611	bidirectional_gene_fusion	0	1	0	0	0	0	0
CADPS2&RNF148	gene_fusion	0	1	1	1	1	0	0
CAGE1	frameshift_variant	0	1	0	0	0	0	0
CAHM&PACRG&QKI&RP3-495	feature_ablation	0	0	1	1	1	0	0
CALHM2&CALHM3	gene_fusion	1	1	1	1	1	1	1
CAMK2D&UGT8	bidirectional_gene_fusion	0	0	0	0	0	1	1
CAMSAP1	frameshift_variant	1	1	0	1	1	0	1
CAMSAP1	stop_gained&duplication	0	0	0	0	0	1	1
CAMTA1	sequence_feature	1	1	1	0	1	0	0
CAND1	sequence_feature	0	1	1	0	0	0	0
CASC16&RP11-297L17.2&RP1	feature_ablation	0	1	1	0	0	0	0
CASC5&RPUSD2	gene_fusion	0	0	1	0	1	0	0
CASC5	protein_protein_contact	1	0	0	0	0	0	0
CASC5	sequence_feature	0	0	1	1	1	1	1
CASK&MAOB	gene_fusion	0	0	1	0	1	0	0
CASP8AP2&MAP3K7	bidirectional_gene_fusion	1	1	1	1	1	0	0
CASR	splice_region_variant&intron_variant	1	1	1	1	1	0	1
CAV3&OXTR	bidirectional_gene_fusion	0	1	0	0	0	0	0
CBY3&HNRNPH1	gene_fusion	0	0	0	0	0	1	1
CCAR2&RP11-582J16.5	duplication	1	1	0	1	1	0	1
CCDC106&ZNF524	gene_fusion	0	0	0	0	0	0	1
CCDC110	frameshift_variant	0	0	1	1	1	0	0
CCDC110	frameshift_variant&stop_lost	0	1	1	0	1	0	1
CCDC112	frameshift_variant	0	0	0	1	1	0	0
CCDC127&PLEKHG4B	bidirectional_gene_fusion	1	0	0	0	0	0	1
CCDC144B&FBXW10	bidirectional_gene_fusion	1	1	1	1	1	1	1
CCDC146&RP11-467H10.2	gene_fusion	1	0	0	0	0	0	0
CCDC147&ITPRIP	bidirectional_gene_fusion	0	0	1	0	0	0	0
CCDC168&LINC00283	duplication	1	1	1	1	1	1	1
CCDC172	exon_loss_variant	0	0	0	0	0	1	1
CCDC177	duplication	0	1	1	0	0	0	0
CCDC17	frameshift_variant&start_lost	1	1	1	1	1	0	1
CCDC73	frameshift_variant&splice_acceptor_v	0	1	0	1	1	0	1
CCDC80	stop_gained&duplication	0	0	0	1	1	0	0
CCDC82&JRKL	bidirectional_gene_fusion	0	1	0	0	0	0	0
CCDC87&CCS	bidirectional_gene_fusion	0	1	1	0	0	0	0
CCDC88C	splice_acceptor_variant&splice_donor	1	1	0	0	1	0	0
CCIN&GLIPR2	gene_fusion	0	1	0	0	0	0	0
CCNB3&CLCN5	gene_fusion	0	1	1	1	1	0	0
CCNL1	frameshift_variant	0	0	0	0	0	1	1
CCNT1&KANSL2	gene_fusion	0	1	1	0	1	1	1

CCPG1	frameshift_variant&splice_donor_vari	0	1	0	0	0	0	0
CCR2&CCR3	gene_fusion	1	0	0	0	0	0	0
CCSER1&MMRN1	gene_fusion	1	1	0	1	1	0	1
CCT8L2	frameshift_variant&stop_gained	0	0	1	1	1	0	0
CD177P1&CEACAM1	gene_fusion	1	1	1	1	1	0	1
CD177P1&PSG1	gene_fusion	0	0	1	0	0	0	0
CD177P1&PSG3	gene_fusion	0	0	0	0	0	1	1
CD180&MAST4	bidirectional_gene_fusion	1	1	1	1	1	1	1
CD40	3_prime_UTR_variant	0	1	1	1	1	0	0
CD53&RP11-96K19.2	gene_fusion	0	1	1	1	1	0	0
CD5L	frameshift_variant	1	1	1	1	1	1	1
CDC20	structural_interaction_variant	0	1	0	0	0	0	0
CDC37P1&RP11-57A19.4	gene_fusion	0	0	0	0	0	0	1
CDK16&USP11	gene_fusion	0	1	0	0	0	0	0
CDK1	protein_protein_contact	0	0	0	1	1	0	1
CDK1	structural_interaction_variant	0	0	1	0	1	0	0
CDK20	splice_region_variant&intron_variant	0	0	1	0	0	0	0
CDK6&FAM133B	gene_fusion	0	0	0	0	0	1	1
CDKAL1&RP1-135L22.1&RP11	feature_ablation	0	0	1	1	1	1	1
CDX4&DMRTC1	bidirectional_gene_fusion	1	1	1	1	1	1	1
CEBPA&SLC7A10	gene_fusion	0	1	1	1	1	0	1
CEBPZ&NDUFAF7	bidirectional_gene_fusion	1	0	0	1	1	0	0
CELSR1	frameshift_variant	1	1	1	0	1	0	0
CELSR1	frameshift_variant&splice_donor_vari	0	0	0	1	1	0	1
CELSR2	sequence_feature	1	1	1	1	1	1	1
CELSR3	sequence_feature	1	1	1	1	1	0	1
CENPJ	frameshift_variant	1	1	1	1	1	0	1
CENPW&MIR588&PRELID1P18	feature_ablation	1	0	0	0	0	0	0
CEP170&RP11-261C10.4	bidirectional_gene_fusion	1	0	1	1	1	1	1
CEP170P1	intragenic_variant	0	1	0	0	0	0	0
CEP170	frameshift_variant	0	0	1	0	1	0	0
CEP97	sequence_feature	0	0	1	0	0	0	0
CETN2&MAGEA3	gene_fusion	0	1	1	0	0	0	0
CH17-132F21.1	exon_region	1	0	1	1	1	0	1
CHAMP1	sequence_feature	1	1	1	1	1	1	1
CHD1&RGMB	bidirectional_gene_fusion	0	1	0	0	0	0	0
CHD3&KCNAB3	bidirectional_gene_fusion	0	0	1	0	0	0	0
CHD9&RP11-467J12.4	bidirectional_gene_fusion	0	1	0	0	0	0	0
CHML	frameshift_variant&stop_gained	0	1	1	1	1	0	0
CHPF2&SMARCD3	bidirectional_gene_fusion	0	0	1	0	1	0	0
CHRFAM7A	frameshift_variant	0	0	1	0	1	0	0

CHRM4&MDK	duplication	0	1	0	0	0	0	0
CHRM5	sequence_feature	0	0	1	0	1	0	0
CHST12	sequence_feature	1	1	0	0	1	1	1
CHST2	sequence_feature	0	0	0	0	0	0	1
CHST3&SPOCK2	bidirectional_gene_fusion	0	0	0	0	0	1	1
CHST4	sequence_feature	1	1	1	1	1	0	1
CHST8&KCTD15	gene_fusion	0	0	0	0	0	0	1
CHSY1	duplication	1	0	0	0	0	0	0
CICP18&LINC00266-3&OR4F1	feature_ablation	0	1	0	0	0	0	0
CIC	downstream_gene_variant	0	1	1	0	0	0	0
CIITA	downstream_gene_variant	1	1	1	1	1	0	1
CIITA	sequence_feature	0	0	1	0	1	0	0
CILP2&PBX4	bidirectional_gene_fusion	1	0	0	0	0	0	0
CILP	frameshift_variant&splice_acceptor_v	0	1	1	1	1	1	1
CIR1&OLA1	gene_fusion	1	0	0	1	1	0	0
CIR1&SP9	bidirectional_gene_fusion	0	1	1	0	1	0	0
CKAP2L	frameshift_variant	1	1	1	0	1	1	1
CKAP2L	splice_acceptor_variant&duplication&	0	1	1	0	0	0	0
CKMT1A	upstream_gene_variant	0	0	1	0	1	0	0
CLASP2	frameshift_variant&splice_acceptor_v	0	0	1	0	0	0	0
CLDN11&SLC7A14	bidirectional_gene_fusion	0	0	1	0	1	0	0
CLEC18B	frameshift_variant	1	1	1	1	1	1	1
CLK4&ZNF354A	gene_fusion	0	0	1	0	0	0	0
CLN8&ERICH1-AS1	gene_fusion	1	1	1	1	1	0	1
CLUH	frameshift_variant&splice_acceptor_v	1	0	1	0	0	0	0
CMIP	sequence_feature	0	0	0	0	0	0	1
CMTR2	sequence_feature	0	1	0	0	0	0	1
CNDP1&ZNF407	gene_fusion	0	1	1	0	0	0	0
CNIH3&WDR26	bidirectional_gene_fusion	1	0	0	0	0	0	0
CNKSR2&KLHL34	bidirectional_gene_fusion	0	0	1	0	1	0	0
CNTN5&MTMR2	bidirectional_gene_fusion	0	0	1	1	1	0	1
CNTNAP3&FAM201A&RNU6-7	feature_ablation	0	1	0	0	0	0	0
COBLL1	stop_gained&duplication	1	0	0	0	1	0	0
COBL	frameshift_variant&splice_acceptor_v	0	1	1	1	1	1	1
COBL	stop_gained&duplication	1	1	1	1	1	1	1
COG5&GPR22	bidirectional_gene_fusion	0	0	1	0	1	0	1
COL18A1	sequence_feature	1	1	1	1	1	1	1
COL24A1&ZNHIT6	gene_fusion	1	0	0	0	0	0	0
COL24A1	frameshift_variant	1	1	1	0	1	1	1
COL27A1	sequence_feature	1	1	1	0	0	0	1
COL28A1&MIOS	bidirectional_gene_fusion	0	0	1	0	1	0	0

COL6A1&COL6A2	gene_fusion	1	1	1	0	1	1	1
COMMD10&CTB-118N6.3	gene_fusion	1	0	0	1	1	0	0
COX11	frameshift_variant	0	0	0	0	0	0	1
CPD	sequence_feature	1	0	1	0	0	0	1
CPNE1&RBM12	gene_fusion	0	1	0	1	1	1	1
CPSF3L&DVL1	gene_fusion	1	1	1	1	1	1	1
CPSF3L	frameshift_variant	0	0	0	1	1	0	0
CPSF3L	frameshift_variant&start_lost	0	0	0	1	1	0	0
CREBZF&SYTL2	gene_fusion	0	0	1	0	1	0	0
CROCC&MST1P2	gene_fusion	0	0	0	0	0	1	0
CROCCP2	upstream_gene_variant	1	0	0	0	0	1	1
CROCC	splice_donor_variant&splice_region_v	0	0	0	1	1	0	0
CROT	sequence_feature	1	1	1	1	1	0	0
CSAG2&MAGEA2	bidirectional_gene_fusion	1	1	1	0	0	0	0
CSRNP3&SCN2A	gene_fusion	0	0	0	0	0	1	1
CS	frameshift_variant&splice_donor_vari	0	0	1	0	0	0	0
CT45A1&CT45A2	gene_fusion	1	0	1	1	1	0	0
CT45A1	intron_variant	0	1	0	0	0	0	0
CTAGE11P&TBC1D4	gene_fusion	0	1	1	1	1	0	0
CTAGE15&CTAGE15	gene_fusion	1	1	1	1	1	0	1
CTAGE4	sequence_feature	1	1	1	0	1	0	1
CTAGE6	frameshift_variant&stop_gained	1	1	1	1	1	1	1
CTAGE9&OR2A4	gene_fusion	1	1	1	0	0	1	1
CTB-31N19.2&CTD-2547E10.2	gene_fusion	0	0	0	0	0	0	1
CTB-59C6.3&ZNF574	bidirectional_gene_fusion	1	1	1	1	1	0	1
CTC-457E21.3&ZNF99	bidirectional_gene_fusion	1	1	0	1	1	0	0
CTC-479C5.10&EDC4	gene_fusion	1	1	0	1	1	0	0
CTC-479C5.10&LCAT	bidirectional_gene_fusion	1	0	0	0	0	0	0
CTC-513N18.7&ZNF737	gene_fusion	0	0	0	0	0	1	1
CTD-2005H7.1&CTD-2005H7.2	feature_ablation	1	1	1	0	1	0	1
CTD-2173L22.4	duplication	0	1	0	0	0	0	0
CTD-2186M15.3	transcript_ablation	1	0	1	1	1	1	1
CTD-2192J16.20&ZNF44	gene_fusion	0	0	0	1	1	0	1
CTD-2224J9.8&ZNF845	gene_fusion	0	0	1	1	1	1	1
CTD-2313N18.5	splice_donor_variant&splice_region_v	1	0	0	1	1	0	1
CTD-2370N5.3&NF1	bidirectional_gene_fusion	0	1	1	1	1	0	1
CTD-2410N18.5	splice_donor_variant&splice_region_v	0	0	1	0	1	0	0
CTD-2540B15.13&LRP3	duplication	0	1	0	1	1	0	1
CTD-2547E10.2	splice_donor_variant&splice_region_v	1	1	1	1	1	1	1
CTD-2554C21.3	upstream_gene_variant	0	1	1	0	0	0	0
CTD-2571L23.8&EHD2	gene_fusion	1	1	0	0	1	0	1

CTD-2571L23.8&ZNF541	bidirectional_gene_fusion	0	1	0	0	1	0	0
CTD-3065J16.9&EXOSC4&OPL	duplication	1	0	0	0	0	0	0
CTD-3149D2.4&MAP1S	bidirectional_gene_fusion	1	1	1	1	1	0	0
CTGLF10P	exon_region	0	1	1	0	1	0	0
CTGLF11P	downstream_gene_variant	0	0	1	0	1	0	1
CTGLF8P&PTPN20A	gene_fusion	0	0	0	0	0	1	1
CTH	structural_interaction_variant	1	0	0	0	0	0	0
CTNND1&OR10Q1	bidirectional_gene_fusion	1	0	0	0	0	0	0
CTTN&SHANK2	bidirectional_gene_fusion	0	0	1	0	1	0	0
CTTNBP2&NAA38	bidirectional_gene_fusion	0	0	0	0	0	0	1
CWC22	frameshift_variant&stop_gained	0	1	1	1	1	0	0
CXCR1&CXCR2	bidirectional_gene_fusion	1	1	0	0	0	0	0
CXorf21&NR0B1	gene_fusion	0	0	1	0	0	0	0
CXorf23&MAP7D2	gene_fusion	1	0	0	1	1	0	1
CXorf23	duplication	0	1	0	0	0	0	0
CXorf31&RP2	bidirectional_gene_fusion	0	0	1	0	0	0	1
CXorf31&SLC9A7	gene_fusion	0	0	1	0	1	0	0
CXorf40A&TMEM185A	bidirectional_gene_fusion	0	0	1	1	1	1	1
CXorf40B&DUTP4&HSFX1&MA	duplication	0	0	1	1	1	0	0
CXorf49&CXorf49B	bidirectional_gene_fusion	1	0	0	0	1	0	0
CXorf66	sequence_feature	0	0	0	0	1	0	0
CXorf66	stop_gained&duplication	1	0	0	0	0	1	1
CYB5D1&DNAH2	gene_fusion	0	1	1	1	1	0	0
CYP4F24P&ZNF861P	gene_fusion	0	0	0	1	1	0	1
CYP4V2	stop_gained&duplication	0	0	1	0	1	0	0
DBIL5P&FAM57A	gene_fusion	0	0	0	1	1	0	0
DBP&SPHK2	bidirectional_gene_fusion	1	1	1	1	1	0	1
DBX1&HTATIP2	bidirectional_gene_fusion	0	1	1	0	0	0	0
DCAF4L1	sequence_feature	0	0	1	0	1	0	0
DCAF5	frameshift_variant&stop_gained	1	0	0	1	1	1	1
DCAF5	frameshift_variant&stop_lost	0	1	1	0	0	0	0
DCDC1	splice_acceptor_variant&splice_donor	0	0	0	1	0	0	0
DCHS2&SFRP2	gene_fusion	0	1	1	1	1	0	1
DCHS2	frameshift_variant&splice_donor_vari	0	1	1	1	1	0	1
DCLK2&NR3C2	bidirectional_gene_fusion	0	0	1	0	1	0	0
DCLK3	frameshift_variant&splice_donor_vari	1	1	1	0	0	1	1
DCLRE1A	frameshift_variant	1	1	1	0	0	0	1
DCUN1D4&SGCB	bidirectional_gene_fusion	0	0	1	0	1	0	0
DCX&TRPC5	gene_fusion	0	0	0	1	1	0	0
DDX11	sequence_feature	1	1	1	1	1	1	1
DDX12P&RP11-22B23.1	bidirectional_gene_fusion	0	0	1	1	1	0	0

DDX19A&RP11-529K1.3	gene_fusion	1	1	1	1	1	0	1
DDX19B	protein_protein_contact	0	1	0	0	0	0	0
DDX20	structural_interaction_variant	0	1	1	1	1	0	1
DDX3X&NYX	gene_fusion	0	1	1	0	1	0	1
DDX4	sequence_feature	0	0	0	0	0	0	1
DDX5&POLG2	gene_fusion	0	0	1	0	0	0	0
DDX53&PTCHD1	gene_fusion	0	1	0	0	0	0	0
DEC1&LINC00474&PAPPA&RP	feature_ablation	1	0	0	0	0	0	0
DEDD2&ZNF526	bidirectional_gene_fusion	1	1	1	1	1	0	1
DEGS1	sequence_feature	0	0	1	1	1	0	1
DENND2C	sequence_feature	0	0	0	1	1	0	0
DERL3	frameshift_variant	0	1	1	0	1	0	0
DFNB31	frameshift_variant&start_lost	1	0	0	0	0	0	0
DHX30&MIR1226	duplication	0	1	0	0	0	1	1
DIAPH3&PCDH17	bidirectional_gene_fusion	1	1	1	1	1	1	1
DIDO1	duplication	1	0	1	0	0	0	0
DIDO1	frameshift_variant&stop_lost	1	0	1	1	1	1	1
DIO3	rare_amino_acid_variant	0	0	1	0	0	0	1
DIRAS1&GNG7	gene_fusion	0	0	0	1	1	0	0
DISP1&TLR5	bidirectional_gene_fusion	0	0	0	0	0	1	1
DISP1	sequence_feature	0	0	1	0	0	0	0
DKFZP761J1410&EPOR	bidirectional_gene_fusion	0	0	0	1	1	0	1
DLC1&RP11-145O15.3	gene_fusion	0	1	0	0	0	0	1
DLC1	frameshift_variant&splice_donor_vari	0	1	0	0	0	0	1
DLL3&EID2	bidirectional_gene_fusion	0	1	1	0	1	0	1
DLL4&VPS18	gene_fusion	0	0	0	0	1	0	0
DLX1&METAP1D	gene_fusion	1	0	0	1	1	1	1
DMBT1	splice_acceptor_variant&splice_regior	0	1	1	0	0	0	0
DMD&FAM47A	gene_fusion	0	0	0	0	0	1	1
DNAAF2	start_lost&splice_donor_variant&dupl	0	0	0	0	0	1	1
DNAH3	frameshift_variant	0	0	1	1	1	0	0
DNAH7	frameshift_variant	1	0	0	1	1	0	0
DNAJB7	stop_gained&duplication	1	0	0	0	0	0	1
DNAJC13	upstream_gene_variant	0	0	1	0	1	0	0
DNAJC22	sequence_feature	1	0	0	0	0	0	0
DNAJC28	duplication	0	0	0	1	0	0	0
DNAJC28	sequence_feature	1	0	0	1	1	0	0
DNM1P47	intragenic_variant	1	1	1	1	1	1	1
DNMBP	splice_donor_variant&duplication&spl	1	1	1	1	1	1	1
DNTTIP2	duplication	1	1	1	0	1	0	1
DOC2A	frameshift_variant&stop_gained	0	0	0	0	0	1	1

DOCK1&FAM196A	bidirectional_gene_fusion	0	0	1	0	1	0	0
DOPEY1	sequence_feature	1	1	1	0	0	0	0
DOT1L&PLEKHJ1	bidirectional_gene_fusion	0	0	1	0	0	0	0
DPYSL2&RP11-613H2.1	bidirectional_gene_fusion	0	0	0	0	0	0	1
DRD5&SLC2A9	bidirectional_gene_fusion	1	1	1	1	1	1	1
DSE&TSPYL4	bidirectional_gene_fusion	0	1	0	0	0	0	0
DSEL&TMX3	gene_fusion	1	1	1	1	1	1	1
DST	frameshift_variant	1	1	1	1	1	1	1
DST	frameshift_variant&splice_donor_vari	0	1	0	0	0	0	0
DST	stop_gained&duplication	1	1	1	1	1	0	0
DTX4&OR4D10	gene_fusion	0	0	0	0	1	0	0
DTX4&OR4D9	gene_fusion	1	0	0	0	0	1	1
DUSP22&RP3-416J7.5	gene_fusion	0	0	0	0	0	1	1
DVL1&GLTPD1	bidirectional_gene_fusion	0	0	0	1	1	0	0
DYNLL2&SRSF1	bidirectional_gene_fusion	0	1	0	0	0	0	0
DYRK1A&KCNJ6	bidirectional_gene_fusion	0	1	1	1	1	0	0
DYRK4&RAD51AP1	gene_fusion	0	0	1	1	1	0	0
EBNA1BP2&WDR65	bidirectional_gene_fusion	0	1	0	0	0	0	0
ECT2L&RP3-509I19.1	gene_fusion	0	1	0	0	0	0	0
EDC4&LCAT	bidirectional_gene_fusion	0	1	1	1	1	0	1
EDEM3&FAM129A	gene_fusion	0	1	0	1	1	1	1
EDEM3	frameshift_variant&splice_donor_vari	0	0	1	0	0	0	0
EEF1E1-BLOC1S5&TXNDC5	gene_fusion	0	0	1	0	0	0	0
EEF2&ZBTB7A	gene_fusion	0	1	1	1	0	0	0
EFNB1&PJA1	bidirectional_gene_fusion	0	1	0	1	1	1	1
EHD3	sequence_feature	1	1	1	0	1	1	1
EIF3B&SNX8	bidirectional_gene_fusion	0	0	1	0	1	0	0
EIF4B&KRT18	gene_fusion	0	1	0	0	0	0	0
EIF4EBP2P1&PPIAP21&PTPRT8	RNA_feature_ablation	1	0	0	0	0	0	0
EIF5A	structural_interaction_variant	0	1	0	0	0	0	1
ELF1	duplication	0	0	0	1	1	0	0
ELF1	frameshift_variant	0	1	0	0	0	0	0
ELFN1	sequence_feature	1	1	1	0	1	0	1
ELFN2	frameshift_variant	0	0	1	0	1	1	1
EMC2&NUDCD1	bidirectional_gene_fusion	0	1	0	0	0	0	0
EMC2&TRHR	gene_fusion	0	0	1	1	1	0	1
EMD&FLNA	bidirectional_gene_fusion	0	0	0	1	1	0	0
EMX2OS&PDZD8	gene_fusion	0	0	1	0	0	0	0
ENC1	splice_acceptor_variant&splice_regior	0	0	0	0	0	0	1
ENDOD1&KDM4D	gene_fusion	0	1	1	0	0	0	1
EP300&L3MBTL2	gene_fusion	1	0	0	0	0	0	1

EPB41L2	splice_acceptor_variant&splice_donor	1	0	1	0	0	0	0
EPHA1-AS1&TAS2R41	gene_fusion	1	0	0	0	0	0	0
EPM2AIP1	duplication	0	0	1	0	0	0	0
EPM2AIP1	frameshift_variant&stop_gained	0	0	1	0	0	0	0
EPM2AIP1	stop_gained&duplication	0	1	1	0	1	0	0
EPPK1&PLEC	gene_fusion	0	1	0	1	1	0	1
ERCC6&SLC18A3	bidirectional_gene_fusion	0	1	1	0	1	0	0
ERCC6L	duplication	1	0	0	0	1	0	0
ERF	frameshift_variant&stop_gained	0	1	0	0	0	0	0
ERI1&SGK223	bidirectional_gene_fusion	1	0	0	0	0	1	1
ESCO1	splice_donor_variant&duplication&spl	1	1	1	1	1	1	1
ESPNP&MST1L	gene_fusion	1	1	1	0	0	0	0
ESPNP&MST1P2	bidirectional_gene_fusion	1	1	1	0	0	1	1
ESYT1	sequence_feature	0	0	1	0	1	0	0
EVI2B&OMG	gene_fusion	1	1	0	1	1	1	1
EVPL&TEN1-CDK3	bidirectional_gene_fusion	0	1	0	0	0	0	1
EVPL	frameshift_variant&stop_gained&splic	1	0	0	0	0	1	1
EXPH5	frameshift_variant&stop_gained&splic	1	1	1	1	1	1	1
EXTL3	sequence_feature	1	1	1	1	1	1	1
EYS&PHF3	bidirectional_gene_fusion	1	0	0	0	0	0	0
EYS	frameshift_variant&splice_donor_vari	1	0	0	0	0	1	1
F5	frameshift_variant&splice_acceptor_v	1	0	1	1	1	1	1
F5	frameshift_variant&splice_donor_vari	0	0	0	1	1	1	1
F8	frameshift_variant&splice_acceptor_v	1	1	1	1	1	1	1
FAAH2&UBQLN2	gene_fusion	0	0	0	0	0	0	1
FAM110C	splice_donor_variant&duplication&spl	1	0	1	1	1	0	0
FAM114A1&KLF3	gene_fusion	0	0	1	0	1	1	1
FAM114A1&TLR10	bidirectional_gene_fusion	1	1	0	1	1	0	0
FAM117A	frameshift_variant&splice_donor_vari	0	1	1	0	0	0	1
FAM118A&UPK3A	gene_fusion	0	1	0	0	0	0	0
FAM120B	sequence_feature	1	1	1	0	1	1	1
FAM134B&ZNF622	gene_fusion	1	1	1	0	0	0	0
FAM149A&SORBS2	bidirectional_gene_fusion	0	0	1	0	1	0	0
FAM153C&N4BP3	gene_fusion	1	0	1	1	1	0	0
FAM156B&GPR173	gene_fusion	1	1	1	1	1	1	1
FAM171A1&NMT2	gene_fusion	1	1	1	1	1	0	0
FAM177B&TLR5	bidirectional_gene_fusion	1	1	1	1	1	0	0
FAM181B	frameshift_variant	0	1	0	0	0	0	1
FAM186A	duplication	1	1	1	1	1	0	0
FAM186A	stop_gained&duplication	0	1	1	1	1	0	1
FAM200B	sequence_feature	0	1	0	0	0	0	0

FAM205A	frameshift_variant	1	1	1	1	1	1	1
FAM205B	downstream_gene_variant	1	1	1	1	1	1	1
FAM208B&RP11-336A10.2	bidirectional_gene_fusion	1	1	1	1	1	0	0
FAM214A	frameshift_variant	0	1	0	0	0	0	0
FAM214B&RP11-182N22.7	gene_fusion	1	1	1	1	1	0	1
FAM220A	frameshift_variant&stop_lost	1	0	0	0	0	0	0
FAM27C&FAM27E4&RP11-16(	RNA_feature_ablation	0	0	0	0	0	0	1
FAM35DP	intron_variant	0	0	0	0	1	0	0
FAM45A	upstream_gene_variant	0	0	1	0	1	0	1
FAM46D&TBX22	gene_fusion	1	1	0	1	1	1	1
FAM47B&MAGEB16	gene_fusion	0	0	0	0	0	1	1
FAM47C&FTH1P18	duplication	0	0	0	0	0	1	1
FAM66A&RP11-351I21.7	bidirectional_gene_fusion	1	1	1	1	1	1	1
FAM71B&HAVCR2	gene_fusion	0	1	0	1	1	0	0
FAM71B	frameshift_variant&stop_gained	1	0	1	0	1	0	1
FAM90A21P&FAM90A6P	gene_fusion	1	1	1	1	1	1	1
FASN	frameshift_variant	1	1	0	0	0	1	1
FAS	sequence_feature	1	1	1	1	1	0	1
FAT1&TRIML2	gene_fusion	1	1	1	1	1	1	1
FAT1	frameshift_variant&splice_acceptor_v	1	1	1	0	0	1	1
FAT2	frameshift_variant	0	0	0	0	0	1	1
FAT2	frameshift_variant&start_lost	0	0	0	0	0	1	1
FAT4&SPATA5	gene_fusion	1	1	1	1	1	1	1
FAT4	sequence_feature	1	0	1	0	0	1	1
FBRSL1	sequence_feature	0	0	0	0	0	1	1
FBXL12	frameshift_variant&start_lost	0	1	0	0	0	0	0
FBXO43	frameshift_variant&splice_acceptor_v	0	0	1	0	1	0	1
FBXO45&PIGX	gene_fusion	0	1	1	0	1	0	0
FCGBP	duplication	0	0	0	0	0	0	1
FEM1C&TMED7	gene_fusion	1	0	0	1	1	1	1
FFAR1&FFAR3	gene_fusion	0	1	1	0	1	0	1
FGA	frameshift_variant&splice_acceptor_v	0	1	1	1	1	0	1
FGD2&TBC1D22B	gene_fusion	0	0	0	1	1	0	1
FGD6	frameshift_variant&splice_acceptor_v	1	1	1	0	1	0	1
FIGN&GRB14	gene_fusion	0	0	0	1	1	0	0
FIGNL1&IKZF1	bidirectional_gene_fusion	1	1	1	1	1	1	1
FIGN	splice_acceptor_variant&splice_donor	0	1	0	0	0	0	0
FILIP1L	frameshift_variant&splice_donor_vari	1	1	1	1	1	1	1
FILIP1	frameshift_variant&splice_donor_vari	1	1	1	1	1	1	1
FLG2&HRNR	gene_fusion	0	0	0	0	0	1	1
FLG2&TCHHL1	gene_fusion	1	1	1	1	1	1	1

FLG2	frameshift_variant&stop_gained&splic	0	0	1	0	1	0	0
FLNA&RPL10	bidirectional_gene_fusion	1	1	1	1	1	1	1
FLNA	frameshift_variant	0	0	1	1	1	0	0
FLNA	frameshift_variant&start_lost	0	0	0	0	0	1	1
FLNA	stop_gained&duplication	1	1	1	1	1	1	1
FLRT3	frameshift_variant&stop_gained	0	0	0	1	1	0	0
FMN1	frameshift_variant	0	0	1	1	1	0	0
FMN1	frameshift_variant&splice_acceptor_v	0	0	1	1	1	0	0
FMNL2&STAM2	bidirectional_gene_fusion	0	0	0	0	0	0	1
FNDC1	sequence_feature	1	1	1	1	1	0	1
FNIP1	frameshift_variant&splice_acceptor_v	0	0	1	0	0	0	0
FOS	protein_protein_contact	1	0	1	1	1	1	1
FOXB2&PRUNE2	bidirectional_gene_fusion	1	0	0	0	0	1	1
FOXF2&FOXQ1	gene_fusion	1	1	1	0	1	1	1
FOXR2&PAGE5	gene_fusion	0	0	0	0	0	0	1
FPR1&ZNF415	gene_fusion	0	0	0	0	0	1	1
FREM2	sequence_feature	1	1	1	1	1	1	1
FREM3&RP13-578N3.3	bidirectional_gene_fusion	1	1	1	1	1	1	1
FRMPD1	sequence_feature	0	1	0	0	0	0	0
FRMPD2P1&GDF10	gene_fusion	0	1	1	0	0	0	0
FRMPD3&FRMPD3-AS1	bidirectional_gene_fusion	0	0	1	0	0	0	0
FRMPD3	sequence_feature	1	1	0	1	1	1	1
FSIP2&ZNF804A	gene_fusion	1	0	0	1	1	1	1
FSTL5&RP11-497K21.1	bidirectional_gene_fusion	0	1	0	0	0	0	0
FUT2&SEC1P	gene_fusion	0	0	1	0	1	0	0
FUT4&PIWIL4	gene_fusion	0	0	0	1	1	0	0
FYCO1	frameshift_variant&splice_acceptor_v	0	1	0	0	0	0	1
FZD1&MTERF	bidirectional_gene_fusion	0	1	1	0	0	0	0
FZD3	sequence_feature	1	0	0	0	0	0	0
FZD8&NAMPTL	gene_fusion	0	0	1	0	1	0	0
FZD9	frameshift_variant&start_lost	0	0	1	0	0	0	0
FZD9	sequence_feature	1	0	0	0	0	0	1
GABBR1&OR2J1	bidirectional_gene_fusion	0	1	1	0	0	0	0
GABRQ&MAGEA12	bidirectional_gene_fusion	1	1	1	1	1	0	0
GAGE1&GAGE2D	gene_fusion	0	0	1	1	1	1	1
GALC&KCNK10	gene_fusion	1	1	1	1	1	0	0
GALNT13&PRPF40A	bidirectional_gene_fusion	0	1	1	1	1	0	1
GAPDHP59	transcript_ablation	1	0	0	1	1	1	1
GAPDHP61&RNU6-549P&RP1:	RNA&ZNF609_duplication	1	1	1	1	1	1	1
GAPVD1&RABEPK	gene_fusion	1	1	0	0	0	1	1
GAS2L2&RASL10B	bidirectional_gene_fusion	1	1	1	1	1	1	1

GATSL1&PMS2P5	gene_fusion	0	0	1	0	1	1	1
GBP1P1&LRRC8D	gene_fusion	0	1	1	1	1	0	1
GCLC&LRRC1	bidirectional_gene_fusion	1	0	1	1	1	0	1
GCNT2&GCNT6&RP11-360O19	duplication	0	1	0	0	0	0	1
GDF10&RBP3	gene_fusion	1	0	1	0	0	0	0
GDF9&SOWAHA	bidirectional_gene_fusion	1	0	0	1	1	0	1
GFOD2&RLTPR	bidirectional_gene_fusion	0	0	0	1	1	0	1
GGNBP2&PIGW	gene_fusion	1	1	1	1	1	0	1
GIGYF1&GNB2	bidirectional_gene_fusion	0	0	1	0	1	0	0
GIGYF1	frameshift_variant	0	0	1	0	1	0	0
GJB7&ZNF292	bidirectional_gene_fusion	1	1	1	1	1	1	1
GLB1&TRIM71	bidirectional_gene_fusion	1	1	1	1	1	0	1
GLTSCR1L&PRPH2	bidirectional_gene_fusion	0	0	1	1	1	0	1
GLTSCR1	downstream_gene_variant	1	1	0	0	1	0	1
GMNC&IL1RAP	bidirectional_gene_fusion	1	0	0	0	0	0	0
GNAS&GNAS-AS1	bidirectional_gene_fusion	1	1	1	1	1	1	1
GNG7&SLC39A3	gene_fusion	1	0	0	0	0	0	0
GNPTAB	frameshift_variant&splice_donor_vari	0	1	0	0	0	0	0
GOLGA8CP&NBEAP1	bidirectional_gene_fusion	0	1	1	1	0	0	1
GOLGA8CP&RP11-403B2.7	bidirectional_gene_fusion	1	1	1	1	1	0	0
GOLGA8F&GOLGA8G	bidirectional_gene_fusion	0	0	0	1	1	0	0
GOLGA8G	stop_gained&duplication	1	1	1	0	0	1	1
GOLGB1	frameshift_variant	1	1	1	1	1	1	1
GOLGB1	frameshift_variant&splice_acceptor_v	1	1	1	1	1	1	1
GOLGB1	frameshift_variant&splice_donor_vari	0	0	0	1	1	0	0
GOLGB1	frameshift_variant&splice_region_vari	0	0	0	0	0	1	1
GOLGB1	splice_acceptor_variant&duplication&	0	0	0	1	1	0	0
GON4L	frameshift_variant	1	1	1	1	1	0	1
GPATCH8	frameshift_variant&splice_acceptor_v	1	1	0	1	1	0	1
GPC4&HS6ST2	gene_fusion	1	1	0	1	1	0	0
GPC5&SLITRK5	gene_fusion	0	0	0	0	0	0	1
GPER1&GPR146	gene_fusion	0	1	0	0	0	0	0
GPR112	sequence_feature	1	1	0	0	1	0	1
GPR133&RAN	gene_fusion	0	0	0	1	1	0	0
GPR157&SPSB1	bidirectional_gene_fusion	1	1	1	0	1	0	0
GPR158&THNSL1	gene_fusion	0	0	0	1	1	0	1
GPR160&PHC3	bidirectional_gene_fusion	0	0	1	0	1	0	1
GPR174&LPAR4	gene_fusion	1	0	0	0	0	1	1
GPR174&ZCCHC5	bidirectional_gene_fusion	0	1	0	0	0	0	0
GPR179	frameshift_variant&stop_lost	1	1	1	1	1	1	1
GPR182&ZBTB39	bidirectional_gene_fusion	0	0	1	0	1	0	0

GPR22	sequence_feature	0	0	1	0	1	0	1
GPR34&GPR82	gene_fusion	0	1	0	0	0	0	0
GPR37L1	sequence_feature	1	1	1	0	0	0	1
GPR50	sequence_feature	1	0	0	0	0	0	0
GPR78&TRMT44	gene_fusion	1	1	1	1	1	0	1
GPRASP2&NXF2B	bidirectional_gene_fusion	1	1	1	1	1	0	0
GPRIN1	duplication	0	1	0	0	0	0	0
GPRIN3	frameshift_variant&stop_gained	0	1	0	0	0	0	0
GPRIN3	sequence_feature	1	1	0	1	1	0	1
GPSM3&NOTCH4	gene_fusion	0	1	0	1	1	0	0
GRAP	duplication	1	0	0	0	0	0	0
GRB14&KCNH7	gene_fusion	1	0	0	1	1	0	0
GREB1L	sequence_feature	0	0	1	1	1	0	0
GRIN2A	frameshift_variant&stop_gained	1	1	1	1	1	0	1
GRIN2A	splice_acceptor_variant&splice_regior	0	0	1	0	0	0	1
GRK5&RGS10	bidirectional_gene_fusion	1	0	1	1	1	0	0
GRK6&SLC34A1	gene_fusion	1	0	0	1	1	0	0
GRM1&SHPRH	bidirectional_gene_fusion	0	0	0	0	0	1	1
GRM1	sequence_feature	0	1	0	0	0	1	1
GRM3&SEMA3D	bidirectional_gene_fusion	0	0	0	0	0	1	1
GRM3	structural_interaction_variant	1	1	1	1	1	0	1
GS1-256O22.5&SLITRK2	gene_fusion	1	1	1	1	1	1	1
GSR&GTF2E2	gene_fusion	1	0	0	0	0	0	0
GSTA1&TMEM14A	bidirectional_gene_fusion	0	0	0	1	1	0	0
GTF2F2&KCTD4	bidirectional_gene_fusion	0	1	1	0	0	0	0
GTF2IRD2P1	downstream_gene_variant	1	0	0	1	1	0	1
GTSE1	intragenic_variant	0	1	1	1	1	0	1
GUCY1B2&RNASEH2B	bidirectional_gene_fusion	0	0	0	1	1	0	0
GUSBP1&RP11-823P9.3	bidirectional_gene_fusion	1	1	1	1	1	0	1
GVINP1	splice_region_variant&non_coding_tra	1	1	1	1	1	0	1
GZF1&RP4-753D10.3	gene_fusion	1	1	1	1	1	0	1
HAUS1P3&KCNB2&RP11-1145	duplication	1	1	0	1	1	0	0
HCCAT5&RP5-991G20.1	gene_fusion	0	0	0	1	1	1	1
HCCAT5&ZFHX3	bidirectional_gene_fusion	0	1	0	0	0	0	0
HDX&UBE2DNL	bidirectional_gene_fusion	0	1	0	0	0	0	1
HEATR2	downstream_gene_variant	0	1	0	0	0	1	1
HELQ	frameshift_variant&start_lost	1	0	1	1	1	0	0
HELZ2	frameshift_variant	1	1	1	1	1	1	1
HEMGN	sequence_feature	0	0	1	0	0	0	0
HEMGN	stop_gained&duplication&splice_regic	0	0	1	0	1	0	0
HERC2P4&RP11-1166P10.6	gene_fusion	1	1	1	1	1	1	1

HFM1&ZNF644	gene_fusion	0	1	0	0	0	0	0
HIPK3	sequence_feature	0	1	1	1	1	0	0
HIPK4&PRX	gene_fusion	1	0	1	1	1	1	1
HIST1H2AK	frameshift_variant&splice_region_vari	0	0	1	0	1	0	0
HIST1H2AM	frameshift_variant&start_lost&splice_	0	0	1	0	1	0	0
HIST2H2BD&HIST2H2BF	bidirectional_gene_fusion	1	1	1	1	1	1	1
HIVEP1	sequence_feature	1	1	1	1	1	0	1
HIVEP2	splice_donor_variant&duplication&spl	1	1	1	1	1	1	1
HJURP	frameshift_variant	1	1	0	0	0	0	1
HK2&SEMA4F	gene_fusion	0	1	1	1	1	0	0
HLA-DPB1&HLA-DPB2	gene_fusion	0	0	0	0	0	0	1
HMG20A&PEAK1	bidirectional_gene_fusion	0	0	0	0	0	0	1
HMGXB3&PDE6A	bidirectional_gene_fusion	0	1	1	0	0	0	0
HNRNPCP5&PRAMEF26&WI2-	duplication	1	1	1	0	0	1	1
HNRNPM	sequence_feature	1	0	1	0	0	0	1
HNRNPR&LUZP1	gene_fusion	1	1	1	1	1	1	1
HOXB3&HOXB7	gene_fusion	0	0	0	0	0	0	1
HOXD1&HOXD3	gene_fusion	0	0	0	1	1	0	0
HPDL&ZSWIM5	bidirectional_gene_fusion	0	1	1	1	1	0	0
HPS6	sequence_feature	0	1	0	0	0	0	0
HRNR&TCHHL1	gene_fusion	0	0	0	0	0	1	1
HS6ST2&TFDP3	gene_fusion	0	0	1	1	1	0	0
HSD17B8&SLC39A7	gene_fusion	1	0	0	0	0	0	0
HSD3B1&ZNF697	bidirectional_gene_fusion	1	0	0	0	0	0	1
HSD3B2	sequence_feature	0	0	1	0	1	0	0
HSFX1&MAGEA9	gene_fusion	1	0	0	0	1	0	0
HSFX2&MAGEA9B	gene_fusion	0	0	1	0	1	0	0
HSP90AB1&SLC29A1	gene_fusion	0	0	0	1	1	0	0
HSP90AB1&SLC35B2	bidirectional_gene_fusion	1	1	0	0	0	1	1
HSP90AB1	structural_interaction_variant	0	0	0	0	1	0	0
HSP90B1	sequence_feature	0	1	0	1	1	0	0
HSPA1A&HSPA1L	bidirectional_gene_fusion	1	1	1	1	1	1	1
HSPA1B&HSPA1L	bidirectional_gene_fusion	0	0	0	0	0	1	1
HSPA1B	sequence_feature	1	1	1	1	1	0	0
HSPA2&ZBTB25	bidirectional_gene_fusion	1	1	1	1	1	1	1
HYAL3&IFRD2	gene_fusion	0	0	0	0	0	1	1
IARS2	intragenic_variant	0	0	1	1	1	0	0
IBTK&UBE3D	gene_fusion	0	0	0	0	0	0	1
IFNA1&IFNA21	bidirectional_gene_fusion	1	1	1	1	1	1	1
IFNL4&LRFN1	gene_fusion	0	0	1	1	1	1	1
IGFN1	sequence_feature	1	1	1	1	1	0	0

IGFN1	upstream_gene_variant	1	1	1	1	1	1	1
IGHV1OR15-4&RP11-69H14.6	bidirectional_gene_fusion	1	1	1	1	1	0	0
IGHV3-11&IGHV3-35	gene_fusion	1	1	1	1	1	1	1
IGHV3OR16-7&RP11-812E19.3	gene_fusion	1	0	0	0	0	1	1
IGKV1OR10-1	splice_acceptor_variant&splice_region	0	1	0	0	0	0	0
IGLV1-51&PRAME	bidirectional_gene_fusion	1	0	1	0	1	1	1
IGSF1&MST4	bidirectional_gene_fusion	1	0	0	1	1	0	0
IGSF10&MED12L	bidirectional_gene_fusion	0	0	0	1	1	1	1
IGSF10	frameshift_variant	1	0	0	0	0	0	1
IKBIP&SLC25A3	bidirectional_gene_fusion	0	1	0	0	0	0	0
IKBKAP&RAD23B	bidirectional_gene_fusion	1	0	1	0	0	1	1
IL9RP4	transcript_ablation	1	1	1	1	1	0	1
ILVBL&SYDE1	bidirectional_gene_fusion	0	0	1	0	1	1	1
IMPG2	frameshift_variant	0	1	1	0	1	0	0
INHA&STK11IP	gene_fusion	1	0	0	0	0	0	0
INO80E&TAOK2	gene_fusion	1	1	1	1	1	1	1
INPP1&MFSD6	gene_fusion	1	0	0	0	0	0	0
IPO8&RNA5SP356&RP11-155I	feature_ablation	0	0	1	0	1	0	0
IQGAP2&SV2C	gene_fusion	1	1	1	1	1	1	1
IRAK1BP1&PHIP	bidirectional_gene_fusion	1	1	1	1	1	0	1
IRAK3	intragenic_variant	1	1	1	1	1	0	1
IRF2BPL	frameshift_variant&stop_gained	0	0	0	0	0	0	1
IRGQ&L34079.2	gene_fusion	0	1	1	0	1	0	0
IRS1&RHBDD1	bidirectional_gene_fusion	0	1	1	0	1	0	0
IRS4&RP6-24A23.6	gene_fusion	1	1	1	1	1	1	1
ISLR&ISLR2	gene_fusion	1	1	1	1	1	1	1
ISLR2&STRA6	bidirectional_gene_fusion	0	0	0	0	0	1	1
ITGA7&NEUROD4	bidirectional_gene_fusion	1	1	1	1	1	1	1
ITGB8&MACC1	bidirectional_gene_fusion	0	0	0	1	1	0	1
ITM2A&ZCCHC5	gene_fusion	0	0	0	1	1	0	0
ITPKB	frameshift_variant	0	0	1	0	1	0	0
ITPKB	sequence_feature	0	0	0	0	0	0	1
ITPRIPL1	splice_acceptor_variant&splice_region	0	0	0	0	0	1	1
ITPRIPL2	sequence_feature	1	1	1	1	1	0	1
JARID2&RNF182	gene_fusion	0	0	0	1	1	0	0
JMJD1C	frameshift_variant	1	1	1	1	1	0	1
JMJD1C	frameshift_variant&splice_acceptor_v	0	1	1	1	1	0	1
JMJD1C	stop_gained&duplication	0	1	0	1	1	0	0
KAT6A	frameshift_variant&stop_gained&splic	0	0	0	0	0	0	1
KAT6B	sequence_feature	0	0	0	0	0	1	1
KATNA1&LATS1	gene_fusion	1	1	0	0	0	0	0

KBTBD13&UBAP1L	bidirectional_gene_fusion	0	0	1	1	1	1	1
KBTBD8&LRIG1&RP11-814M2	feature_ablation	1	1	0	1	1	1	1
KCNB1	frameshift_variant	1	0	0	0	0	0	0
KCNE1&LINC00649	bidirectional_gene_fusion	1	1	1	1	1	0	0
KCNG4&RP11-486L19.2	gene_fusion	1	1	1	0	1	1	1
KCNJ12	sequence_feature	0	1	0	0	0	1	1
KCNQ2	frameshift_variant	0	0	1	0	1	1	1
KCNRG&TRIM13	gene_fusion	1	1	1	1	1	0	1
KCNS2&STK3	bidirectional_gene_fusion	0	0	1	0	1	0	0
KCTD4	duplication	0	0	1	0	0	0	0
KIAA0247	frameshift_variant&start_lost	1	1	1	1	1	0	0
KIAA0895	frameshift_variant&splice_acceptor_v	0	0	1	0	0	0	0
KIAA0947	sequence_feature	0	0	0	0	0	0	1
KIAA1161	frameshift_variant&stop_gained	1	1	1	1	1	1	1
KIAA1210	frameshift_variant	0	0	0	1	1	0	1
KIAA1210	stop_gained&duplication	1	1	0	0	0	0	0
KIAA1429	frameshift_variant	0	0	1	0	1	0	0
KIAA1430&SNX25	bidirectional_gene_fusion	0	1	0	0	0	0	0
KIAA1430	duplication	0	0	0	1	1	0	0
KIAA1462&MTPAP	gene_fusion	1	1	1	1	1	1	1
KIAA1462	frameshift_variant&stop_lost	0	1	0	0	0	0	0
KIAA1549L	sequence_feature	0	0	1	1	1	0	0
KIAA1551	sequence_feature	0	1	1	0	0	0	0
KIAA1586&ZNF451	gene_fusion	1	1	1	1	1	1	1
KIAA1614	sequence_feature	0	1	1	0	1	0	0
KIAA1731	sequence_feature	1	1	1	1	1	1	1
KIAA1919	sequence_feature	0	1	1	0	1	0	1
KIAA2018	frameshift_variant&stop_lost	0	0	1	0	0	0	0
KIAA2018	stop_gained&duplication	1	1	0	1	1	1	1
KIAA2022	splice_donor_variant&duplication&spl	1	1	1	1	1	1	1
KIAA2026	frameshift_variant&start_lost	1	0	1	0	0	0	0
KIAA2026	frameshift_variant&stop_gained	1	0	1	1	1	0	0
KIAA2026	frameshift_variant&stop_lost	0	0	1	0	1	0	0
KIF14&LINC00862	gene_fusion	0	0	0	1	1	1	1
KIF14&ZNF281	gene_fusion	0	0	1	0	0	0	1
KIF16B	splice_acceptor_variant&splice_donor	0	1	1	1	1	0	1
KIF26B	sequence_feature	0	1	1	1	1	0	0
KLF11	sequence_feature	0	1	1	1	1	0	0
KLF5	sequence_feature	0	0	1	1	1	0	0
KLHDC7A	sequence_feature	0	0	1	0	1	0	1
KLHDC7B&NCAPH2	gene_fusion	1	1	1	0	1	0	1

KLHL23&PHOSPHO2	gene_fusion	0	1	1	0	1	0	1
KLHL34	frameshift_variant&stop_gained	0	1	0	1	1	0	1
KLHL38	splice_donor_variant&splice_region_v	0	1	0	0	0	0	0
KM-PA-2&SCXB	bidirectional_gene_fusion	0	0	0	1	1	0	0
KMT2C	frameshift_variant&splice_acceptor_v	0	0	1	0	0	0	0
KMT2D	frameshift_variant	1	1	1	1	1	1	1
KNOP1P1&LINC00477&MIR92	feature_ablation	0	1	0	0	0	0	0
KPRP	sequence_feature	0	1	1	1	1	0	0
KRBOX1&SNRK	gene_fusion	1	1	1	1	1	1	1
KRCC1	sequence_feature	0	0	1	0	1	0	0
KRT8P44&PACRG&PARK2&RP	feature_ablation	0	0	0	0	0	1	1
KRT8P44	transcript_ablation	0	0	1	1	1	0	0
KRTAP10-11&KRTAP10-4	gene_fusion	1	0	1	0	1	1	1
KRTAP10-11&KRTAP10-7	gene_fusion	0	0	1	0	0	0	0
KRTAP4-6&KRTAP4-9	bidirectional_gene_fusion	0	0	0	0	0	1	1
KTI12&TXNDC12	gene_fusion	1	1	1	1	1	0	1
LA16c-313D11.12&RAB11FIP3	gene_fusion	0	1	1	0	0	0	0
LAMA5	frameshift_variant	1	0	1	0	1	0	1
LAMA5	frameshift_variant&stop_gained	0	0	0	1	1	1	1
LAMB4	frameshift_variant	1	0	0	0	0	0	0
LAMC1&SHCBP1L	bidirectional_gene_fusion	0	0	1	0	1	0	0
LATS1	frameshift_variant	0	0	0	1	1	0	0
LAX1&ZC3H11A	gene_fusion	1	1	1	1	1	0	1
LCE1D	start_lost&duplication	0	0	0	1	1	0	0
LCMT2	frameshift_variant&stop_gained	1	1	1	1	1	0	1
LCORL	frameshift_variant&stop_gained&splic	1	1	1	1	1	1	1
LCT	frameshift_variant	0	0	0	1	1	0	0
LHX3&QSOX2	gene_fusion	0	0	1	0	1	0	0
LIG4	stop_gained&duplication	1	1	1	1	1	0	0
LINC00116	transcript_ablation	0	1	1	1	1	0	0
LINC00482	downstream_gene_variant	1	0	0	0	0	0	0
LINC00842&RP11-38L15.2	gene_fusion	1	1	1	1	1	1	1
LINC00851&ZNF133	gene_fusion	0	1	0	0	0	0	0
LINGO1&PEAK1	gene_fusion	0	1	1	0	0	0	0
LIPE	splice_donor_variant&splice_region_v	0	1	1	0	1	0	1
LMOD1&RP11-307B6.3	bidirectional_gene_fusion	1	1	1	1	1	0	1
LMTK3	frameshift_variant&splice_donor_vari	0	0	1	0	0	0	0
LMX1B&ZBTB34	gene_fusion	1	0	0	0	0	0	0
LPAR4&ZCCHC5	bidirectional_gene_fusion	1	1	0	1	1	1	1
LPA	frameshift_variant	1	1	0	1	1	0	0
LPA	frameshift_variant&start_lost	0	0	0	0	0	1	1

LPA	stop_gained&duplication	0	1	1	1	1	0	1
LPHN3	exon_loss_variant&splice_acceptor_va	0	0	0	0	0	1	0
LRCH2&RBMXL3	bidirectional_gene_fusion	0	1	1	0	1	0	1
LRFN1	frameshift_variant&start_lost	1	1	1	0	0	0	0
LRIG1&SLC25A26	bidirectional_gene_fusion	0	0	1	1	1	0	1
LRIG2&MAGI3	gene_fusion	0	0	1	0	1	0	0
LRP3&SLC7A10	bidirectional_gene_fusion	0	0	1	0	0	0	0
LRRC34&LRRIQ4	bidirectional_gene_fusion	0	0	1	0	0	0	0
LRRC37A4P	duplication	1	0	1	1	1	0	1
LRRC4C	frameshift_variant	1	1	1	0	1	0	0
LRRC53	stop_gained&duplication	1	1	1	1	1	1	1
LRRC66	sequence_feature	1	1	0	1	1	0	0
LRRC8B&LRRC8D	gene_fusion	1	0	1	0	0	1	1
LRRD1	frameshift_variant&start_lost&splice_	0	1	0	0	0	0	1
LRRN3	sequence_feature	1	1	0	0	0	0	0
LZTS3&UBOX5	gene_fusion	0	1	0	1	1	0	0
MACC1	frameshift_variant	0	1	1	0	0	0	0
MACC1	frameshift_variant&splice_acceptor_v	1	0	0	0	0	0	0
MACC1	stop_gained&duplication	0	1	0	0	0	0	0
MAGEA1&TREX2	gene_fusion	1	1	1	1	1	1	1
MAGEC1	sequence_feature	1	1	1	1	1	0	0
MAGEE1	sequence_feature	1	1	1	1	1	1	1
MAGEE2&TTC3P1	gene_fusion	0	1	1	1	1	0	1
MAGI1	frameshift_variant&stop_gained&splic	0	1	1	1	1	0	1
MAGOH	splice_donor_variant&splice_region_v	1	0	1	0	0	0	0
MALAT1	duplication	1	1	1	1	1	1	1
MAMDC4	sequence_feature	1	1	1	1	1	1	1
MAN1B1	structural_interaction_variant	1	1	1	1	1	1	1
MAP10&SIPA1L2	bidirectional_gene_fusion	1	1	0	0	1	0	1
MAP3K19	frameshift_variant&splice_donor_vari	1	1	1	1	1	0	1
MAP3K7&MDN1	gene_fusion	0	0	0	0	0	0	1
MAP4	frameshift_variant	1	1	1	1	1	1	1
MAPK7	protein_protein_contact	0	1	1	0	1	0	1
MARS&MBD6	gene_fusion	1	1	0	1	1	0	1
MARS2	sequence_feature	0	0	0	0	0	1	1
MARVELD3&PHLPP2	bidirectional_gene_fusion	1	1	1	1	1	0	0
MAST2	sequence_feature	0	1	0	0	0	0	0
MASTL	sequence_feature	0	0	0	1	1	0	1
MAU2&RP11-15H20.6	bidirectional_gene_fusion	0	0	1	0	0	0	0
MAU2&UQCRFS1	bidirectional_gene_fusion	0	0	1	0	0	0	0
MBTPS2&YY2&Y	RNA_duplication	0	1	1	1	1	0	1

MCHR2&PRDM13	bidirectional_gene_fusion	1	1	1	1	1	0	1
MCHR2&SIM1	gene_fusion	0	1	0	0	0	0	0
MCL1	frameshift_variant&splice_donor_vari	1	0	1	0	1	1	1
MCM3&PAQR8	bidirectional_gene_fusion	0	1	0	0	0	0	0
MCM9	frameshift_variant&splice_acceptor_v	1	1	1	1	1	0	1
MCPH1	sequence_feature	1	0	1	0	0	0	0
MDC1&MDC1-AS1	bidirectional_gene_fusion	1	1	1	1	1	1	1
MECOM	frameshift_variant&splice_acceptor_v	1	0	0	0	0	0	0
MECP2	frameshift_variant&stop_gained	1	0	1	0	0	0	0
MED14-AS1&USP9X	gene_fusion	0	1	0	0	0	0	0
MED1	frameshift_variant	1	1	1	0	1	1	1
MEGF8	sequence_feature	0	1	1	0	1	0	1
METRNL	splice_acceptor_variant&splice_regior	1	1	0	1	1	0	1
MGAM	structural_interaction_variant	0	0	0	1	1	0	0
MGAT1&ZFP62	gene_fusion	1	1	1	1	1	0	1
MGST2&SCOC	gene_fusion	0	0	0	1	1	0	0
MIB2	sequence_feature	0	0	0	0	0	1	1
MINK1&RNF167	gene_fusion	1	1	1	1	1	0	1
MIR3179-1&NOMO1	duplication	0	0	1	0	1	0	0
MIR3648&MIR3687	duplication	1	1	1	1	1	0	0
MIR432&RTL1	bidirectional_gene_fusion	0	0	1	0	1	1	1
MIR4508&NPAP1	bidirectional_gene_fusion	0	0	0	1	1	0	0
MIR4509-2&MIR4509-3&RP11	duplication	1	1	1	0	0	1	1
MIR548I3&RP11-52B19.1&RP1	feature_ablation	1	0	0	0	0	0	1
MIR566&SEMA3F	duplication	0	0	1	0	1	0	0
MIR588&PRELID1P1&RNU6-2(	feature_ablation	0	1	0	0	0	0	0
MKI67	frameshift_variant&splice_acceptor_v	1	1	1	1	1	1	1
MLIP	sequence_feature	0	1	0	0	0	0	0
MLLT4-AS1	duplication	0	1	0	0	0	0	0
MMP11	sequence_feature	0	1	1	0	1	1	1
MMRN1	sequence_feature	0	1	0	0	0	0	0
MN1	frameshift_variant	1	1	1	1	1	0	1
MOB1A&MTHFD2	bidirectional_gene_fusion	0	1	1	0	1	0	0
MRGPRE&MRGPRG	gene_fusion	0	0	1	1	1	0	0
MRGPRE&OSBPL5	gene_fusion	1	1	1	0	0	1	1
MRPL18&TCP1	bidirectional_gene_fusion	0	0	0	1	1	0	1
MSH6	protein_protein_contact	0	0	0	0	0	1	1
MST1P2&NBPF1	bidirectional_gene_fusion	1	1	1	0	0	1	1
MSX2P1&OR4D1&OR4D2	duplication	0	1	0	1	1	0	1
MTUS1	frameshift_variant	1	1	0	0	1	1	1
MTUS1	stop_gained&duplication	0	0	0	0	0	1	0

MUC12	sequence_feature	1	1	1	1	1	1	1
MUC16	frameshift_variant	1	1	1	1	1	1	1
MUC16	frameshift_variant&splice_donor_vari	1	1	1	1	1	0	0
MUC16	frameshift_variant&start_lost	0	0	0	0	0	1	0
MUC16	splice_acceptor_variant&splice_donor	1	1	1	1	1	1	1
MUC17	sequence_feature	1	1	1	1	1	1	1
MUC19	downstream_gene_variant	1	1	1	1	1	1	1
MUC22	sequence_feature	1	1	1	1	1	1	1
MUC4&TNK2	gene_fusion	0	1	0	0	0	1	1
MUC4	frameshift_variant	0	0	1	0	1	0	0
MUC4	frameshift_variant&splice_donor_vari	0	1	0	0	0	0	0
MUC5B&RP11-532E4.2	bidirectional_gene_fusion	0	0	1	1	1	1	1
MUTYH&ZSWIM5	gene_fusion	1	0	0	0	1	0	1
MUTYH	frameshift_variant	0	1	1	1	1	0	0
MYADM	sequence_feature	0	0	1	0	1	1	1
MYBPH&MYOG	gene_fusion	1	0	0	0	1	0	1
MYO15A	upstream_gene_variant	1	1	1	1	1	0	1
MYO9A&SENP8	bidirectional_gene_fusion	1	0	0	1	1	0	0
MYO9A	frameshift_variant	0	0	0	1	1	0	0
N4BP1	frameshift_variant&splice_donor_vari	1	1	1	1	1	0	1
N4BP3	sequence_feature	0	1	0	1	1	0	0
NAF1&TKTL2	gene_fusion	0	1	1	1	1	1	1
NAIP	frameshift_variant&splice_donor_vari	1	1	0	1	1	0	1
NALCN	splice_donor_variant&splice_region_v	0	0	0	1	1	0	0
NAP1L3&PCDH11X	bidirectional_gene_fusion	0	1	1	0	1	0	1
NARG2	frameshift_variant&splice_acceptor_v	1	0	0	1	1	0	0
NBPF10&PDE4DIP	bidirectional_gene_fusion	1	1	1	1	1	1	1
NBPF1	frameshift_variant	1	1	1	0	0	1	1
NBPF1	frameshift_variant&stop_gained	1	1	1	1	1	1	1
NCKAP5L	frameshift_variant&splice_donor_vari	1	1	1	1	1	1	1
NCKAP5	frameshift_variant&splice_acceptor_v	1	1	1	1	1	1	1
NCOA6	frameshift_variant&splice_donor_vari	1	1	1	1	1	1	1
NDFIP1&SPRY4	bidirectional_gene_fusion	0	1	1	1	1	0	1
NDOR1&RNF208	bidirectional_gene_fusion	0	1	0	0	0	0	0
NDST3&NDST4	bidirectional_gene_fusion	0	1	0	0	0	0	0
NEDD4	duplication	1	1	1	1	1	1	1
NEDD4	frameshift_variant	0	1	1	1	1	0	0
NEFL	duplication	0	1	0	1	1	0	1
NES	frameshift_variant&stop_gained	1	1	1	1	1	1	1
NEURL1&SH3PXD2A	bidirectional_gene_fusion	0	0	1	0	0	0	0
NEURL4	frameshift_variant&stop_gained	0	1	1	0	1	0	1

NFKBIB	sequence_feature	0	1	0	1	1	0	0
NHSL1	frameshift_variant	0	0	1	0	0	0	0
NHSL1	frameshift_variant&stop_gained&splic	1	1	0	1	1	0	1
NIN	frameshift_variant	1	1	0	1	1	0	0
NISCH	sequence_feature	1	0	1	0	0	0	1
NKAP&ZBTB33	bidirectional_gene_fusion	1	1	1	1	1	1	1
NKRF&SEPT6	gene_fusion	0	0	0	1	1	0	0
NKX2-2&PAX1	bidirectional_gene_fusion	0	1	0	0	0	0	1
NLGN4X	frameshift_variant	0	0	1	0	1	0	0
NMD3P2&RBM22P3&RNU6-1(	feature_ablation	1	1	1	1	1	1	1
NOC3L&TBC1D12	bidirectional_gene_fusion	0	0	0	0	0	0	1
NOL12	intragenic_variant	1	1	1	0	1	0	1
NOMO2	frameshift_variant&stop_gained&splic	1	1	0	0	0	0	0
NOMO3&RP11-517A5.6	duplication	0	0	0	0	0	0	1
NOTCH2&RP11-439A17.7	gene_fusion	1	1	1	1	1	0	1
NPAP1	sequence_feature	1	1	1	1	1	1	1
NPAP1	start_lost&duplication	1	0	1	0	1	0	0
NPAT	splice_acceptor_variant&splice_donor	0	0	1	0	0	0	0
NPAT	stop_gained&duplication	0	0	1	0	0	0	0
NPBWR1	sequence_feature	0	1	0	0	0	0	0
NPIPB11	duplication	1	0	1	0	0	1	1
NPIPB11	frameshift_variant&stop_gained	0	0	0	0	1	0	0
NPIPB3	frameshift_variant&stop_gained	1	1	1	1	1	1	1
NPIPB3	frameshift_variant&stop_gained&splic	0	1	1	1	1	1	1
NPIPB4	frameshift_variant	1	1	1	1	1	1	1
NPIPB4	stop_gained&duplication	0	0	0	0	0	1	1
NPIPB5	sequence_feature	1	1	1	1	1	1	1
NR3C1	splice_acceptor_variant&splice_regior	0	0	0	1	1	0	0
NRBP2&PLEC	gene_fusion	1	0	1	0	1	1	1
NRG3	sequence_feature	0	0	1	0	0	0	0
NRIP1	sequence_feature	1	1	1	1	1	0	1
NRXN1	frameshift_variant&splice_donor_vari	0	1	0	0	0	0	0
NSUN5P2&POM121	bidirectional_gene_fusion	1	1	0	1	1	0	0
NSUN5P2	downstream_gene_variant	0	1	0	0	0	0	1
NSUN5P2	duplication	0	1	1	0	1	0	1
NT5C1B-RDH14&OSR1	gene_fusion	1	0	1	1	1	0	1
NT5C1B-RDH14&SMC6	gene_fusion	1	0	1	1	1	0	1
NTN1	sequence_feature	0	0	0	0	0	0	1
NUDT17	duplication	1	0	0	0	0	0	0
NUFIP2	splice_acceptor_variant&splice_donor	1	0	0	1	1	0	1
NUMA1&RP11-849H4.4	bidirectional_gene_fusion	1	1	1	1	1	1	1

NUP205	sequence_feature	0	0	0	0	1	0	0
NUP214	sequence_feature	1	0	1	0	0	0	1
NUSAP1&OIP5	bidirectional_gene_fusion	1	0	1	1	1	1	1
NXF2&NXF5	bidirectional_gene_fusion	1	1	1	1	1	0	1
NXF2&RPL36A-HNRNPH2	gene_fusion	0	0	0	0	0	1	0
NYAP2&RHBDD1	gene_fusion	1	0	1	1	1	0	1
NYAP2	sequence_feature	0	0	1	0	1	0	0
OCRL&TENM1	bidirectional_gene_fusion	1	1	1	1	1	0	1
OLFML2A&WDR38	gene_fusion	1	0	0	0	0	0	1
OLIG3&PERP	gene_fusion	0	0	1	0	0	0	0
OPLAH&PLEC	gene_fusion	1	0	0	0	0	1	1
OR10D1P&OR10D3&OR8F1P&	duplication	1	1	1	1	1	0	0
OR10G7&OR8D4	bidirectional_gene_fusion	1	1	1	1	1	1	1
OR10K1&OR6Y1	bidirectional_gene_fusion	1	0	0	0	0	0	0
OR11H2&OR11K2P	gene_fusion	0	0	1	1	1	0	1
OR14C36&OR2AJ1&OR2AK2&	RNA&Y_RNA&Y_RNA_duplication	1	1	0	0	0	0	0
OR1AC1P&OR1E1&OR1E2&OF	duplication	1	0	0	0	0	0	0
OR1L1&OR1L4	gene_fusion	1	0	0	0	0	0	0
OR2A13P&OR2F2	gene_fusion	1	1	1	0	0	1	1
OR2AS1P&OR2AS2P&OR2G6&	duplication	0	0	1	1	1	1	1
OR2D3&RP11-732A19.5	gene_fusion	0	0	0	0	0	1	1
OR2F2&RP4-545C24.1	gene_fusion	0	0	0	1	1	1	1
OR2H1&OR2J1	gene_fusion	1	1	1	1	1	1	1
OR2J1&OR5V1	bidirectional_gene_fusion	0	0	0	0	0	1	1
OR2L13&OR2L2	gene_fusion	0	0	1	1	1	0	1
OR2L13&OR2T7	gene_fusion	0	0	1	1	1	0	0
OR2T7&OR2T8	gene_fusion	0	0	0	0	0	1	1
OR4C3&PTPRJ	gene_fusion	1	1	1	1	1	0	1
OR4F13P&OR4F14P&OR4F15&	duplication	1	1	0	1	1	0	0
OR4F4	sequence_feature	1	0	0	1	1	0	1
OR4F7P	splice_region_variant&non_coding_tra	0	0	0	1	1	0	0
OR4M1&OR4N2	gene_fusion	0	0	0	0	0	1	1
OR6K6&OR6N1	bidirectional_gene_fusion	0	1	0	1	1	0	0
OR6Y1	frameshift_variant&stop_gained&splic	0	0	0	1	1	0	0
OR7E157P&RP11-1118M6.2	feature_ablation	1	0	0	0	0	0	0
ORM1&ORM2	gene_fusion	0	1	1	1	1	1	1
OSBPL7&TBX21	bidirectional_gene_fusion	0	0	0	0	0	0	1
OSCAR	upstream_gene_variant	0	0	1	0	1	0	0
OSGEP&TEP1	gene_fusion	1	1	1	1	1	0	1
OSR2	sequence_feature	0	0	1	0	1	0	0
OTOA	sequence_feature	1	0	0	0	0	0	0

OTOG	sequence_feature	0	1	1	0	1	0	0
OTUD7B	stop_gained&duplication&splice_regic	0	1	0	1	1	0	0
OVCH1-AS1	upstream_gene_variant	0	0	0	1	1	0	0
P2RY11&PPAN-P2RY11	gene_fusion	0	1	0	0	0	0	0
PALB2	frameshift_variant&stop_gained	0	0	0	0	0	0	1
PAPSS2	frameshift_variant&splice_region_vari	0	0	0	0	0	0	1
PAQR8	sequence_feature	1	0	0	0	1	0	0
PARGP1	intragenic_variant	1	0	0	0	0	0	0
PARP10	structural_interaction_variant	0	0	1	0	1	0	0
PARP4	frameshift_variant&start_lost	0	0	0	0	0	0	1
PARS2	frameshift_variant	1	0	0	0	0	0	0
PAX1	upstream_gene_variant	1	1	1	1	1	0	1
PCDH10&PCDH18	bidirectional_gene_fusion	1	1	1	1	1	0	0
PCDH10&SLC7A11-AS1	gene_fusion	0	1	1	0	0	0	0
PCDH11X	sequence_feature	0	1	1	0	1	0	1
PCDH12	frameshift_variant	0	0	0	0	0	1	1
PCDH19	frameshift_variant&splice_donor_vari	0	1	1	0	1	0	1
PCDH7	sequence_feature	1	0	1	0	0	1	1
PCDH9	frameshift_variant&splice_donor_vari	0	1	1	1	1	0	0
PCDHA1&PCDHGB3	gene_fusion	1	1	1	0	0	1	1
PCDHGA1	frameshift_variant	0	0	1	1	1	0	0
PCED1B&SLC38A4	bidirectional_gene_fusion	0	0	1	0	0	0	0
PCF11	sequence_feature	1	0	0	0	0	0	0
PCLO&SEMA3E	gene_fusion	1	1	1	1	1	1	1
PDCD6IP	structural_interaction_variant	1	1	1	0	1	0	1
PDE11A&RBM45	bidirectional_gene_fusion	0	0	1	0	1	0	1
PDE3B&RP11-677N16.1	duplication	0	1	0	0	0	0	1
PDE6A&TIGD6	gene_fusion	1	1	1	1	1	1	1
PDIA3P1	upstream_gene_variant	1	0	1	0	0	0	0
PDK3&SUPT20HL2	bidirectional_gene_fusion	0	0	0	0	0	0	1
PDPK1	structural_interaction_variant	1	1	1	1	1	1	1
PDPR&RP11-296I10.3	bidirectional_gene_fusion	0	0	1	0	0	0	0
PDXDC2P&RP11-296I10.3	gene_fusion	0	0	1	0	1	0	1
PDZD2	sequence_feature	1	1	1	1	1	0	1
PDZD8&SLC18A2	bidirectional_gene_fusion	0	1	0	0	0	0	0
PDZD8	frameshift_variant&stop_gained&splic	1	0	1	1	1	1	1
PDZRN3&PPP4R2	bidirectional_gene_fusion	0	0	1	0	0	0	0
PDZRN3	frameshift_variant&stop_gained&splic	1	1	0	1	1	0	1
PEAK1	splice_donor_variant&5_prime_UTR_v	1	0	1	1	1	1	1
PEAK1	stop_gained&duplication	0	0	0	0	0	0	1
PEAR1	sequence_feature	1	1	0	0	0	0	0

PEG10&PPP1R9A	gene_fusion	0	1	1	0	0	0	0
PEG10	sequence_feature	1	1	1	1	1	1	1
PEG3	frameshift_variant	1	1	1	1	1	1	1
PER3&RP3-467L1.4	bidirectional_gene_fusion	1	1	1	0	1	0	0
PEX1	frameshift_variant&splice_acceptor_v	0	0	0	0	0	0	1
PFN2&TSC22D2	bidirectional_gene_fusion	1	0	1	1	1	0	1
PGR&TRPC6	gene_fusion	0	0	0	0	0	0	1
PHC1	sequence_feature	1	0	1	0	1	0	1
PHKG1P4&SLC29A4P1&TNRC1	feature_ablation	1	1	1	1	1	1	1
PHLDA3	frameshift_variant&start_lost	1	1	1	1	1	0	0
PHLDB1	sequence_feature	0	0	1	0	1	0	0
PIAS4&ZBTB7A	bidirectional_gene_fusion	0	0	1	0	1	0	0
PIDD&SLC25A22	gene_fusion	1	0	0	0	1	1	1
PIK3C2B&PPP1R15B	gene_fusion	1	1	0	0	1	0	0
PIPSL&PLCE1	bidirectional_gene_fusion	1	1	1	1	1	1	1
PIPSL&SLC35G1	bidirectional_gene_fusion	1	1	1	1	1	1	1
PIWIL4&RP11-867G2.8	bidirectional_gene_fusion	0	1	1	0	0	0	1
PJA1	splice_acceptor_variant&splice_regior	1	0	1	0	1	0	0
PKDREJ	duplication	1	1	1	1	1	1	1
PKDREJ	frameshift_variant	0	1	1	0	0	0	0
PKN2	structural_interaction_variant	1	0	0	1	1	1	1
PLAGL1&STX11	bidirectional_gene_fusion	0	1	0	0	0	0	0
PLCH1	frameshift_variant&splice_acceptor_v	0	1	1	1	1	0	0
PLCH1	frameshift_variant&stop_gained	1	1	1	1	1	0	0
PLCXD3	frameshift_variant&start_lost	0	0	1	0	1	0	0
PLD3&PRX	bidirectional_gene_fusion	0	1	1	0	1	0	1
PLEC	splice_donor_variant&splice_region_v	1	0	0	0	0	0	0
PLEKHA3&TTN-AS1	gene_fusion	1	1	1	0	1	0	0
PLEKHG2	sequence_feature	0	1	1	0	1	0	1
PLEKHG2	upstream_gene_variant	1	1	0	0	1	0	1
PLIN4	frameshift_variant&splice_acceptor_v	1	1	1	1	1	1	1
PLIN4	splice_donor_variant&duplication&spl	0	1	1	1	1	1	1
PLSCR5&RNU6-505P&RP11-23	feature_ablation	1	1	0	0	0	0	1
PLXNA3	sequence_feature	1	1	1	1	1	1	1
PNISR	frameshift_variant&stop_gained&splic	0	1	0	0	0	0	0
PNMA3&PNMA5	bidirectional_gene_fusion	1	1	1	1	1	0	0
PNMA3&ZNF185	gene_fusion	0	0	1	0	1	0	1
PNPLA8&THAP5	gene_fusion	1	1	0	1	1	0	1
POLA1	structural_interaction_variant	0	0	0	0	0	0	1
POLQ	frameshift_variant&splice_acceptor_v	1	1	1	1	1	1	1
POLQ	frameshift_variant&splice_donor_vari	0	1	1	1	1	1	1

POLR2A	downstream_gene_variant	0	1	0	0	0	0	0
POM121L12&VSTM2A	gene_fusion	0	1	1	0	1	0	0
POMZP3	splice_acceptor_variant&duplication&	1	0	0	0	0	0	0
POSTN	frameshift_variant	1	0	0	0	0	0	0
POTEH	frameshift_variant&splice_acceptor_v	0	0	0	1	1	0	0
POTEH	frameshift_variant&start_lost	1	1	1	1	1	1	1
POTEH	start_lost&duplication	0	0	0	1	1	0	0
POTEM&RP11-597A11.1	bidirectional_gene_fusion	0	0	0	1	1	0	0
POTEM	stop_gained&duplication	1	1	1	1	1	1	1
POU4F3&TCERG1	gene_fusion	0	0	0	1	1	0	0
PPAN&PPAN-P2RY11&SNORD:	duplication	0	1	0	0	0	0	0
PPIH	structural_interaction_variant	0	1	1	1	1	0	1
PPIP5K1	downstream_gene_variant	1	1	1	1	1	1	1
PPL&UBN1	bidirectional_gene_fusion	0	0	1	1	1	0	1
PPL	frameshift_variant&stop_gained&splic	0	1	0	0	0	0	0
PPP1R3A&TSRM	bidirectional_gene_fusion	0	1	1	0	0	0	0
PPP1R3A	frameshift_variant&start_lost	1	0	0	1	1	0	1
PPRC1	sequence_feature	0	1	0	0	0	0	0
PRB2&PRB4	gene_fusion	0	0	0	0	0	0	1
PRCP&RP11-179A16.1	gene_fusion	1	0	0	1	1	0	0
PRDM2	sequence_feature	0	0	0	0	0	1	0
PRR12	sequence_feature	1	0	1	1	1	0	1
PRR14L	splice_donor_variant&duplication&spl	1	1	1	1	1	1	1
PRRC2A	sequence_feature	0	0	1	0	1	0	0
PRRT1	frameshift_variant	0	1	1	1	1	0	1
PRSS37&SSBP1	bidirectional_gene_fusion	1	1	1	1	1	1	1
PRUNE2	stop_gained&duplication	1	1	1	1	1	1	1
PSAPL1&SORCS2	bidirectional_gene_fusion	0	1	0	0	0	0	0
PSAPL1	frameshift_variant	1	0	0	0	0	0	0
PSD2&UBE2D2	gene_fusion	1	0	0	1	1	0	1
PTCHD2&UBIAD1	gene_fusion	0	0	1	0	1	0	0
PTGER4	sequence_feature	1	1	0	0	0	0	0
PTPN23&SCAP	bidirectional_gene_fusion	1	1	1	1	1	1	1
PTPRH	frameshift_variant	1	0	1	1	1	0	1
PTPRZ1	protein_protein_contact	0	0	1	0	0	0	0
PTPRZ1	sequence_feature	1	1	1	1	1	1	1
PVT1&TRIB1	gene_fusion	1	1	1	1	1	0	1
PWWP2A	frameshift_variant&splice_acceptor_v	1	1	1	0	0	0	0
PWWP2A	frameshift_variant&start_lost	0	0	1	1	1	0	1
PWWP2B	structural_interaction_variant	0	0	1	0	1	0	1
QRICH2	splice_donor_variant&duplication&spl	0	0	0	0	0	1	1

QSER1	sequence_feature	1	1	1	1	1	1	1
RAB11FIP2&RP11-354M20.3	bidirectional_gene_fusion	0	0	1	0	0	0	0
RAB44	intragenic_variant	0	0	0	1	1	0	0
RABL2A	sequence_feature	0	1	0	0	0	0	0
RAD51AP2	frameshift_variant&start_lost&splice_	1	1	1	1	1	0	1
RAET1G&RP11-244K5.8	bidirectional_gene_fusion	1	1	1	1	1	0	0
RAG1&RAG2	bidirectional_gene_fusion	1	1	1	0	0	0	0
RAI1	sequence_feature	1	1	1	1	1	1	1
RANBP2	sequence_feature	1	1	1	1	1	1	1
RANBP2	upstream_gene_variant	0	0	1	0	1	0	0
RANBP6	frameshift_variant&stop_lost	1	0	1	0	0	0	0
RAPGEF6	frameshift_variant&stop_gained&splic	0	0	1	0	0	0	0
RASA4DP	downstream_gene_variant	1	1	1	0	1	0	1
RB1CC1	stop_gained&duplication	0	1	0	0	0	0	1
RBM10&UBA1	gene_fusion	0	1	1	0	1	0	1
RBM12B&TMEM67	bidirectional_gene_fusion	0	0	0	0	0	1	1
RBM12B	frameshift_variant&start_lost&splice_	0	0	1	0	1	0	0
RBMX&ZIC3	bidirectional_gene_fusion	0	0	1	0	1	0	0
RBPJL	3_prime_UTR_variant	0	0	1	0	1	0	0
RDH16&ZBTB39	gene_fusion	1	1	0	1	1	0	1
REPIN1&ZNF775	gene_fusion	0	1	1	0	1	1	1
REV3L	frameshift_variant	1	1	1	1	1	1	1
REV3L	stop_gained&duplication	0	0	0	1	1	0	0
REXO1L10P&REXO1L11P&REX	duplication	1	1	1	1	1	1	1
REXO1	frameshift_variant&splice_acceptor_v	0	0	0	1	0	0	1
REXO1	frameshift_variant&splice_region_vari	1	1	1	1	1	0	0
RFPL2	frameshift_variant	0	0	1	0	0	0	1
RFPL2	stop_gained&duplication	0	0	0	0	0	1	1
RFPL3&RFPL3S	bidirectional_gene_fusion	0	0	1	0	0	1	1
RFPL3S	duplication	0	0	1	0	0	0	0
RFPL4A&RFPL4AL1&RFPL4AP1	duplication	0	0	0	0	0	1	1
RFPL4A&RFPL4AP1	gene_fusion	1	1	1	1	1	0	0
RFX7	frameshift_variant&stop_lost	1	1	0	1	1	1	1
RFX7	splice_acceptor_variant&duplication&	0	1	0	0	0	1	0
RGAG1	sequence_feature	1	1	1	1	1	1	1
RGPD1	sequence_feature	1	1	1	1	1	1	1
RGPD2	frameshift_variant&splice_acceptor_v	1	1	1	1	1	1	1
RGPD3	frameshift_variant&splice_acceptor_v	1	1	1	1	1	1	1
RGPD4	sequence_feature	1	1	1	1	1	1	1
RGPD5	downstream_gene_variant	1	1	1	1	1	0	0
RGPD5	upstream_gene_variant	1	1	1	1	1	1	1

RGPD6	frameshift_variant	1	1	1	1	1	1	1
RGPD6	frameshift_variant&splice_acceptor_v	1	1	1	1	1	1	1
RGPD8	frameshift_variant	0	0	0	1	1	0	0
RGPD8	frameshift_variant&splice_acceptor_v	1	1	1	1	1	1	1
RGPD8	frameshift_variant&stop_gained	0	1	1	0	0	0	0
RGS14&SLC34A1	gene_fusion	0	0	0	1	1	0	0
RMI1&RP11-380F14.2	gene_fusion	0	0	1	0	1	0	1
RMND5B	splice_acceptor_variant&duplication&	1	1	1	1	1	0	0
RN7SKP235	transcript_ablation	0	0	0	0	0	1	1
RNF113B	frameshift_variant&start_lost	1	1	1	1	1	0	1
RNF139&TRMT12	gene_fusion	1	1	0	1	1	0	1
RNF168&WDR53	gene_fusion	0	1	0	0	0	0	0
RNF168	frameshift_variant&stop_lost	0	1	1	0	1	0	0
RNF213	sequence_feature	1	1	0	0	0	0	0
RNF41&RP11-977G19.5	gene_fusion	0	0	1	0	0	0	0
RNMT&ZNF519	bidirectional_gene_fusion	1	1	1	0	0	1	1
RNU6-767P	upstream_gene_variant	0	1	1	1	1	1	1
ROBO1	stop_gained&duplication	0	0	0	1	1	0	0
RP1-125N5.2&RP11-417E7.1&	A.2_feature_ablation	0	0	0	0	0	0	1
RP1-232L22	_B.1&RPL36A-HNRNPH2_bidirectional	1	1	1	0	0	1	1
RP11-1023L17.1&RP11-1023L	bidirectional_gene_fusion	1	1	1	1	1	1	1
RP11-1023L17.1	upstream_gene_variant	0	0	1	0	1	0	1
RP11-1094M14.11&SLFN5	gene_fusion	1	1	1	0	1	1	1
RP11-1094M14.11&UNC45B	gene_fusion	0	0	0	1	1	0	0
RP11-126K1.2&ZNF687	bidirectional_gene_fusion	0	0	0	0	0	1	1
RP11-1280N14.3	splice_donor_variant&splice_region_v	1	1	1	0	0	0	1
RP11-1319K7.1	splice_donor_variant&splice_region_v	0	1	0	0	0	1	1
RP11-149F8.3&RP11-195B21.3	feature_ablation	0	0	0	1	1	0	0
RP11-151E14.1	transcript_ablation	0	0	0	0	0	0	1
RP11-153F1.2&RP11-347K2.1	gene_fusion	0	0	1	0	1	0	0
RP11-154H17.1	splice_donor_variant&splice_region_v	1	1	1	1	1	1	1
RP11-154P18.2	splice_acceptor_variant&splice_regior	0	0	0	1	1	0	1
RP11-156P1.2	downstream_gene_variant	1	1	0	1	1	1	1
RP11-171I2.2&TTN-AS1	gene_fusion	1	0	0	0	0	0	0
RP11-192P3.5&ZEB1	bidirectional_gene_fusion	0	1	1	1	1	0	1
RP11-231C14.4&SNX29P2	bidirectional_gene_fusion	1	1	1	1	1	1	1
RP11-244K5.8&ULBP2	gene_fusion	0	0	0	0	0	0	1
RP11-255H23.4&ZNF681	gene_fusion	0	0	0	0	0	0	1
RP11-291L22.4	splice_acceptor_variant&splice_regior	0	0	0	0	0	1	0
RP11-296E7.1	non_coding_transcript_exon_variant	1	1	1	1	1	0	1
RP11-296I10.3	splice_acceptor_variant&splice_regior	1	1	0	1	1	1	1

RP11-296I10.3	upstream_gene_variant	0	1	1	0	0	0	0
RP11-29H23.5&YY1AP1	gene_fusion	0	0	1	1	1	0	0
RP11-347C12.1	frameshift_variant	0	1	1	1	1	0	0
RP11-347C12.1	splice_acceptor_variant&duplication&	1	1	1	1	1	1	1
RP11-34F13.3	upstream_gene_variant	0	1	1	1	1	1	1
RP11-363G10.2	duplication	1	1	1	0	1	0	1
RP11-383M4.2&RP11-383M4.	gene_fusion	0	0	0	0	0	0	1
RP11-383M4.6	splice_acceptor_variant&splice_donor	0	0	0	0	0	0	1
RP11-386G11.5&WNT1	gene_fusion	1	1	1	1	1	1	1
RP11-395E19.5&SPATA31A3	bidirectional_gene_fusion	0	0	0	1	1	0	0
RP11-402G3.3&TNFSF15	gene_fusion	1	0	0	0	0	0	0
RP11-432B6.3	frameshift_variant&start_lost&splice_	0	0	1	0	1	0	0
RP11-43D2.2&ZNF608	bidirectional_gene_fusion	1	0	0	0	0	0	0
RP11-446F17.3&TDRD6	bidirectional_gene_fusion	1	1	1	0	1	0	0
RP11-492D6.3	duplication	0	1	1	0	0	1	1
RP11-506D12.5&WFIKKN2	bidirectional_gene_fusion	1	0	0	0	1	0	0
RP11-510H23.1	non_coding_transcript_exon_variant	0	1	0	0	0	0	1
RP11-525E9.1&RP11-726G1.1	bidirectional_gene_fusion	0	0	1	0	1	1	1
RP11-538P18.1	transcript_ablation	0	0	0	0	0	1	1
RP11-589F5.4	intragenic_variant	1	1	0	1	1	0	1
RP11-624G17.3&RTN4RL2	bidirectional_gene_fusion	0	0	1	0	0	0	1
RP11-683L23.1	duplication	0	1	1	0	1	0	0
RP11-6O2.4&SYNM	bidirectional_gene_fusion	0	1	0	0	0	0	1
RP11-707O23.5	non_coding_transcript_exon_variant	0	0	0	1	1	0	0
RP11-766F14.2	duplication	1	1	1	1	1	1	1
RP11-813N20.1&UGT2B7	bidirectional_gene_fusion	1	0	1	0	1	0	0
RP11-847H18.2&SYT10	gene_fusion	1	1	0	1	1	0	0
RP11-847H18.2	splice_acceptor_variant&splice_regior	0	0	1	0	0	0	0
RP11-863K10.7&ZNF703	bidirectional_gene_fusion	1	1	1	1	1	1	1
RP11-88L24.4&TTN	bidirectional_gene_fusion	0	1	1	0	1	0	1
RP11-961A15.1&WDR81	bidirectional_gene_fusion	1	1	1	1	1	1	1
RP11-98J23.2	intragenic_variant	0	1	0	0	0	1	1
RP1L1	stop_gained&duplication	1	1	1	1	1	1	1
RP1	sequence_feature	0	0	1	0	0	0	0
RP4-576H24.5	transcript_ablation	1	1	1	1	1	1	1
RP5-937E21.8&TMEM185A	gene_fusion	1	0	0	0	0	0	0
RP5-991G20.1	splice_acceptor_variant&splice_regior	1	0	1	1	1	1	1
RPE	structural_interaction_variant	0	0	1	0	0	0	0
RPF1	sequence_feature	0	1	0	0	0	0	1
RPGR	frameshift_variant	0	0	1	0	1	0	1
RPL24&ZBTB11	gene_fusion	0	0	1	0	0	0	1

RPL31P57&TEX2	duplication	0	1	1	0	0	0	0
RPL35P8-C22orf34	intergenic_region	0	0	1	0	0	0	1
RPL5P27	transcript_ablation	1	1	1	1	1	1	1
RPRD2	sequence_feature	1	1	1	1	1	1	1
RPTOR	sequence_feature	1	1	0	0	0	0	0
RRBP1	frameshift_variant	0	1	1	0	0	0	0
RRBP1	frameshift_variant&start_lost	1	1	1	1	1	1	1
RREB1	sequence_feature	0	1	0	0	0	0	0
RSF1	frameshift_variant&splice_acceptor_v	1	1	1	1	1	0	1
RSPH3&TAGAP	gene_fusion	1	1	1	1	1	0	1
RTL1	frameshift_variant	0	0	1	0	0	0	0
RTN4	frameshift_variant&splice_acceptor_v	1	1	0	1	1	1	1
RUSC2	sequence_feature	1	1	1	1	1	1	1
RXFP4	stop_gained&duplication	1	0	0	0	1	0	0
S1PR4	sequence_feature	0	1	1	0	0	0	1
SACS	frameshift_variant&splice_donor_vari	1	1	1	1	1	1	1
SAMD8&VDAC2	gene_fusion	0	0	1	0	1	1	1
SAMD9&SAMD9L	gene_fusion	1	1	1	1	1	1	1
SAMD9	duplication	0	0	0	0	0	1	1
SBSN	frameshift_variant&splice_donor_vari	0	1	1	0	1	0	1
SCAF11	frameshift_variant	0	0	0	0	0	1	1
SCAF11	frameshift_variant&start_lost	0	0	0	0	0	1	1
SCAF11	stop_gained&duplication	1	1	1	1	1	0	1
SCAF4	frameshift_variant&stop_gained	1	0	0	0	0	0	1
SCAND3	frameshift_variant	1	1	1	1	1	1	1
SCG2	frameshift_variant&stop_lost	0	0	1	1	1	0	1
SCIMP&ZNF594	gene_fusion	1	1	1	1	1	0	0
SCN1A&SCN9A	gene_fusion	1	1	1	1	1	0	1
SCN2A	sequence_feature	1	1	1	1	1	1	1
SCNM1&TNFAIP8L2	gene_fusion	0	1	0	0	0	0	1
SCNN1D	sequence_feature	1	1	0	0	1	0	0
SDHDP6	transcript_ablation	1	1	1	1	1	1	1
SEC23A&SSTR1	bidirectional_gene_fusion	1	0	0	0	0	0	0
SEC23A&TTC6	bidirectional_gene_fusion	0	0	1	0	1	0	0
SEC24B	structural_interaction_variant	1	0	0	1	1	0	0
SEMA4C	frameshift_variant	0	0	1	0	0	0	0
SEPP1	frameshift_variant&stop_gained&splic	1	0	0	0	0	0	0
SERPINE1&TRIM56	gene_fusion	1	1	1	1	1	1	1
SETD1B	protein_protein_contact	1	1	1	1	1	1	1
SETD2	frameshift_variant&splice_donor_vari	1	1	1	1	1	1	1
SETX	frameshift_variant	1	1	1	1	1	1	1

SGK223	duplication	0	0	0	0	1	0	0
SH2D1A&STAG2	gene_fusion	0	0	1	1	1	0	0
SH3BP4	sequence_feature	1	1	1	1	1	1	1
SH3PXD2A	frameshift_variant	1	1	0	1	1	0	1
SH3TC1	sequence_feature	1	1	0	0	0	0	0
SHANK2	frameshift_variant&splice_acceptor_v	1	1	0	1	1	0	1
SHROOM2&Y	RNA_duplication	1	1	0	0	0	0	1
SHROOM2	sequence_feature	0	0	1	0	0	0	0
SHROOM3&SOWAHB	bidirectional_gene_fusion	0	0	0	1	1	0	0
SHROOM3	sequence_feature	1	1	1	0	1	1	1
SHROOM4	frameshift_variant&splice_acceptor_v	0	0	0	1	1	1	1
SHROOM4	frameshift_variant&start_lost	0	1	1	0	0	0	0
SHROOM4	start_lost&duplication	1	1	1	1	1	1	1
SIM1	frameshift_variant	1	0	1	1	1	0	1
SIPA1L3&WDR87	bidirectional_gene_fusion	0	1	1	0	0	0	0
SIPA1L3	sequence_feature	1	0	0	1	1	0	1
SIX2&SIX3	bidirectional_gene_fusion	0	0	1	0	1	0	0
SLC18A3	sequence_feature	1	0	1	1	1	0	1
SLC24A1&VWA9	bidirectional_gene_fusion	0	1	0	0	0	0	0
SLC25A14&ZNF280C	bidirectional_gene_fusion	0	0	1	1	1	0	0
SLC25A27&TDRD6	gene_fusion	0	0	0	1	1	0	1
SLC29A1&SLC35B2	bidirectional_gene_fusion	0	0	1	0	0	0	1
SLC34A1	sequence_feature	0	0	0	1	1	0	0
SLC45A4	frameshift_variant&splice_region_vari	0	1	1	0	0	0	0
SLC46A2&ZFP37	gene_fusion	1	1	1	1	1	0	0
SLC46A2	frameshift_variant&splice_donor_vari	0	1	1	1	0	0	0
SLC4A10&TBR1	gene_fusion	1	0	1	0	0	1	1
SLC9A2	sequence_feature	0	0	1	0	1	0	0
SLC9A6	sequence_feature	0	0	0	0	0	0	1
SLITRK1&SLITRK6	gene_fusion	0	1	1	1	1	0	1
SLITRK1	frameshift_variant&stop_gained	0	0	1	0	0	0	0
SLITRK5	sequence_feature	0	1	0	0	0	0	0
SLITRK6	splice_acceptor_variant&splice_regior	0	0	1	0	1	0	0
SMC1B	frameshift_variant	0	1	0	0	0	0	0
SMG6	frameshift_variant&splice_acceptor_v	1	1	1	1	1	1	1
SMG8	sequence_feature	0	1	1	0	1	1	1
SON	sequence_feature	1	1	1	1	1	0	1
SORBS2	frameshift_variant&splice_acceptor_v	0	0	1	0	1	0	0
SOWAHB	frameshift_variant&stop_gained	1	1	1	0	1	0	1
SOX2	sequence_feature	0	0	0	0	0	0	1
SPATA24	frameshift_variant&stop_gained	1	0	0	1	1	0	1

SPATA31A1	sequence_feature	0	1	0	1	1	0	1
SPATA31A2	exon_region	0	1	0	1	1	0	1
SPATA31A6	stop_gained&duplication	1	1	1	1	1	1	1
SPATA31C2	non_coding_transcript_exon_variant	0	0	1	0	0	0	0
SPEN&ZBTB17	bidirectional_gene_fusion	0	1	0	0	0	0	1
SPERT	sequence_feature	0	0	0	0	0	1	1
SPHK1&UBE2O	bidirectional_gene_fusion	0	0	0	0	0	1	1
SPHKAP	duplication	1	1	0	1	1	0	0
SPHKAP	frameshift_variant	0	0	1	0	1	0	1
SPPL2C	sequence_feature	0	0	0	1	1	0	0
SPTB	frameshift_variant&splice_donor_vari	1	1	1	0	0	0	0
SPTY2D1	frameshift_variant&splice_acceptor_v	0	0	0	0	0	0	1
SPTY2D1	stop_gained&duplication	0	1	0	0	0	0	0
SRRM2	sequence_feature	0	1	1	1	1	1	1
SRSF1	protein_protein_contact	0	0	0	1	1	0	1
SSX2&SSX2B	bidirectional_gene_fusion	1	1	1	1	1	0	1
ST3GAL6	upstream_gene_variant	0	1	0	0	0	0	0
STAG2	protein_protein_contact	1	1	1	1	1	0	1
STARD8&YIPF6	gene_fusion	0	1	1	0	1	0	0
STARD8	sequence_feature	1	0	1	1	1	1	1
STARD9	sequence_feature	1	1	1	1	1	1	1
STK11IP	upstream_gene_variant	1	0	0	0	0	0	0
STRA8	intragenic_variant	1	0	0	0	0	0	0
STRA8	sequence_feature	1	0	1	1	1	0	1
STRC&STRCP1	gene_fusion	1	0	0	0	0	0	0
STRC	frameshift_variant	1	0	0	1	1	1	1
STRC	frameshift_variant&start_lost	0	0	0	1	0	1	1
STRC	splice_donor_variant&splice_region_v	0	0	0	0	1	0	1
STX19	splice_acceptor_variant&splice_regior	0	1	0	0	0	0	1
STX4&ZNF646	gene_fusion	1	1	1	0	0	1	1
SUN1	sequence_feature	0	0	0	0	0	1	1
SUV420H1	frameshift_variant	1	0	0	0	0	0	0
SYCP1	exon_loss_variant	0	0	0	0	0	1	1
SYNCRIP	frameshift_variant&stop_lost	0	0	0	0	0	0	1
SYNC	frameshift_variant&splice_acceptor_v	0	1	0	0	0	0	0
SYNE1	frameshift_variant	1	1	1	1	1	0	0
SYNE1	frameshift_variant&splice_donor_vari	0	1	1	1	1	0	1
SYNPO	sequence_feature	1	1	1	0	1	1	1
SYT10	duplication	0	0	0	0	0	0	1
SYTL2	frameshift_variant	0	0	0	1	1	1	1
SYTL2	splice_acceptor_variant&splice_donor	0	1	1	0	0	0	0

SYTL2	stop_gained&duplication	1	1	1	1	1	1	1
SZT2	sequence_feature	1	0	0	1	1	0	0
TAAR6&TAAR9	gene_fusion	1	1	1	0	0	0	1
TACC2	sequence_feature	1	1	0	0	0	0	0
TAF1C	frameshift_variant	1	1	1	0	1	1	1
TAOK2	sequence_feature	0	0	0	0	0	1	1
TARDBPP2&TDRD3	gene_fusion	0	0	1	1	1	0	1
TBC1D28&ZNF286B	gene_fusion	0	0	1	0	0	0	0
TBCD&ZNF750	bidirectional_gene_fusion	1	1	0	0	0	1	1
TBL3	sequence_feature	1	1	1	0	1	1	1
TCAIM&ZNF445	bidirectional_gene_fusion	0	0	0	0	0	1	1
TCEB3C&TCEB3CL2	gene_fusion	1	1	1	1	1	1	1
TCF20	start_lost&duplication&splice_region_	1	1	1	1	1	1	1
TCIRG1	sequence_feature	1	0	0	0	0	0	0
TDRD15	sequence_feature	1	1	1	1	1	0	1
TDRD6	sequence_feature	0	0	0	0	0	1	1
TECTA	sequence_feature	0	0	0	0	0	0	1
TEKT3&ZNF286A	bidirectional_gene_fusion	0	0	0	0	0	1	0
TET3	sequence_feature	1	1	1	1	1	0	1
TEX15	frameshift_variant	0	1	1	1	1	0	0
TEX15	splice_acceptor_variant&splice_regior	1	1	1	1	1	1	1
TEX15	stop_gained&duplication	0	1	0	0	0	0	0
THAP9	downstream_gene_variant	0	0	0	0	0	0	1
THAP9	sequence_feature	1	1	1	1	1	0	1
THEMIS	frameshift_variant	0	0	1	0	0	0	1
THOC2&XIAP	bidirectional_gene_fusion	1	0	0	0	0	0	0
TIAM1	frameshift_variant&start_lost&splice_	1	0	0	0	0	0	1
TIAM2	sequence_feature	0	1	0	0	0	0	0
TIE1&TMEM125	gene_fusion	1	1	1	1	1	0	0
TIGD4	duplication	0	0	1	1	1	0	0
TLE3	splice_donor_variant&duplication&spl	0	1	0	0	0	0	0
TLR5	frameshift_variant&start_lost&splice_	0	0	1	0	0	0	0
TLR7&TLR8	gene_fusion	0	0	0	0	0	1	1
TLR7	sequence_feature	0	1	1	1	1	0	0
TLR8	structural_interaction_variant	1	1	0	1	1	0	1
TLR9	frameshift_variant&stop_gained&splic	1	0	1	0	0	0	1
TM9SF2	frameshift_variant	0	0	1	0	0	0	0
TMEM132A	sequence_feature	0	0	1	0	0	0	0
TMEM14A	sequence_feature	1	1	1	1	1	1	1
TMTC2	sequence_feature	0	0	0	0	0	1	1
TMX2&TMX2-CTNND1	gene_fusion	0	0	0	0	0	0	1

TNC	frameshift_variant&start_lost	1	1	1	1	1	1	1
TNK2	frameshift_variant&splice_acceptor_v	0	0	1	0	1	0	0
TNKS1BP1	stop_gained&duplication	0	1	1	0	1	0	0
TNXB	frameshift_variant	1	1	1	1	1	0	1
TOPAZ1	sequence_feature	0	0	1	0	0	0	0
TOPORS&TOPORS-AS1	bidirectional_gene_fusion	1	1	0	1	1	0	0
TOPORS	stop_gained&duplication	0	0	0	0	0	0	1
TPTE	frameshift_variant&stop_gained	1	0	0	0	0	0	1
TRANK1	frameshift_variant	1	1	0	0	0	0	0
TRANK1	frameshift_variant&splice_acceptor_v	1	1	0	0	0	0	0
TRIM32	sequence_feature	0	0	0	0	0	0	1
TRIP11	frameshift_variant	0	0	0	0	0	1	1
TRIP11	frameshift_variant&splice_donor_vari	0	1	1	1	1	0	0
TRIP11	splice_donor_variant&duplication&spl	1	1	1	1	1	0	1
TRMT61B&WDR43	bidirectional_gene_fusion	1	0	1	0	0	0	0
TRO	sequence_feature	1	1	1	1	1	1	1
TRPS1	frameshift_variant	1	0	0	0	1	0	1
TSC22D1	frameshift_variant&start_lost&splice_	1	1	1	0	1	0	1
TSHZ3&URI1	bidirectional_gene_fusion	0	0	1	1	1	0	0
TSHZ3&ZNF536	bidirectional_gene_fusion	0	0	1	0	1	0	0
TSKU	sequence_feature	1	1	0	0	1	0	0
TSN	protein_protein_contact	0	0	0	1	1	0	0
TSPYL6	duplication	0	1	0	0	0	0	0
TTBK2	frameshift_variant	1	1	1	1	1	1	1
TTC28	frameshift_variant	0	0	0	0	0	0	1
TTC28	frameshift_variant&stop_lost	0	0	0	0	0	0	1
TTC30A&TTC30B	gene_fusion	1	1	0	0	0	0	1
TTN	duplication	1	1	1	0	1	1	1
TTN	frameshift_variant	0	0	0	1	1	1	1
TTN	stop_gained&duplication&splice_regic	1	1	1	1	1	1	1
TUBB6	sequence_feature	1	1	0	0	0	0	0
TUBGCP4&ZSCAN29	bidirectional_gene_fusion	0	1	0	0	0	0	0
TYW1B	frameshift_variant	1	1	1	0	0	1	1
UACA	frameshift_variant&splice_donor_vari	1	1	1	1	1	1	1
UBE3A	frameshift_variant	1	1	1	1	1	0	0
UBXN6	duplication	0	1	1	1	1	1	1
UBXN6	stop_gained&duplication	1	0	1	0	0	0	0
UGT1A9	frameshift_variant&splice_region_vari	1	1	1	1	1	1	1
UGT2B10&UGT2B15	bidirectional_gene_fusion	1	0	1	0	1	0	1
UHRF1BP1L	frameshift_variant&splice_acceptor_v	1	1	1	1	1	0	1
UIMC1	frameshift_variant	1	0	0	1	1	0	0

ULBP2	sequence_feature	1	1	1	1	1	0	0
UQCRFS1&ZNF254	bidirectional_gene_fusion	0	0	0	0	0	0	1
UQCRFS1&ZNF91	gene_fusion	1	1	1	1	1	0	0
URB2	sequence_feature	1	1	1	1	1	0	1
URGCP	frameshift_variant&stop_gained	0	0	0	1	1	0	0
USP12&WASF3	bidirectional_gene_fusion	1	0	1	1	1	0	0
USP12-AS1	transcript_ablation	0	0	0	1	1	0	1
USP17L10&USP17L11&USP17I	feature_ablation	0	1	0	0	0	0	1
USP27X-AS1	upstream_gene_variant	0	0	1	1	1	0	1
USP29&ZIM3	bidirectional_gene_fusion	1	1	1	1	1	1	1
USP35	sequence_feature	1	1	0	0	0	0	1
USP44	frameshift_variant&start_lost&splice_	1	1	0	0	0	0	0
USP6NL	frameshift_variant&stop_gained&splic	1	1	1	1	1	0	0
VARS	frameshift_variant&stop_gained	1	1	1	1	1	1	1
VASN	sequence_feature	0	1	1	1	1	0	0
VCAN&VCAN-AS1	bidirectional_gene_fusion	1	0	1	1	1	1	1
VCAN	frameshift_variant	0	1	1	0	0	0	1
VCPIP1	splice_donor_variant&duplication&spl	0	1	1	0	0	0	0
VCX2&VCX3B	bidirectional_gene_fusion	0	0	1	0	1	0	0
VIT	sequence_feature	1	0	0	0	0	0	0
VPRBP	frameshift_variant&splice_acceptor_v	0	0	0	0	0	0	1
WDR87	frameshift_variant&stop_gained	0	0	0	1	1	1	1
WDR87	frameshift_variant&stop_lost	0	1	1	0	0	0	0
XIRP1	frameshift_variant	0	0	0	1	1	0	0
XIRP1	splice_acceptor_variant&splice_regior	1	1	1	1	1	1	1
XIST	duplication	1	1	1	1	1	1	1
YTHDF1	splice_acceptor_variant&splice_donor	1	0	1	1	1	1	1
YWHAEP5	duplication	0	0	0	1	1	0	0
Z95704.1&ZNF595	duplication	1	1	1	1	1	0	1
ZADH2&ZNF407	bidirectional_gene_fusion	0	1	1	0	0	0	0
ZBED5	frameshift_variant&stop_gained&splic	0	1	1	1	1	0	1
ZBTB18	sequence_feature	0	1	1	0	1	0	0
ZBTB21	frameshift_variant&start_lost	1	1	1	0	0	0	1
ZBTB34&ZBTB43	gene_fusion	0	0	1	0	1	1	1
ZBTB34	exon_region	1	0	0	0	1	0	0
ZBTB5	frameshift_variant&stop_gained	0	1	0	0	0	0	0
ZC3H13	duplication	1	0	1	0	0	1	1
ZC3H13	frameshift_variant	0	1	0	1	1	1	1
ZC3H4	frameshift_variant	0	1	1	0	0	0	0
ZC3H4	frameshift_variant&stop_gained	1	1	1	0	1	0	0
ZDBF2	sequence_feature	1	1	1	1	1	1	1

ZDHHC11	splice_acceptor_variant&splice_regior	1	0	0	0	0	0	0
ZDHHC8	sequence_feature	0	1	1	0	1	0	0
ZEB2	frameshift_variant&stop_gained&splic	0	0	0	0	0	1	1
ZFP37	frameshift_variant	0	1	0	1	1	0	0
ZFP62	frameshift_variant&stop_gained&splic	0	0	0	0	0	1	1
ZHX1	splice_acceptor_variant&splice_regior	1	1	1	0	1	0	1
ZHX3	frameshift_variant&splice_donor_vari	1	1	1	1	1	0	1
ZKSCAN2	frameshift_variant	0	0	0	0	0	0	1
ZMYM1	sequence_feature	0	1	0	0	0	0	1
ZNF106	frameshift_variant&start_lost	1	0	1	1	1	1	1
ZNF148	frameshift_variant&stop_gained&splic	1	1	1	1	1	0	1
ZNF208&ZNF98	gene_fusion	1	0	0	1	1	0	0
ZNF208	frameshift_variant&stop_gained&splic	0	0	0	1	0	0	1
ZNF285	stop_gained&duplication	0	0	1	0	1	0	0
ZNF292	sequence_feature	0	1	1	0	0	0	0
ZNF318	stop_gained&duplication&splice_regic	0	0	1	1	1	0	0
ZNF326	sequence_feature	0	1	0	0	0	0	1
ZNF331&ZNF845	gene_fusion	0	0	1	0	0	0	0
ZNF334	frameshift_variant&stop_lost	1	0	1	1	1	0	1
ZNF354A	frameshift_variant&splice_acceptor_v	0	0	0	1	1	0	0
ZNF417&ZNF552	gene_fusion	0	0	0	1	1	0	0
ZNF423	frameshift_variant	1	1	1	1	1	1	1
ZNF423	stop_gained&duplication	0	0	0	1	0	1	1
ZNF425&ZNF786	gene_fusion	0	1	1	0	1	0	0
ZNF430&ZNF714	gene_fusion	0	0	0	0	0	1	1
ZNF438	frameshift_variant	1	1	1	1	1	1	1
ZNF438	frameshift_variant&start_lost	0	0	0	1	1	0	0
ZNF462	sequence_feature	0	1	0	0	0	0	0
ZNF469	sequence_feature	1	1	1	1	1	1	1
ZNF510	frameshift_variant&splice_region_vari	0	1	0	0	0	0	0
ZNF510	frameshift_variant&stop_gained&splic	0	0	1	0	0	0	0
ZNF518A	downstream_gene_variant	1	1	0	1	1	1	1
ZNF518B	duplication	0	1	1	1	1	0	0
ZNF518B	frameshift_variant	1	1	1	1	1	1	1
ZNF518B	sequence_feature	0	0	0	0	0	1	1
ZNF521	frameshift_variant&splice_acceptor_v	1	1	1	1	1	1	1
ZNF541	frameshift_variant	0	0	0	0	0	0	1
ZNF541	frameshift_variant&splice_donor_vari	1	1	0	0	1	0	0
ZNF541	stop_gained&duplication	1	1	0	0	0	0	0
ZNF562&ZNF846	gene_fusion	1	1	1	0	0	0	0
ZNF585A	frameshift_variant&splice_acceptor_v	1	1	0	0	0	0	1

ZNF585B	frameshift_variant&stop_gained	0	0	0	0	0	0	1
ZNF594	frameshift_variant&stop_gained	1	1	1	1	1	0	1
ZNF608	frameshift_variant	1	0	1	1	1	0	0
ZNF608	frameshift_variant&stop_gained&splic	0	0	0	0	0	0	1
ZNF611	frameshift_variant&stop_gained&splic	0	1	0	0	0	0	0
ZNF644	frameshift_variant	1	1	1	1	1	1	1
ZNF646&ZNF668	bidirectional_gene_fusion	0	0	1	1	1	0	0
ZNF658B	splice_acceptor_variant&splice_regior	0	0	0	1	1	0	0
ZNF658B	splice_region_variant&non_coding_tra	0	0	1	0	0	0	0
ZNF658	frameshift_variant	0	0	0	1	1	0	1
ZNF658	frameshift_variant&stop_lost	0	1	0	0	0	0	0
ZNF674	frameshift_variant&stop_lost	0	1	1	0	0	0	0
ZNF675&ZNF681	gene_fusion	0	0	0	0	0	0	1
ZNF676	frameshift_variant	0	0	0	0	0	0	1
ZNF687	sequence_feature	1	1	1	1	1	0	1
ZNF695	frameshift_variant&splice_donor_vari	0	0	1	1	1	0	0
ZNF721&ZNF876P	bidirectional_gene_fusion	1	0	0	0	0	0	0
ZNF721	frameshift_variant&splice_acceptor_v	0	0	0	1	1	0	0
ZNF721	frameshift_variant&stop_gained	0	0	1	0	0	0	0
ZNF724P&ZNF99	gene_fusion	1	1	0	1	1	0	0
ZNF729&ZNF98	bidirectional_gene_fusion	0	0	0	1	1	0	1
ZNF782	frameshift_variant	0	1	1	0	0	0	0
ZNF786	frameshift_variant&stop_gained	0	1	1	0	1	0	0
ZNF800	frameshift_variant&stop_gained&splic	1	1	1	1	1	0	1
ZNF831	frameshift_variant	1	1	1	1	1	1	1
ZNF850	duplication	1	1	0	0	0	0	1
ZNF850	frameshift_variant	0	1	0	0	0	0	0
ZNF91	frameshift_variant	0	0	0	0	0	0	1
ZNF92	sequence_feature	1	1	1	1	1	0	1
ZNF99	frameshift_variant	1	1	0	1	1	0	1
ZNF99	frameshift_variant&splice_donor_vari	0	1	0	1	1	0	0
ZRANB1	stop_gained&duplication	0	0	1	0	1	0	0
ZSWIM1&ZSWIM3	gene_fusion	0	1	1	1	1	0	0
ZXDA&ZXDB	bidirectional_gene_fusion	1	1	1	1	1	1	1
ZXDA	duplication	0	1	0	0	0	0	0
ZXDB	sequence_feature	0	1	1	0	0	0	0
ZZZ3	frameshift_variant&splice_donor_vari	0	0	0	0	0	0	1
ZZZ3	splice_acceptor_variant&splice_donor	0	1	0	0	0	0	0
