# Supplementary material for: Human sensorimotor organoids derived from healthy and amyotrophic lateral sclerosis stem cells form neuromuscular junctions
Source: Nat Commun. 2021 Aug 6;12:4744. doi: 10.1038/s41467-021-24776-4 (PMC8346474; doi:10.1038/s41467-021-24776-4)
Supplement: Supplementary file 2 — Description of Additional Supplementary Files [file 41467_2021_24776_MOESM2_ESM.docx]

Description of Additional Supplementary Files

Title: Supplementary Movie 1

Description: Outward migration of cells from within a seeded sphere.

Title: Supplementary Movie 2

Description: Example of skeletal muscle contraction.

Title: Supplementary Movie 3

Description: Tracking of optogenetic-induced muscle contractions.

Title: Supplementary Movie 4

Description: Optic flow analysis of spontaneous skeletal muscle contractions.
