# Supplementary material for: Human sensorimotor organoids derived from healthy and amyotrophic lateral sclerosis stem cells form neuromuscular junctions
Source: Nat Commun. 2021 Aug 6;12:4744. doi: 10.1038/s41467-021-24776-4 (PMC8346474; doi:10.1038/s41467-021-24776-4)
Supplement: Supplementary file 7 — Reporting Summary [file 41467_2021_24776_MOESM7_ESM.pdf]

nature research

Corresponding author(s): Brian Wainger

Last updated by author(s): 06-02-2021

Reporting Summary

Nature Research wishes to improve the reproducibility of the work that we publish. This form provides structure for consistency and transparency in reporting. For further information on Nature Research policies, see our Editorial Policies and the Editorial Policy Checklist.

Statistics

For all statistical analyses, confirm that the following items are present in the figure legend, table legend, main text, or Methods section.

n/a	Confirmed	
[ ]	[x]	The exact sample size (*n*) for each experimental group/condition, given as a discrete number and unit of measurement
[ ]	[x]	A statement on whether measurements were taken from distinct samples or whether the same sample was measured repeatedly
[ ]	[x]	The statistical test(s) used AND whether they are one- or two-sided<br>*Only common tests should be described solely by name; describe more complex techniques in the Methods section.*
[x]	[ ]	A description of all covariates tested
[ ]	[x]	A description of any assumptions or corrections, such as tests of normality and adjustment for multiple comparisons
[ ]	[x]	A full description of the statistical parameters including central tendency (e.g. means) or other basic estimates (e.g. regression coefficient) AND variation (e.g. standard deviation) or associated estimates of uncertainty (e.g. confidence intervals)
[ ]	[x]	For null hypothesis testing, the test statistic (e.g. *F*, *t*, *r*) with confidence intervals, effect sizes, degrees of freedom and *P* value noted<br>*Give P values as exact values whenever suitable.*
[x]	[ ]	For Bayesian analysis, information on the choice of priors and Markov chain Monte Carlo settings
[x]	[ ]	For hierarchical and complex designs, identification of the appropriate level for tests and full reporting of outcomes
[x]	[ ]	Estimates of effect sizes (e.g. Cohen's *d*, Pearson's *r*), indicating how they were calculated

Our web collection on statistics for biologists contains articles on many of the points above.

Software and code

Policy information about availability of computer code

Data collection	For patch clamp, PatchMaster software (PatchMaster v2x90.2, HEKA) was used for acquisition. For calcium imaging, we used Nikon's NIS Elements (4.51.01). For muscle contraction analysis Gen5 (v3.00, Biotek) was used with a Cytation5 instrument. For the confocal imaging we used MetaXpress (6.5.4.532). FACS sorting was gated with FACSDiva (v.6.1.3).
Data analysis	Custom code for MATLAB (MATLAB R2018b), the Seurat R package (v 2.3.4) in R version 3.5, and Fiji (ImageJ 1.52p) and the web interface of SPRING software (SPRING viewer 1.6) were used for data analysis. Relevant code used throughout this manuscript is available at Github, and a copy is preserved in Zenodo (https://zenodo.org/record/4893797#.YLeZPS1h1bU ; doi:10.5281/zenodo.4893797). Multiple component analysis and clustering were completed using the FactoMineR (v. 2.3) and pheatmap (v. 1.0.12) R packages in R (v. 3.5.0).

For manuscripts utilizing custom algorithms or software that are central to the research but not yet described in published literature, software must be made available to editors and reviewers. We strongly encourage code deposition in a community repository (e.g. GitHub). See the Nature Research guidelines for submitting code & software for further information.

Data

Policy information about availability of data

All manuscripts must include a data availability statement. This statement should provide the following information, where applicable:

- Accession codes, unique identifiers, or web links for publicly available datasets
- A list of figures that have associated raw data
- A description of any restrictions on data availability

The single-cell RNA-seq and whole exome sequencing data generated in this study have been deposited in the db-GaP database under accession code phs002440.v1.p1 [http://www.ncbi.nlm.nih.gov/projects/gap/cgi-bin/study.cgi?study_id=phs002440.v1.p1]. These data are available under restricted access to comply with the conditions of the informed consent form, access can be obtained by applying for controlled access.

CRISPR-Cas9 edited iPSC lines carrying the ALS associated mutations TDP+/G298S, PFN1+/G118V, SOD1+/G85R, and paired isotype controls are available upon request.

Field-specific reporting

Please select the one below that is the best fit for your research. If you are not sure, read the appropriate sections before making your selection.

- [x] Life sciences
- [ ] Behavioural & social sciences
- [ ] Ecological, evolutionary & environmental sciences

For a reference copy of the document with all sections, see nature.com/documents/nr-reporting-summary-flat.pdf

Life sciences study design

All studies must disclose on these points even when the disclosure is negative.

Sample size

Sample sizes were not pre-determined. We selected sample sizes according to existing examples in the field.

Data exclusions

Raw sequence reads were processed using the bcbio-nextgen single-cell RNA-seq pipeline (https://bcbio-nextgen.readthedocs.io/en/latest/contents/pipelines.html#single-cell-rna-seq). The pipeline uses tools from the umis repository (https://github.com/vals/umis) to generate a cell by gene count matrix. FASTQ files were formatted to parse out non-biological segments of the reads (i.e. cellular barcode, sample barcodes, and UMIs). Excess cellular barcodes were removed to reduce artifacts. The reads were aligned to the GRCh38 Ensembl Release 90 transcriptome with RapMap 84. Duplicate UMIs were collapsed and the number of reads per transcript were counted for each cellular barcode. Samples were assessed for quality and filtered using the distributions of reads per cell, UMIs per cell, genes per cell, mitochondrial ratios per cell, UMIs vs. genes detected, UMIs vs. read counts, and novelty scores.
Cell clustering was performed using the Seurat R package (v 2.3.4)23,85 in R(3.5). Cells with less than 200 unique genes were removed from the analysis, and genes expressed in less than five cells were filtered out. Raw expression values were log normalized and each gene was scaled and centered after regression of contributions from batch, cell cycle phase, total number of reads, and number of mitochondrial genes.
SPRING analysis was performed using the webtool35: number of PCA dimensions chosen was 50, with gene filtering settings of a minimum count of 3, a variability percentile threshold of 80%, and 5 nearest neighbors. No minimum was applied for cell filtering and a minimum of 3 cells was used for gene filtering.

These are standard filtering criteria for single-cell RNA-seq, as is the exclusion of cells with less than 200 unique genes.

Replication

All experiments were ran at independent times (n reported per experiment) and results such as contractions were confirmed across all lines in independent differentiations. Experiments were conducted in a minimum of 3 biological replicas (independent differentiations).

Randomization

Randomization was not used because experiments generally did not involve treatment assignment. In the small number of experiments involving treatment of wells from an individual iPSC line with drugs, assignment was done in a blind, arbitrary manner.

Blinding

Experimental acquisition and analysis were all performed with full blinding.

Reporting for specific materials, systems and methods

We require information from authors about some types of materials, experimental systems and methods used in many studies. Here, indicate whether each material, system or method listed is relevant to your study. If you are not sure if a list item applies to your research, read the appropriate section before selecting a response.

Materials & experimental systems

n/a	Involved in the study
[ ]	[x] Antibodies
[ ]	[x] Eukaryotic cell lines
[x]	[ ] Palaeontology and archaeology
[x]	[ ] Animals and other organisms
[x]	[ ] Human research participants
[x]	[ ] Clinical data
[x]	[ ] Dual use research of concern

Methods

n/a	Involved in the study
[x]	[ ] ChIP-seq
[ ]	[x] Flow cytometry
[x]	[ ] MRI-based neuroimaging

Antibodies

Antibodies used

The list of antibodies used is as follows: B-III Tubulin (1:500, Abcam ab18207), Bassoon (1:100, Abcam ab110426), BRN3A (1:200, Millipore MAB1585), GFAP (1:200,

Sigma-Aldrich G9269), HB9 (1:100, DSHB 81.5C10-c), IBA1 (1:200, Wako Pure Chemicals 019-19741), IBA1 (1:50, Abcam Ab107159), ISL1/2 (1:50, DSHB
39.4D5), PAX7 (1:500, ThermoFisher Scientific PA1-117), Peripherin (1:100, Millipore AB1530), S100" (1:200, Abcam ab52642), SMI32 (1:200, BioLegend 801701), SOX2 (1:100, Abcam ab97959), TBXT (1:200, R&D AF2085), TUJ1 (1:500, BioLegend 801202), SAA (1:200, Abcam ab9465), "-III Tubulin (1:500, Abcam ab18207), APC anti-human CD56 (NCAM) Antibody (1:20, Biolegend 362503), APC Mouse IgG1 # Isotype Ctrl (FC) Antibody (1:20, Biolegend 400121), Human Fc Receptor Binding Inhibitor Purified 100 tests antibody (Thermo Fisher Scientific 14-9161-73), ChAT (1:200, EMD Millipore AB144P).

Validation

All antibodies were validated by the suppliers. Bassoon (Abcam, ab110426) detects multiple isoforms of Bassoon of mice, rats and humans by western blotting and immunohistochemistry of both frozen and paraffin sections (https://www.abcam.com/bassoonbsn-antibody-ab110426.html#top-239).
B-III Tubulin (Abcam ab18207) is a rabbit polyclonal antibody developed human B-III Tubulin. It detects
B-III Tubulin by immunohistochemistry, flow cytometry, and western blot in Mouse, Rat, Human, Common marmoset. https://www.abcam.com/beta-iii-tubulin-antibody-neuronal-marker-ab18207.html
BRN3A (POU4F1, Millipore, MAB1585, clone 5A3.2) has been validated for use in both immunohistochemistry and western blotting of chicken, monkey and rat (https://www.emdmillipore.com/US/en/product/Anti-Brn-3a-Antibody-POU-domain-proteinclone-
5A3.2,MM_NF-MAB1585?ReferrerURL=https%3A%2F%2Fwww.google.com%2F&bd=1). It has been validated in literature for use with human iPSCs (https://www-nature-com.ezp-prod1.hul.harvard.edu/articles/srep30552).
GFAP (Sigma-Aldrich G9269), has been validated for detection of astrocytes in western blot, immunohistochemistry and microarray applications of both rat and human samples (https://www.sigmaaldrich.com/catalog/product/sigma/g9269?lang=en&region=US).
HB9 (DSHB, 81-5C10) is an antibody developed against a purified internal fragment of mouse HB9 (MNR2 NH2(C-terminus; chick)-GST fusion protein expressed in E. coli). It detects HB9 by immunohistochemistry in Fish, Human, Mouse, Zebrafish. https://dshb.biology.uiowa.edu/81-5C10
IBA1 (Wako Pure Chemicals 019-19741) is an antibody against a synthethic peptide matching the C-terminal of the IBA1 protein, commonly used to detect microglia by immunohistochemistry and western blotting of mouse, rat, human and other species (https://labchem-wako.fujifilm.com/us/category/01213.html).
IBA1 (Abcam Ab107159) was used only when species compatibility issues arose with other antibodies and the aforementioned Wako IBA1 antibody. It is predicted to work with rat (https://www.abcam.com/iba1-antibody-ab107159.html), but publications suggest it works with human samples https://www.abcam.com/iba1-antibody-ab107159-references.html.
ISL1/2 (DSHB 39.4D5) is an antibody developed against the amino acid sequence 178-349 of ISL1 that also detects ISL2 and has been noted to work in immunohistochemistry of paraffin and paraformaldehyde fixed tissue of a variety of species including mouse, rat and human https://dshb.biology.uiowa.edu/39-4D5.
PAX7 (ThermoFisher Scientific PA1-117) is an antibody developed against a purified internal fragment of human PAX7. It detects PAX7 by immunohistochemistry and western blot in both human and mouse https://www.thermofisher.com/antibody/product/PAX7-Antibody-Polyclonal/PA1-117.
Peripherin (Millipore AB1530) has been validated for the detection of Peripherin by immunohistochemistry and western blot in human, rat, mouse and other species https://www.emdmillipore.com/US/en/product/Anti-Peripherin-Antibody,MM_NFAB1530.
S100" (Abcam ab52642) antibody has been validated for immunohistochemistry, western blot and immunoprecipitation in human, rat, mouse, and other species https://www.abcam.com/s100-beta-antibody-ep1576y-ab52642.html.
SMI32 (BioLegend 801701) antibody has been validated to detect a non-phosphorilated version of neurofilament heavy (NF-H) in immunohistochemistry, western blot and array tomography of human, rat, and mouse samples https://www.biolegend.com/enus/products/purified-anti-neurofilament-h-nf-h--nonphosphorylated-antibody-11475 .
SOX2 (Abcam ab97959) has been validated in immunohistochemistry and western blot of Mouse, Rat, Human, and other species https://www.abcam.com/sox2-antibody-ab97959.html.
TBXT (R&D AF2085) detects Brachyury by Elisa and Western blot of mouse and human https://www.rndsystems.com/products/human-mouse-brachyury-antibody_af2085 and has been used in immunohistochemistry of human samples https://www-ncbinlm-nih-gov.ezp-prod1.hul.harvard.edu/pmc/articles/PMC4624992/.
SAA (Abcam ab9465) is an antibody raised against full length rabbit sarcomeric alpha actinin. Validated for immunohistochemistry and western blot of human, rat, mouse, and other species https://www.abcam.com/sarcomeric-alphaactinin-antibody-ea-53-ab9465.html.
TUJ1 (BioLegend 801202) detects an epitope of TUBB3. Validated for immunohistochemistry, western blot and flow cytometry https://www.biolegend.com/en-us/products/purified-anti-tubulin-beta-3-tubb3-antibody-11580.
"-III Tubulin (Abcam ab18207) is an antibody raised against an immunogen that shares 75% homology withTUBB3. Validated for immunohistochemistry, western blot and flow cytometry in mouse, rat, human and other species https://www.abcam.com/betaiii-tubulin-antibody-ab18207.html.
APC anti-human CD56 (NCAM) Antibody (Biolegend 362503) is a mouse -raised antibody against NCAM directly conjugated with APC. Tested for flow cytometry. https://www.biolegend.com/en-us/products/apc-anti-human-cd56-ncam-antibody-9941
APC Mouse IgG1 # Isotype Ctrl (FC) Antibody (1:20, Biolegend 400121) is the matching isotype control for APC anti-human CD56 (NCAM) Antibody (Biolegend 362503) which has been validated for flow cytometry applications https://www.biolegend.com/enus/products/apc-mouse-igg1--kappa-isotype-ctrl-fc-3034.
Human Fc Receptor Binding Inhibitor Purified 100 tests antibody (Thermo Fisher Scientific 14-9161-73) is used to inhibit the nonspecific
binding of mice-raised antibodies by the human Fc-gamma receptor (FcgammaR) https://www.thermofisher.com/antibody/product/Fc-Receptor-Binding-Inhibitor-Antibody-Polyclonal/14-9161-73.
ChAT (EMD Millipore AB144P) is an antibody against Choline Acetyltransferase that has been validated for use in immunohistochemistry and western blot of species including human, rat and mouse https://www.emdmillipore.com/US/en/

product/Anti-Choline-Acetyltransferase-Antibody,MM_NF-AB144P.

Eukaryotic cell lines

Policy information about cell lines

Cell line source(s)	The FA0000011 and FA0000012 lines were obtained through NINDs (https://nindsgenetics.org/target-als-project). The 11a, 19f, MGH5b, and HuES3 lines were kindly provided by Dr. Kevin Eggan (https://www.ncbi.nlm.nih.gov/pubmed/21293464 and https://www.ncbi.nlm.nih.gov/pubmed/24703839). Isogenic pairs of mutant and control lines for ALS related genes (TDP-43 G298S, PFN1 G118V, and SOD1 G85R, were generated in collaboration with the IPSC core of the Harvard Stem Cell Institute.
Authentication	The iPSC nature of the cell lines was confirmed by morphological analysis and differentiation potential.
Mycoplasma contamination	All cell lines tested negative for mycoplasma contamination.
Commonly misidentified lines (See ICLAC register)	No common misidentified lines were used.

Flow Cytometry

Plots

Confirm that:

- [x] The axis labels state the marker and fluorochrome used (e.g. CD4-FITC).
- [x] The axis scales are clearly visible. Include numbers along axes only for bottom left plot of group (a 'group' is an analysis of identical markers).
- [x] All plots are contour plots with outliers or pseudocolor plots.
- [x] A numerical value for number of cells or percentage (with statistics) is provided.

Methodology

Sample preparation	Dissociation of the organoid culture was done at week four by incubating the culture in 0.25% Trypsin-EDTA (Thermo Fisher Scientific, 25200056) for 20 minutes at 37°C. The resulting cell suspension was dissociated mechanically by pipetting ten times, followed by centrifugation at 200 rcf for 5 minutes. Briefly, the cell pellet was resuspended into 200 µl of Fc-block antibody solution (1:100 of Human Fc Receptor Binding Inhibitor, Purified, Thermo Fisher Scientific, 14-9161-73; in PBS with 2% FBS, GE Healthcare, SH30910.03HI) for 30 minutes at 4°C while protected from light. Directly conjugated antibodies (NCAM or IG control) were then diluted in PBS with 2%FBS. The cell suspension was split in a 9:1 ratio, and 10% was reserved as IG control. The antibody solutions were either added to 180 µl (NCAM) or 20 µl (IG) of the cell suspension and were incubated protected from light for 30 minutes at 4°C. Finally, the cells were washed twice by adding PBS with 2% FBS and centrifuging at 200 rcf for five minutes before sorting108.
Instrument	FACS sorting was performed with either a BD FACSAria II or a BD FACSAria Fusion54.
Software	The software used was FACSDiva v.6.1.3. for the BD FACSAria II or a FACSDiva v.8.0.0 for the BD FACSAria Fusion.
Cell population abundance	We found that NCAM-based FACS selected 23.0 ± 7.1% (mean ± S.E.M.) of cells (Fig. 3c and Extended Data Fig. 3). Given that NCAM can label other, non-neuronal cell types, we subsequently stained for TUJ1 (Fig. 3d) which revealed that 38.0 ± 1.3% of the NCAM-purified cells (mean ± S.E.M., Fig. 3d) were neuronal. Overall, 8.7 ± 2.7% of total cells were NCAM- and TUJ1-positive neurons (mean ± S.E.M.), in agreement with the 8.5% of cells in neuronal clusters identified by single-cell RNA-seq (Supplementary Table 1).
Gating strategy	The gates were defined by using a fraction of the sample as a paired negative control. This control was labeled with the matching IgG Isotype directly conjugated with APC. FSC-H and FSC-A, as well as FSC-A and SSC-A were used to exclude doublets and debris. Hoescht staining, detectable in the Pacific Blue channel, was used to detect live cells. The gates for selection of positive cells were set such that they exclude all events in the negative control.

- [x] Tick this box to confirm that a figure exemplifying the gating strategy is provided in the Supplementary Information.
